# Microwave-assisted cyclizations promoted by polyphosphoric acid esters: a general method for 1-aryl-2-iminoazacycloalkanes

**DOI:** 10.3762/bjoc.12.190

**Published:** 2016-09-14

**Authors:** Jimena E Díaz, María C Mollo, Liliana R Orelli

**Affiliations:** 1 Universidad de Buenos Aires. CONICET. Departamento de Química Orgánica. Facultad de Farmacia y Bioquímica. Junín 956, (1113) Buenos Aires, Argentina

**Keywords:** cyclic amidines, medium size heterocycles, microwaves, PPE, PPSE

## Abstract

The first general procedure for the synthesis of 5 to 7-membered 1-aryl-2-iminoazacycloalkanes is presented, by microwave-assisted ring closure of ω-arylaminonitriles promoted by polyphosphoric acid (PPA) esters. 1-Aryl-2-iminopyrrolidines were easily prepared from the acyclic precursors employing a chloroformic solution of ethyl polyphosphate (PPE). The use of trimethylsilyl polyphosphate (PPSE) in solvent-free conditions allowed for the synthesis of 1-aryl-2-iminopiperidines and hitherto unreported 1-aryl-2-iminoazepanes. The cyclization reaction involves good to high yields and short reaction times, and represents a novel application of PPA esters in heterocyclic synthesis.

## Introduction

The synthesis of new nitrogen heterocycles has a great interest in medicinal chemistry since they are part of many drugs and represent structures with a wide therapeutic potential [1–2]. 2-Iminoazacycloalkanes are cyclic amidines where the formally sp^2^ nitrogen is exocyclic. These compounds have been described as selective inhibitors of the inducible form of human nitric oxide synthase (iNOS), which catalyzes the reaction to form nitric oxide via the oxidation of L-arginine to L-citrulline [3–6]. Some urea and thiourea derivatives have been studied as CNS agents [7] and anthelmintic drugs [8]. This heterocyclic core therefore represents the foundation for potential bioactive agents.

A few methods have been described for the synthesis of 2-iminoazacycloalkanes from acyclic precursors. One strategy involves the cyclization of ω*-*aminonitriles, which requires the presence of protic acids [9–10]. It has also been described the reaction between halonitriles and primary or secondary amines, where the ω*-*aminonitrile is formed in situ [8,11–14]. This heterocyclic core has also been prepared by cyclization of azidonitriles [15]. These methods usually require drastic reaction conditions and are generally limited to 2-iminopyrrolidines and/or to *N*-unsubstituted or *N*-alkyl derivatives. Other methods involve cyclic precursors such as lactams [4,6–7,16], thiolactams [3,6] or 2-aminopyridines [6].

Literature examples on the corresponding *N-*unsubstituted or *N-*alkyl seven membered heterocycles, i.e. 2-iminoazepanes [3,7,9–10], are very scarce, and no general method has been described for their synthesis. To our knowledge, *N-*aryl derivatives have not been hitherto reported. The lack of synthetic methods can be attributed to the intrinsic difficulty of cyclizations leading to seven membered heterocycles. This is a consequence of the high activation energies involved in these reactions, which are also hindered by entropic factors [17–22]. In addition to this, due to the considerably lower nucleophilicity of the arylamino group, procedures for *N*-alkyl or *N-*unsubstituted 2-iminoazacycloalkanes are usually not suitable for the synthesis of *N*-aryl derivatives.

Ethyl polyphosphate (PPE) and trimethylsilyl polyphosphate (PPSE) are mild irreversible dehydrating agents of the Lewis acid type. They have been widely used for several synthetically useful transformations like dehydration of amides leading to nitriles [23–24] or the Beckmann rearrangement [23,25]. They have also been employed in the synthesis of heterocycles such as benzimidazoles, benzothiazoles, benzoxazoles [23,26–28], indoles and 3,4-dihydroisoquinolines [23,29]. Microwave-assisted organic reactions proceed in general faster, with higher yields and more efficiently than those performed under conventional heating [30–38]. This technology has found interesting applications in heterocyclic synthesis, specially in cyclocondensation reactions [39–40]. In particular, it has enabled the synthesis of the more challenging medium size (7–9 membered) heterocycles by overcoming limitations such as low reactivity and yields, harsh reaction conditions and side reactions typical of conventional heating [17].

**Scheme 1:**
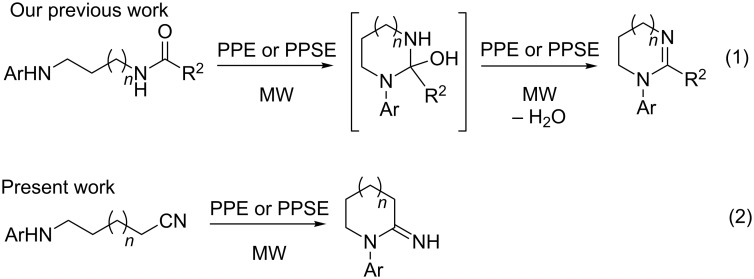
Our previous and present work.

In previous work, we reported the microwave-assisted synthesis of 5 to 8 membered cyclic amidines by cyclodehydration of *N*-aryl-*N´*-acyl-1,*n-*alkanediamines (*n* = 2–5) promoted by polyphosphoric acid (PPA) esters PPE and PPSE (Scheme 1, reaction 1) [41–43]. In this case, PPA esters activate the amidic oxygen and, at the same time, react chemically with water.

We hypothesized that applying similar reaction conditions to ω*-*arylaminonitriles could lead to 1-aryl-2-imino-1-azacycloalkanes (Scheme 1, reaction 2), providing a novel synthetic method for these heterocycles. In this case no dehydration would be involved and PPA esters, due to their Lewis acid nature, would solely increase the electrophilicity of the cyano group towards an intramolecular nucleophilic attack. This approach would avoid the use of strong protic acids, which may be disadvantageous for sensitive substrates.

## Results and Discussion

The ω*-*arylaminonitrile precursors were obtained by reaction of the corresponding ω-halonitrile and arylamines, as previously reported by our group [44].

We examined first the cyclization of 4-(*p*-tolylamino)butyronitrile (**1a**) with PPE under microwave irradiation in a closed vessel reactor. The reaction was completed after 5 minutes at 100 °C and 1-(*p*-tolyl)-2-iminopyrrolidine (**2a**) was obtained in 86% yield (Table 1, entry 1). No reaction occurred in the absence of PPE, while the use of classical Lewis acids (ZnCl_2_, AlCl_3_, BF_3_) as cyclization agents led to very low yields of the desired product **2a** (Table 1, entries 2–5).

Employing the optimized experimental conditions, 1-aryl-2-iminopyrrolidines **2b–h** were prepared in high yields (Table 1, entries 6–12).

**Table 1:** Synthesis of 1-aryl-2-iminopyrrolidines **2a–h.**

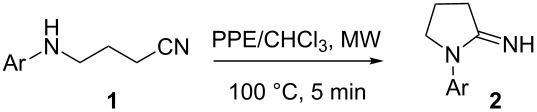			

Entry	Compound **2**	Ar	Yield (%)

1	**a**	4-CH_3_C_6_H_4_	86
2^a^	**a**	4-CH_3_C_6_H_4_	0
3^b^	**a**	4-CH_3_C_6_H_4_	traces
4^c^	**a**	4-CH_3_C_6_H_4_	11
5^d^	**a**	4-CH_3_C_6_H_4_	17
6	**b**	C_6_H_5_	85
7	**c**	4-FC_6_H_4_	74
8	**d**	4-ClC_6_H_4_	82
9	**e**	4-BrC_6_H_4_	77
10	**f**	2-CH_3_C_6_H_4_	77
11	**g**	2-ClC_6_H_4_	80
12^e^	**h**	2-OCH_3_C_6_H_4_	85

^a^The reaction was run in the absence of reagents (background reaction). ^b^The reaction was run using stoichiometric amount of ZnCl_2_. ^c^The reaction was run using stoichiometric amount of AlCl_3_. ^d^The reaction was run using stoichiometric amount of BF_3_. ^e^The reaction time was 20 minutes.

In order to extend the scope of the method, we investigated next the synthesis of 1-aryl-2-iminopiperidines **4**. The conversion of 5-(*p*-tolylamino)valeronitrile (**3a**) to 1-(*p*-tolyl)-2-iminopiperidine (**4a**) was chosen for the optimization of the reaction conditions (Table 2). In the conditions used for the lower homologues **2** (PPE/CHCl_3_, 100 °C), no reaction occurred after 5 minutes (Table 2, entry 1). Increasing the reaction time and/or temperature resulted in higher conversion, but the *N*-ethyl derivative **5a** was obtained along with the desired product (Table 2, entries 2 and 3). This side reaction was quite unexpected, although some examples in the literature show that PPE can act as an ethylating agent under certain conditions [45–46]. Using a dichloromethane solution of PPSE, compound **4a** was obtained exclusively (Table 2, entries 4–6), while working under solvent-free conditions further improved the yield (Table 2, entry 7).

**Table 2:** Reaction conditions screening for 1-(*p*-tolyl)-2-iminopiperidine (**4a**).

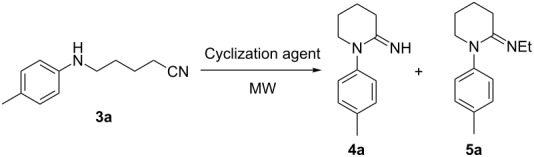						

Entry	Cyclization agent	Solvent	Temp. (°C)	Time (min.)	**4a** (%)	**5a** (%)

1	PPE	CHCl_3_	100	5	0	0
2	PPE	CHCl_3_	100	30	traces	traces
3	PPE	CHCl_3_	150	30	32	32
4	PPSE	CH_2_Cl_2_	130	15	34	0
5	PPSE	CH_2_Cl_2_	150	15	62	0
6	PPSE	CH_2_Cl_2_	150	30	71	0
7	PPSE	none	150	30	74	0

Employing the optimized reaction conditions (neat PPSE, 30 min at 150 °C), 1-aryl-2-iminopiperidines **4** were synthesized in high yields (Table 3). Compound **4g** required a higher reaction temperature (200 °C) due to steric hindrance of the *N*-aryl moiety.

**Table 3:** Synthesis of 1-aryl-2-iminopiperidines **4**.

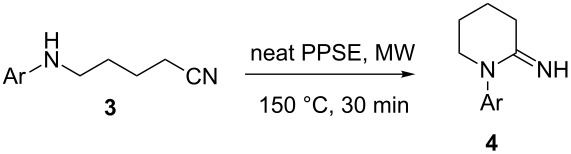			

Entry	Compound **4**	Ar	Yield (%)

1	**a**	4-CH_3_C_6_H_4_	74
2	**b**	C_6_H_5_	79
3	**c**	4-ClC_6_H_4_	72
4^a^	**d**	2-CH_3_C_6_H_4_	74
5	**e**	2-FC_6_H_4_	82
6^a^	**f**	2-ClC_6_H_4_	73
7^b^	**g**	2,6-(CH_3_)_2_C_6_H_4_	86

^a^The reaction time was 40 minutes. ^b^This reaction was carried out at 200 °C.

The encouraging results obtained up to this point, together with the absence of methods for the synthesis of the higher homologues 1-aryl-2-iminoazepanes **7** prompted us to attempt the microwave-assisted cyclization of 6-arylaminohexanenitriles **6** promoted by PPA esters. We examined first the cyclization of compound **6a** under different reaction conditions (Table 4). No conversion was observed using PPE/CHCl_3_ at 100 °C for 5 minutes (Table 4, entry 1). When longer reaction times and/or higher temperatures were used, 1-(*p*-tolyl)-2-ethyliminoazepane (**8a**) was obtained as the only product (Table 4, entries 2 and 3). The use of PPSE in DCM solution afforded traces of the desired product **7a** (Table 4, entry 4). Performing the reaction under solvent-free conditions at 150 °C, compound **7a** was obtained in modest yield (Table 4, entry 5). Significantly better results were achieved by increasing the temperature to 200 °C (Table 4, entry 6).

**Table 4:** Reaction conditions screening for 1-(*p*-tolyl)-2-iminoazepane (**7a**).

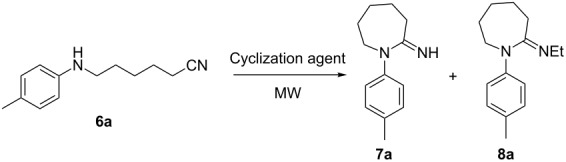						

Entry	Cyclization agent	Solvent	Temp. (°C)	Time (min)	**7a** (%)	**8a** (%)

1	PPE	CHCl_3_	100	5	0	0
2	PPE	CHCl_3_	100	30	0	10
3	PPE	CHCl_3_	150	30	0	77
4	PPSE	CH_2_Cl_2_	150	30	traces	0
5	PPSE	none	150	30	45	0
6	PPSE	none	200	30	73	0

Employing the optimized reaction conditions, we synthesized some novel 1-aryl-2-iminoazepanes **7** in good to high yields (Table 5). A clear steric effect on the reactivity was observed in the case of compound **7g** (Table 5, entry 7), which resulted in low conversion of the substrate.

**Table 5:** Synthesis of 1-aryl-2-iminoazepanes **7**.

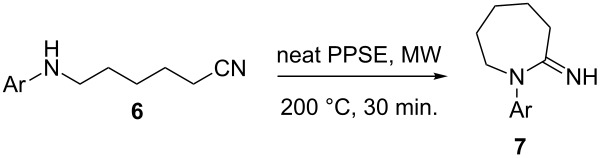			

Entry	Compound **7**	Ar	Yield (%)

1	**a**	4-CH_3_C_6_H_4_	73
2	**b**	C_6_H_5_	62
3	**c**	4-FC_6_H_4_	62
4	**d**	4-BrC_6_H_4_	75
5	**e**	2-CH_3_C_6_H_4_	55
6	**f**	2-FC_6_H_4_	68
7	**g**	2,6-(CH_3_)_2_C_6_H_4_	28

## Conclusion

In summary, we have developed a straightforward and efficient protocol for the microwave-assisted synthesis of 1-aryl-2-iminoazacycloalkanes, by ring closure of ω-arylaminonitriles promoted by PPA esters. This reaction constitutes a novel application of such reagents in heterocyclic synthesis. The procedure involves easily available starting materials and requires remarkably short reaction times. It affords the desired compounds in good to high yields and avoids the use of protic acids. Noteworthly, classical Lewis acids failed to efficiently promote this transformation. To our knowledge, this is the first method which allows the synthesis of the hitherto unreported *N*-aryl 7-membered heterocycles.

## Supporting Information

File 1 Experimental procedures, characterization of new compounds and copies of ^1^H and ^13^C NMR spectra.
