# Supplementary material for: Microwave-assisted cyclizations promoted by polyphosphoric acid esters: a general method for 1-aryl-2-iminoazacycloalkanes
Source: Beilstein J Org Chem. 2016 Sep 14;12:2026–31. doi: 10.3762/bjoc.12.190 (PMC5082496; doi:10.3762/bjoc.12.190)
Supplement: File 1 — Experimental procedures, characterization of new compounds and copies of 1H and 13C NMR spectra. [file Beilstein_J_Org_Chem-12-2026-s001.pdf]

Supporting Information

for

Microwave-assisted cyclizations promoted by polyphosphoric acid esters: a general method for 1-aryl-2-iminoazacycloalkanes

Jimena E. Díaz, María C. Mollo and Liliana R. Orelli*

Address: Universidad de Buenos Aires. CONICET. Departamento de Química Orgánica. Facultad de Farmacia y Bioquímica. Junín 956, (1113) Buenos Aires, Argentina.

Email: Liliana R. Orelli - lorelli@ffyb.uba.ar

*Corresponding author

Experimental procedures, characterization of new compounds and copies of ^{1}H and ^{13}C NMR spectra

1. General information S2
2. Representative procedures for synthesis S2
3. Characterization data for compounds **1–8** S3
4. Copies of ^{1}H and ^{13}C NMR spectra of compounds **1–8** S14
5. References S50

1. General information

Melting points were determined with a Büchi capillary apparatus and are uncorrected. ^{1}H and ^{13}C NMR spectra were recorded on a Bruker Bio Spin Avance III 600 MHz spectrometer, a Bruker Avance II 500 MHz spectrometer or a Bruker MSL 300 MHz spectrometer, using deuteriochloroform as the solvent. Chemical shifts are reported in ppm (δ) relative to TMS as an internal standard. D_2O was employed to confirm exchangeable protons (ex). Splitting multiplicities are reported as singlet (s), broad signal (bs), doublet (d), double doublet (dd), triplet (t), quartet (q), heptet (h) and multiplet (m). HRMS (ESI) were performed with a Bruker MicroTOF-Q II spectrometer. Reagents, solvents and starting materials were purchased from standard sources and purified according to literature procedures. PPE was prepared according to the procedure described by Schramm [1], without evaporation of the solvent. PPSE was prepared according to Yokoyama [2].

2. Representative procedures for synthesis

2.1. Representative procedure for the synthesis of 1-aryl-2-iminopyrrolidines **2**.

A mixture of the corresponding compound **1** (0.5 mmol) and a chloroform solution of PPE (4 mL) was reacted in a microwave reactor (Monowave 300, Anton Paar) at the indicated temperature and time. After reaching room temperature, the resulting solution was extracted with water (5 x 8 mL). The aqueous phases were pooled, filtered and made alkaline in an ice bath, and the mixture extracted with dichloromethane (3 x 40 mL). The organic layer was washed with water (5 mL), dried over sodium sulfate and filtered. The solvent was removed in vacuo. The crude products were purified by column chromatography (Silica gel 60, DCM:methanol:isopropylamine).

2.2. Representative procedure for the synthesis of 1-aryl-2-iminopiperidines **4** and 1-aryl-2-iminoazepanes **7**.

A mixture of the corresponding compound **3** or **6** (0.5 mmol) and neat PPSE (3 g) was reacted in the microwave reactor (Monowave 300, Anton Paar) at the indicated temperature and time. After reaching room temperature, the resulting oil was treated with dichloromethane (25 mL) and 10% aqueous NaOH (15 mL). The aqueous phase was extracted with dichloromethane (2 x 25 mL). The organic phases were pooled, washed with water (5 mL), filtered, dried over sodium sulfate and filtered. The solvent was removed in vacuo. The crude products were purified by column chromatography.

2.3. Representative procedure for the synthesis of 4-arylaminobutyronitriles **1**, 5-arylaminovaleronitriles **3** and 6-arylaminohexanenitriles **6**.

A solution of the corresponding precursor (4-chlorobutyronitrile for compounds **1**, 5-chlorovaleronitrile for compounds **3** and 6-bromohexanenitrile for compounds **6**) (2.5 mmol) in dimethylformamide (1 mL) was added during 1.5 h to a mixture of the arylamine (2.5 mmol), Cs_2CO_3 (2.5 mmol) and KI (5 mmol) in dimethylformamide (2.5 mL). The mixture was stirred at the indicated temperature and time. After completion of the reaction, as indicated by TLC, the mixture was treated with ethyl ether (50 mL) and water (10 mL). The aqueous phase was separated and extracted with ethyl ether (30 mL). The combined organic layers were dried over anhydrous sodium sulfate and filtered. The solvent was evaporated in vacuo. The crude product was purified by column chromatography (silica gel, hexane:DCM).

3. Characterization data for compounds **1–8**.

Compounds **1a–c**,**e–f**, **3b,d** [3], **1d, 3a** [4] and **3c** [5] were described in the literature.

1-(*p*-Tolyl)-2-iminopyrrolidine (**2a**)

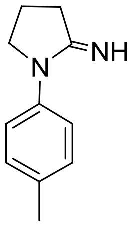

Yellow oil (86% yield). ^{1}H NMR (500 MHz, $CDCl_3$): δ 2.00-2.06 (m, 2H), 2.30 (s, 3H), 2.68 (t, *J*= 7.9 Hz, 2H), 3.74 (t, *J*= 6.8 Hz, 2H), 5.63 (bs, ex, 1H), 7.16 (dd, *J*= 8.6, 0.7 Hz, 2H), 7.33 (d, *J*= 8.6 Hz, 2H). ^{13}C NMR (125 MHz, $CDCl_3$): δ 20.1, 21.1, 33.9, 52.1, 122.0, 129.9, 134.1, 138.3, 167.5. HRMS (ESI) *m/z* calcd for $C_{11}H_{15}N_2$: 175.1230. Found: 175.1235.

1-Phenyl-2-iminopyrrolidine (**2b**)

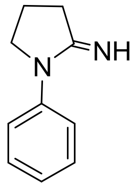

Yellow oil (85% yield). ^{1}H NMR (500 MHz, $CDCl_3$): δ 1.99-2.05 (m, 2H), 2.68 (t, *J*= 7.9 Hz, 2H), 3.76 (t, *J*= 6.9 Hz, 2H), 5.31 (bs, ex, 1H), 7.08 (tt, *J*= 7.4, 1.1 Hz, 1H), 7.34 (dd, *J*= 8.6, 7.4 Hz, 2H), 7.48 (dd, *J*= 8.6, 1.1 Hz, 2H). ^{13}C NMR (125 MHz, $CDCl_3$): δ 20.0, 34.1, 51.9, 121.6, 124.2, 129.2, 140.9, 167.6. HRMS (ESI) *m/z* calcd for $C_{10}H_{13}N_2$: 161.1073. Found: 161.1065.

1-(*p*-Fluorophenyl)-2-iminopyrrolidine (**2c**)

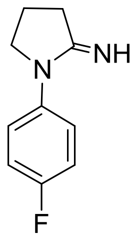

Yellow oil (74% yield). ^{1}H NMR (600 MHz, $CDCl_3$): δ 2.03-2.08 (m, 2H), 2.69 (t, *J*= 7.9 Hz, 2H), 3.76 (t, *J*= 6.9 Hz, 2H), 7.03-7.06 (m, 2H), 7.51 (dd, *J*= 8.1, 4.8 Hz, 2H). ^{13}C NMR (150 MHz, $CDCl_3$): δ 20.1, 34.3, 52.1, 115.9 (d, *J*= 22.0 Hz), 123.3 (d, *J*= 6.6 Hz), 137.3 (d, *J*= 2.2 Hz), 159.4 (d, *J*= 244.3 Hz), 167.9. HRMS (ESI) *m/z* calcd for $C_{10}H_{12}FN_2$: 179.0979. Found: 179.0983.

1-(*p*-Chlorophenyl)-2-iminopyrrolidine (**2d**)

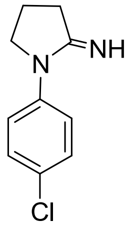

White solid (82% yield), mp: 88-90°C. ^{1}H NMR (500 MHz, $CDCl_3$): δ 2.02-2.07 (m, 2H), 2.69 (t, *J*= 7.8 Hz, 2H), 3.76 (t, *J*= 6.9 Hz, 2H), 5.59 (bs, ex, 1H), 7.29 (d, *J*= 8.9 Hz, 2H), 7.56 (d, *J*= 8.9 Hz, 2H). ^{13}C NMR (125 MHz, $CDCl_3$): δ 19.9, 34.6, 51.7, 122.1, 128.6, 129.1, 139.9, 167.9. HRMS (ESI) *m/z* calcd for $C_{10}H_{12}ClN_2$: 195.0684. Found: 195.0688.

1-(*p*-Bromophenyl)-2-iminopyrrolidine (**2e**)

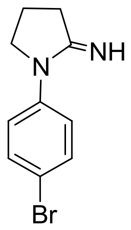

White solid (77% yield), mp: 83-85°C. ^{1}H NMR (600 MHz, $CDCl_3$): δ 2.04-2.10 (m, 2H), 2.71 (t, *J*= 7.9 Hz, 2H), 3.78 (t, *J*= 6.9 Hz, 2H), 7.46 (d, *J*= 8.9 Hz, 2H), 7.54 (d, *J*= 8.9 Hz, 2H). ^{13}C NMR (125 MHz, $CDCl_3$): δ 19.9, 34.7, 51.5, 116.1, 122.3, 132.0, 140.6, 167.9. HRMS (ESI) *m/z* calcd for $C_{10}H_{12}BrN_2$: 239.0178. Found: 239.0171.

1-(*o*-Tolyl)-2-iminopyrrolidine (**2f**)

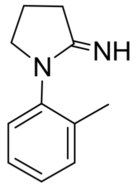

Yellow oil (77% yield). ^{1}H NMR (500 MHz, $CDCl_3$): δ 2.10-2.15 (m, 2H), 2.19 (s, 3H), 2.71 (t, *J*= 7.8 Hz, 2H), 3.65 (t, *J*= 6.8 Hz, 2H), 4.68 (bs, ex, 1H), 7.13-7.15 (m, 1H), 7.20-7.28 (m, 3H). ^{13}C NMR (125 MHz, $CDCl_3$): δ 17.9, 21.0, 32.2, 53.0, 127.5, 128.0, 128.1, 131.6, 136.8, 138.3, 167.8. HRMS (ESI) *m/z* calcd for $C_{11}H_{15}N_2$: 175.1230. Found: 175.1236.

1-(*o*-Chlorophenyl)-2-iminopyrrolidine (**2g**)

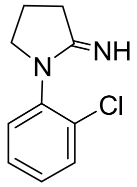

Yellow oil (80% yield). ^{1}H NMR (500 MHz, $CDCl_3$): δ 2.04-2.14 (m, 2H), 2.65 (t, *J*= 7.8 Hz, 2H), 3.66 (t, *J*= 6.7 Hz, 2H), 4.85 (bs, ex, 1H), 7.18-7.31 (m, 3H), 7.43 (d, *J*= 7.9 Hz, 1H). ^{13}C NMR (125 MHz, $CDCl_3$): δ 21.0, 32.2, 52.4, 128.2, 129.0, 130.2, 131.0, 133.3, 137.5, 168.0. HRMS (ESI) *m/z* calcd for $C_{10}H_{12}ClN_2$: 195.0684. Found: 195.0679.

1-(*o*-Methoxyphenyl)-2-iminopyrrolidine (**2h**)

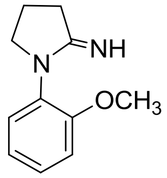

Yellow oil (85% yield). ^{1}H NMR (600 MHz, $CDCl_3$): δ 2.06-2.11 (m, 2H), 2.68 (t, *J*= 7.7 Hz, 2H), 3.68 (t, *J*= 6.8 Hz, 2H), 3.81 (s, 3H), 6.96-6.99 (m, 2H), 7.22 (dd, *J*= 7.7, 1.5 Hz, 1H), 7.26 (td, *J*= 7.9, 1.5, 1H). ^{13}C NMR (150 MHz, $CDCl_3$): δ 21.0, 32.4, 52.5, 55.9, 112.7, 121.4, 128.4, 128.8, 129.4, 156.0, 168.5. HRMS (ESI) *m/z* calcd for $C_{11}H_{15}N_2O$: 191.1179. Found: 175.1177.

1-(*p*-Tolyl)-2-iminopiperidine (**4a**)

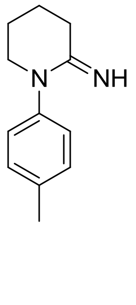

Yellow oil (74% yield). ^{1}H NMR (500 MHz, $CDCl_3$): δ 1.80-1.88 (m, 4H), 2.32 (s, 3H), 2.57 (t, *J*= 6.4 Hz, 2H), 3.42-3.44 (m, 2H), 5.13 (bs, ex, 1H), 7.06 (d, *J*= 8.1 Hz, 2H), 7.18 (d, *J*= 8.1 Hz, 2H). ^{13}C NMR (125 MHz, $CDCl_3$): δ 21.3, 22.3, 24.5, 32.6, 51.8, 127.2, 130.7, 136.8, 141.8, 164.4. HRMS (ESI) *m/z* calcd for $C_{12}H_{17}N_2$: 189.1386. Found: 189.1390.

1-(*p*-Tolyl)-2-ethyliminopiperidine (**5a**)

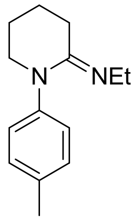

Yellow oil (32% yield). ^{1}H NMR (300 MHz, $CDCl_3$): δ 1.03 (t, *J*= 7.1 Hz, 3H), 1.84-1.85 (m, 4H), 2.30 (s, 3H), 2.48-2.52 (m, 2H), 3.23 (c, *J*= 7.1 Hz, 2H), 3.49-3.52 (m, 2H), 7.10 (bs, 4H). ^{13}C NMR (75 MHz, $CDCl_3$): δ 16.7, 21.3, 22.2, 24.6, 24.8, 42.8, 51.3, 126.0, 129.5, 134.0, 145.0, 158.9. HRMS (ESI) *m/z* calcd for $C_{14}H_{21}N_2$: 217.1699. Found: 217.1703.

1-Phenyl-2-iminopiperidine (**4b**)

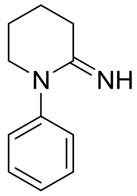

Yellow oil (79% yield). ^{1}H NMR (500 MHz, $CDCl_3$): δ 1.81-1.90 (m, 4H), 2.56-2.59 (m, 2H), 3.46 (t, *J*= 6.0, 2H), 5.29 (bs, ex, 1H), 7.19 (dd, *J*= 8.5, 1.4 Hz, 2H), 7.20-7.24 (m, 1H), 7.36-7.40 (m, 2H). ^{13}C NMR (125 MHz, $CDCl_3$): δ 22.1, 24.4, 32.5, 51.7, 127.0, 127.4, 130.0, 144.4, 164.4. HRMS (ESI) *m/z* calcd for $C_{11}H_{15}N_2$: 175.1230. Found: 175.1237.

1-(*p*-Chlorophenyl)-2-iminopiperidine (**4c**)

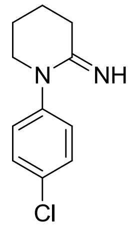

Yellow oil (72% yield). ^{1}H NMR (500 MHz, $CDCl_3$): δ 1.83-1.92 (m, 4H), 2.60 (t, *J*= 6.4 Hz, 2H), 3.46 (t, *J*= 6.0 Hz, 2H), 4.21 (bs, ex, 1H), 7.16 (d, *J*= 8.9 Hz, 2H), 7.37 (d, *J*= 8.9 Hz, 2H). ^{13}C NMR (125 MHz, $CDCl_3$): δ 22.1, 24.3, 32.9, 51.7, 128.5, 130.1, 132.4, 143.2, 164.7. HRMS (ESI) *m/z* calcd for $C_{11}H_{14}ClN_2$: 209.0840. Found: 209.0836.

1-(*o*-Tolyl)-2-iminopiperidine (**4d**)

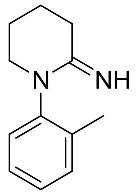

Yellow oil (74% yield). ^{1}H NMR (500 MHz, $CDCl_3$): δ 1.80-1.95 (m, 2H), 2.17 (s, 3H), 2.58-2.65 (m, 2H), 3.28-3.32 (m, 1H), 3.44-3.49 (m, 1H), 4.89 (bs, ex, 1H), 7.13 (dd, *J*= 7.6, 1.6 Hz, 1H), 7.19-7.28 (m, 3H). ^{13}C NMR (125 MHz, $CDCl_3$): δ 17.6, 22.0, 24.4, 32.0, 51.1, 127.9, 128.1, 128.2, 131.7, 136.6, 142.2, 163.1. HRMS (ESI) *m/z* calcd for $C_{12}H_{17}N_2$: 189.1386. Found: 189.1381.

1-(*o*-Fluorophenyl)-2-iminopiperidine (**4e**)

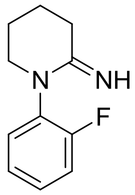

Yellow oil (82% yield). ^{1}H NMR (500 MHz, $CDCl_3$): δ 1.83-1.95 (m, 4H), 2.61 (t, *J*= 6.4 Hz, 2H), 3.44-3.46 (m, 2H), 4.82 (bs, ex, 1H), 7.12-7.19 (m, 2H), 7.23-7.29 (m, 2H). ^{13}C NMR (125 MHz, $CDCl_3$): δ 21.9, 24.2, 32.5, 51.2, 117.4 (d, *J*= 20.5 Hz), 125.4 (d, *J*= 3.9 Hz), 129.1 (d, *J*= 7.8 Hz), 129.9 (d, *J*= 2.0 Hz), 131.7 (d, *J*= 12.7 Hz), 158.7 (d, *J*= 251.4 Hz), 164.1. HRMS (ESI) *m/z* calcd for $C_{11}H_{14}FN_2$: 193.1136. Found: 193.1143.

1-(*o*-Chlorophenyl)-2-iminopiperidine (**4f**)

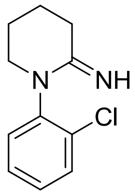

Yellow oil (73% yield). ^{1}H NMR (500 MHz, $CDCl_3$): δ 1.83-1.95 (m, 4H), 2.61-2.65 (m, 2H), 3.36-3.48 (m, 2H), 3.80 (bs, ex, 1H), 7.24-7.29 (m, 2H), 7.30-7.34 (m, 1H), 7.47-7.49 (m, 1H). ^{13}C NMR (125 MHz, $CDCl_3$): δ 21.7, 24.1, 32.2, 50.8, 128.7, 129.2, 130.5, 131.2, 133.5, 141.2, 163.5. HRMS (ESI) *m/z* calcd for $C_{11}H_{14}ClN_2$: 209.0840. Found: 209.0835.

1-(2,6-dimethylphenyl)-2-iminopiperidine (**4g**)

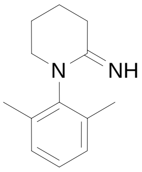

Yellow solid (86% yield), mp: 56-57°C. ^{1}H NMR (600 MHz, $CDCl_3$): δ 1.82-1.94 (m, 4H), 2.16 (s, 6H), 2.67 (t, *J*= 6.3 Hz, 2H), 3.29 (t, *J*= 6.0 Hz, 2H), 4.27 (bs, ex, 1H), 7.08-7.16(m, 3H). ^{13}C NMR (150 MHz, $CDCl_3$): δ 17.8, 21.7, 24.3, 31.5, 49.0, 128.2, 129.4, 136.9, 139.9, 161.9. HRMS (ESI) *m/z* calcd for $C_{13}H_{19}N_2$: 203.1543. Found: 203.1547.

1-(*p*-Tolyl)-2-iminoazepane (**7a**)

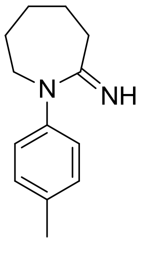

Brown oil (73% yield). ^{1}H NMR (500 MHz, $CDCl_3$): δ 1.72-1.78 (m, 4H), 1.82-1.85 (m, 2H), 2.35 (s, 3H), 2.67-2.69 (m, 2H), 3.62-3.64 (m, 2H), 7.07 (d, *J*= 8.1 Hz, 2H), 7.21 (d, *J*= 8.1 Hz, 2H). ^{13}C NMR (125 MHz, $CDCl_3$): δ 21.2, 25.9, 29.6, 29.9, 36.7, 54.0, 127.6, 130.7, 136.9, 142.7, 169.7. HRMS (ESI) *m/z* calcd for $C_{13}H_{19}N_2$: 203.1543. Found: 203.1549.

1-(*p*-Tolyl)-2-ethyliminoazepane (**8a**)

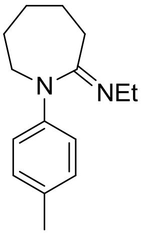

This compound was obtained as a mixture of E and Z isomers (55% E, 45% Z). Yellow oil (77% yield). ^{1}H NMR (600 MHz, $CDCl_3$): δ 1.00 (t, *J*= 7.2 Hz, 3H, E), 1.01 (t, *J*= 7.2 Hz, 3H, Z), 1.55-1.58 (m, 2H, Z), 1.65-1.77 (m, 10H, Z/E), 2.29 (s, 3H, Z), 2.30 (s, 3H, E), 2.59-2.61 (m, 2H, E), 2.66 (c, 2H, *J*= 7.2 Hz, Z), 2.74-2.76 (m, 2H, Z), 3.24 (c, *J*= 7.2 Hz, 2H, E), 3.63-3.65 (m, 2H, Z), 3.66-3.68 (m, 2H, E), 6.84 (d, *J*= 8.1 Hz, 2H, Z), 7.06-7.08 (m, 4H, Z/E), 7.11 (d, *J*= 8.1 Hz, 2H, E). ^{13}C NMR (150 MHz, $CDCl_3$): δ 17.0 (Z), 17.4 (E), 21.0 (Z), 21.3 (E), 25.38, 25.44, 25.6, 26.7 (Z), 29.4 (E), 30.0, 30.1, 39.5 (Z), 43.9 (E), 45.5 (Z), 52.6 (Z), 53.0 (E), 122.8 (Z), 127.4 (E), 129.7 (E), 130.0 (Z), 132.0 (Z), 134.2 (E), 143.7 (Z), 145.5 (E), 160.9 (Z), 163.4 (E). HRMS (ESI) *m/z* calcd for $C_{15}H_{23}N_2$: 231.1856. Found: 231.1859.

1-Phenyl-2-iminoazepane (**7b**)

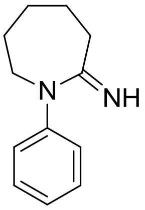

Brown oil (62% yield). ^{1}H NMR (500 MHz, $CDCl_3$): δ 1.71-1.78 (m, 4H), 1.82-1.86 (m, 2H), 2.67-2.69 (m, 2H), 3.64-3.66 (m, 2H), 7.18 (dd, *J*= 8.4, 1.3 Hz, 2H), 7.24-7.27 (m, 1H), 7.38-7.42 (m, 2H). ^{13}C NMR (125 MHz, $CDCl_3$): δ 26.1, 29.8, 30.0, 37.1, 54.1, 127.2, 128.0, 130.2, 145.5, 169.7. HRMS (ESI) *m/z* calcd for $C_{12}H_{17}N_2$: 189.1386. Found: 189.1390.

1-(*p*-Fluorophenyl)-2-iminoazepane (**7c**)

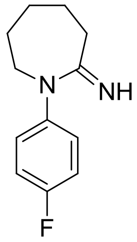

Brown oil (62% yield). ^{1}H NMR (500 MHz, $CDCl_3$): δ 1.66-1.73 (m, 4H), 1.77-1.81 (m, 2H), 2.63-2.65 (m, 2H), 3.57-3.59 (m, 2H), 4.71 (bs, ex, 1H), 7.02-7.06 (m, 2H), 7.12 (dd, *J*= 9.2, 5.0 Hz, 2H). ^{13}C NMR (125 MHz, $CDCl_3$): δ 25.9, 29.6, 29.8, 36.9, 54.2, 116.9 (d, *J*= 22.7 Hz), 129.6 (d, J=8.2), 141.3 (d, J= 3.6), 161.3 (d, *J*= 247.1 Hz), 169.9. HRMS (ESI) *m/z* calcd for $C_{12}H_{16}FN_2$: 207.1292. Found: 207.1297.

1-(*p*-Bromophenyl)-2-iminoazepane (**7d**)

Brown oil (75% yield). ^{1}H NMR (500 MHz, $CDCl_3$): δ 1.68-1.74 (m, 4H),

1.78-1.82 (m, 2H), 2.64-2.66 (m, 2H), 3.59-3.61 (m, 2H), 4.33 (bs, ex, 1H), 7.05 (d, *J*= 8.7 Hz, 2H), 7.49 (d, *J*= 8.7 Hz, 2H). ^{13}C NMR (125 MHz, $CDCl_3$): δ 25.9, 29.6, 29.8, 37.2, 54.0, 120.5, 129.6, 133.2, 144.5, 169.7. HRMS (ESI) *m/z* calcd for $C_{12}H_{16}BrN_2$: 267.0491. Found: 267.0486.

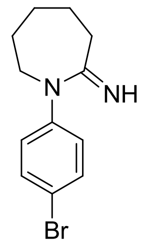

1-(*o*-Tolyl)-2-iminoazepane (**7e**)

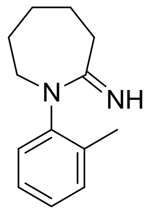

Brown oil (55% yield). ^{1}H NMR (500 MHz, $CDCl_3$): δ 1.66-1.96 (m, 6H), 2.22 (s, 3H), 2.67-2.75 (m, 2H), 3.44-3.48 (m, 1H), 3.65-3.70 (m, 1H), 4.90 (bs, ex, 1H), 7.10 (dd, *J*= 7.3, 1.6 Hz, 1H), 7.19-7.29 (m, 3H). ^{13}C NMR (125 MHz, $CDCl_3$): δ 18.0, 25.8, 29.8, 30.1, 36.6, 53.5, 127.89, 127.91, 128.5, 131.7, 136.3, 143.7, 169.0. HRMS (ESI) *m/z* calcd for $C_{13}H_{19}N_2$: 203.1543. Found: 203.1545.

1-(*o*-Fluorophenyl)-2-iminoazepane (**7f**)

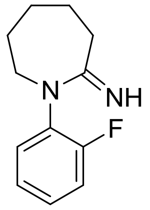

Brown oil (68% yield). ^{1}H NMR (500 MHz, $CDCl_3$): δ 1.75-1.77 (m, 4H), 1.85-1.86 (m, 2H), 2.70-2.72 (m, 2H), 3.61 (bs, 2H), 7.14-7.18 (m, 2H), 7.23-7.25 (m, 1H), 7.25-7.29 (m, 1H). ^{13}C NMR (125 MHz, $CDCl_3$): δ 26.0, 29.6, 30.0, 37.5, 53.9, 117.3 (d, *J*= 20.5 Hz), 125.3 (d, *J*= 3.9 Hz), 128.9 (d, *J*= 7.8 Hz), 130.4 (d, *J*= 2.2 Hz), 132.8 (d, *J*= 12.7 Hz), 158.5 (d, *J*= 250.4 Hz), 169.5. HRMS (ESI) *m/z* calcd for $C_{12}H_{16}FN_2$: 207.1292. Found: 207.1298.

1-(2,6-dimethylphenyl)-2-iminoazepane (**7g**)

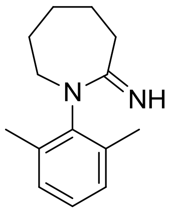

Brown oil (28% yield). ^{1}H NMR (500 MHz, $CDCl_3$): δ 1.74-1.82 (m, 4H), 1.84-1.88 (m, 2H), 2.20 (s, 6H), 2.69-2.71 (m, 2H), 3.49-3.51 (m, 2H), 7.09 (bs, 3H). ^{13}C NMR (125 MHz, $CDCl_3$): δ 18.7, 25.7, 30.3, 30.5, 37.0, 53.2, 127.8, 129.4, 136.7, 142.5, 168.1. HRMS (ESI) *m/z* calcd for $C_{14}H_{21}N_2$: 217.1699. Found: 217.1706.

4-(*o*-Chlorophenylamino)butyronitrile (**1g**)

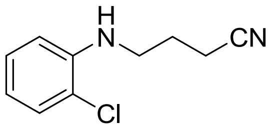

White oil (49% yield). ^{1}H NMR (600 MHz, $CDCl_3$): δ 1.98-2.07 (m, 2H), 2.51 (t, *J*= 7.0 Hz, 2H), 3.39 (t, *J*= 6.6 Hz, 2H), 4.38 (bs, ex, 1H), 6.67-6.72 (m, 2H), 7.16-7.21 (m, 1H), 7.30 (dd, *J*= 8.0, 1.4 Hz, 1H). ^{13}C NMR (150 MHz, $CDCl_3$): δ 15.1, 25.3, 42.2, 111.4, 118.1, 119.6, 119.5, 128.2, 129.6, 143.6. HRMS (ESI) *m/z* calcd for $C_{10}H_{12}ClN_2$: 195.0684. Found: 195.0678.

4-(*o*-Methoxyphenylamino)butyronitrile (**1h**)

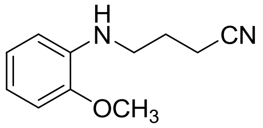

White oil (77% yield). ^{1}H NMR (600 MHz, $CDCl_3$): δ 1.96-2.01 (m, 2H), 2.47 (t, *J*= 7.1 Hz, 2H), 3.32 (t, *J*= 6.6 Hz, 2H), 3.86 (s, 3H), 4.28 (bs, ex, 1H), 6.63 (dd, *J*= 7.9, 1.5 Hz, 1H), 6.70-6.74 (m, 1H), 6.81 (dd, *J*= 8.0, 1.4 Hz, 1H), 6.89-6.92 (m, 1H). ^{13}C NMR (150 MHz, $CDCl_3$): δ 15.0, 25.5, 42.2, 55.6, 109.8, 110.0, 117.2, 119.7, 121.5, 127.7, 147.1. HRMS (ESI) *m/z* calcd for $C_{11}H_{15}N_2O$: 191.1179. Found: 191.1184.

5-(*o*-Fluorophenylamino)valeronitrile (**3e**)

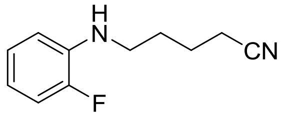

Yellow oil (70% yield). ^{1}H NMR (500 MHz, $CDCl_3$): δ 1.76-1.85 (m, 4H), 2.38-2.42 (m, 2H), 3.20-3.24 (m, 2H), 3.96 (bs, ex, 1H), 6.62-6.67 (m, 1H), 6.68-6.72 (m, 1H), 6.95-7.02 (m, 2H). ^{13}C NMR (125 MHz, $CDCl_3$): δ 17.3, 23.3, 28.8, 43.1, 112.4 (d, *J*= 3.9 Hz), 114.8 (d, *J*= 18.6 Hz), 117.2 (d, *J*= 6.9 Hz), 119.7, 125.9 (d, *J*= 2.9 Hz), 136.6 (d, J= 11.7 Hz), 151.9 (d, *J*= 237.7 Hz). HRMS (ESI) *m/z* calcd for $C_{11}H_{14}FN_2$: 193.1136. Found: 193.1140.

5-(*o*-Chlorophenylamino)valeronitrile (**3f**)

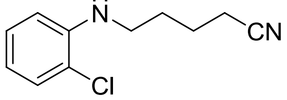

Yellow oil (61% yield). ^{1}H NMR (500 MHz, $CDCl_3$): δ 1.77-1.86 (m, 4H), 2.41 (t, *J*= 6.8 Hz, 2H), 3.24 (t, *J*= 6.5 Hz, 2H), 4.40 (bs, ex, 1H), 6.63-6.67 (m, 2H), 7.13-7.17 (m, 1H), 7.25-7.27 (m, 1H). ^{13}C NMR (125 MHz, $CDCl_3$): δ 17.4, 23.3, 28.6, 43.1, 111.5, 117.8, 119.5, 119.7, 128.2, 129.5, 143.9. HRMS (ESI) *m/z* calcd for $C_{11}H_{14}ClN_2$: 209.0840.

Found: 209.0845.

5-(2,6-Dimethylphenylamino)valeronitrile (**3g**)

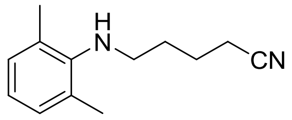

Yellow oil (45% yield). ^{1}H NMR (500 MHz, $CDCl_3$): δ 1.77-1.83 (m, 4H), 2.29 (s, 6H), 2.41 (t, *J*= 6.9 Hz, 2H), 2.93 (bs, ex, 1H), 3.01 (t, *J*= 6.8 Hz, 2H), 6.82-6.85 (m, 1H), 7.01 (d, *J*= 7.3 Hz, 2H). ^{13}C NMR (125 MHz, $CDCl_3$): δ 17.4, 18.7, 23.5, 30.4, 47.8, 119.8, 122.4, 129.1, 129.8, 146.0. HRMS (ESI) *m/z* calcd for $C_{13}H_{19}N_2$: 203.1543. Found: 203.1539.

6-(*p*-Tolylamino)hexanenitrile (**6a**)

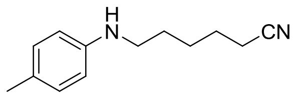

White solid (66% yield), mp: 77-78°C. ^{1}H NMR (500 MHz, $CDCl_3$): δ 1.53-1.59 (m, 2H), 1.63-1.74 (m, 4H), 2.24 (s, 3H), 2.36 (t, *J*= 7.1 Hz, 2H), 3.12 (t, *J*= 6.9 Hz, 2H), 6.56 (d, *J*= 8.3 Hz, 2H), 6.99-7.01 (m, 2H). ^{13}C NMR (125 MHz, $CDCl_3$): δ 17.5, 20.7, 25.5, 26.6, 29.1, 44.4, 113.5, 119.9, 127.2, 130.1, 146.0. HRMS (ESI) *m/z* calcd for $C_{13}H_{19}N_2$: 203.1543. Found: 203.1545.

6-Phenylaminohexanenitrile (**6b**)

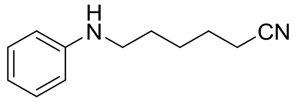

Yellow oil (57% yield). ^{1}H NMR (500 MHz, $CDCl_3$): δ 1.54-1.60 (m, 2H), 1.64-1.74 (m, 4H), 2.36 (t, *J*= 7.1 Hz, 2H), 3.15 (t, *J*= 6.9 Hz, 2H), 3.59 (bs, ex, 1H), 6.62 (dd, *J*= 8.5, 1.2 Hz, 2H), 6.71 (tt, *J*= 7.3, 1.2, 1H), 7.17-7.21 (m, 2H). ^{13}C NMR (125 MHz, $CDCl_3$): δ 17.5, 25.5, 26.6, 29.1, 43.9, 113.1, 117.7, 119.9, 129.6, 148.4. HRMS (ESI) *m/z* calcd for $C_{12}H_{17}N_2$: 189.1386. Found: 189.1392.

6-(*p*-Fluorophenylamino)hexanenitrile (**6c**)

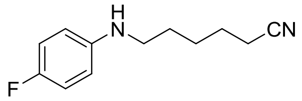

Yellow oil (55% yield). ^{1}H NMR (600 MHz, $CDCl_3$): δ 1.50-1.73 (m, 6H), 2.34 (t, *J*= 6.9 Hz, 2H), 3.07 (t, *J*= 6.7 Hz, 2H), 3.38 (bs, ex, 1H), 6.52 (dd, *J*= 9.0, 4.4 Hz, 2H), 6.83-6.92 (m, 2H). ^{13}C NMR (150 MHz, $CDCl_3$): δ 17.3, 25.4, 26.4, 28.9, 44.4, 113.7 (d, *J*= 7.2 Hz), 115.8 (d,

J= 22.7 Hz), 119.9, 144.9 (d, *J*= 2.2 Hz), 155.9 (d, *J*= 234.4 Hz). HRMS (ESI) *m/z* calcd for $C_{12}H_{16}FN_2$: 207.1292. Found: 207.1296.

6-(*p*-Bromophenylamino)hexanenitrile (**6d**)

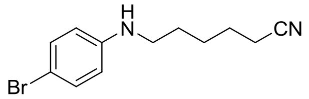

White solid (50% yield), mp: 100-102°C. ^{1}H NMR (500 MHz, $CDCl_3$): δ 1.52-1.59 (m, 2H), 1.60-1.65 (m, 2H), 1.67-1.73 (m, 2H), 2.36 (t, *J*= 7.0 Hz, 2H), 3.09 (t, *J*= 6.9 Hz, 2H), 3.67 (bs, ex, 1H), 6.46 (d, *J*= 8.9 Hz, 2H), 7.24 (d, *J*= 8.9 Hz, 2H). ^{13}C NMR (125 MHz, $CDCl_3$): δ 17.4, 25.4, 26.5, 28.9, 43.8, 109.0, 114.5, 119.9, 132.2, 147.4. HRMS (ESI) *m/z* calcd for $C_{12}H_{16}BrN_2$: 267.0491. Found: 267.0497.

6-(*o*-Tolylamino)hexanenitrile (**6e**)

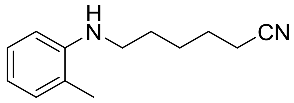

Yellow oil (68% yield). ^{1}H NMR (600 MHz, $CDCl_3$): δ 1.54-1.78 (m, 6H), 2.16 (s, 3H), 2.38 (t, *J*= 6.9 Hz, 2H), 3.20 (t, *J*= 6.9 Hz, 2H), 3.48 (bs, ex, 1H), 6.62-6.71 (m, 2H), 7.08 (d, *J*= 7.1 Hz, 1H), 7.15 (t, *J*= 7.7 Hz, 1H). ^{13}C NMR (150 MHz, $CDCl_3$): δ 17.4, 17.8, 25.5, 26.6, 29.0, 43.9, 110.1, 117.4, 119.9, 122.3, 127.4, 130.4, 146.1. HRMS (ESI) *m/z* calcd for $C_{13}H_{19}N_2$: 203.1543. Found: 203.1535.

6-(*o*-Fluorophenylamino)hexanenitrile (**6f**)

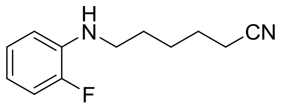

Brown oil (60% yield). ^{1}H NMR (500 MHz, $CDCl_3$): δ 1.55-1.61 (m, 2H), 1.67-1.75 (m, 4H), 2.37 (t, *J*= 7.0 Hz, 2H), 3.18 (t, *J*= 7.0 Hz, 2H), 4.07 (bs, ex, 1H), 6.61-6.66 (m, 1H), 6.68-6.72 (m, 1H), 6.95-7.02 (m, 2H). ^{13}C NMR (125 MHz, $CDCl_3$): δ 17.5, 25.5, 26.5, 29.0, 43.7, 112.5 (d, *J*= 3.3 Hz), 114.8 (d, *J*= 18.7 Hz), 117.1 (d, *J*= 6.6 Hz), 119.9, 124.9 (d, *J*= 3.3 Hz), 136.6 (d, *J*= 12.1 Hz), 151.9 (d, *J*= 237.7 Hz). HRMS (ESI) *m/z* calcd for $C_{12}H_{16}FN_2$: 207.1292. Found: 207.1288.

6-(2,6-Dimethylphenylamino)hexanenitrile (**6g**)

Yellow oil (52% yield). ^{1}H NMR (500 MHz, $CDCl_3$): δ 1.50-1.63 (m, 4H), 1.65-1.71 (m, 2H), 2.27 (s, 6H), 2.34 (t, *J*= 7.1 Hz, 2H), 2.60 (bs, ex,

1H), 2.97 (t, *J*= 7.0 Hz, 2H), 6.79-6.82 (m, 1H), 6.98 (d, *J*= 7.3 Hz, 2H). ^{13}C NMR (125 MHz, $CDCl_3$): δ 17.3, 18.7, 25.5, 26.5, 30.6, 48.3, 119.9, 122.0, 129.0, 129.5, 146.2. HRMS (ESI) *m/z* calcd for $C_{14}H_{21}N_2$: 217.1699. Found: 217.1701.

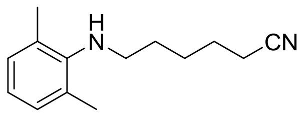

4. Copies of ^{1}H and ^{13}C NMR Spectra of compounds **1–8**

^{1}H NMR (500 MHz, $CDCl_3$) spectrum of compound **2a**

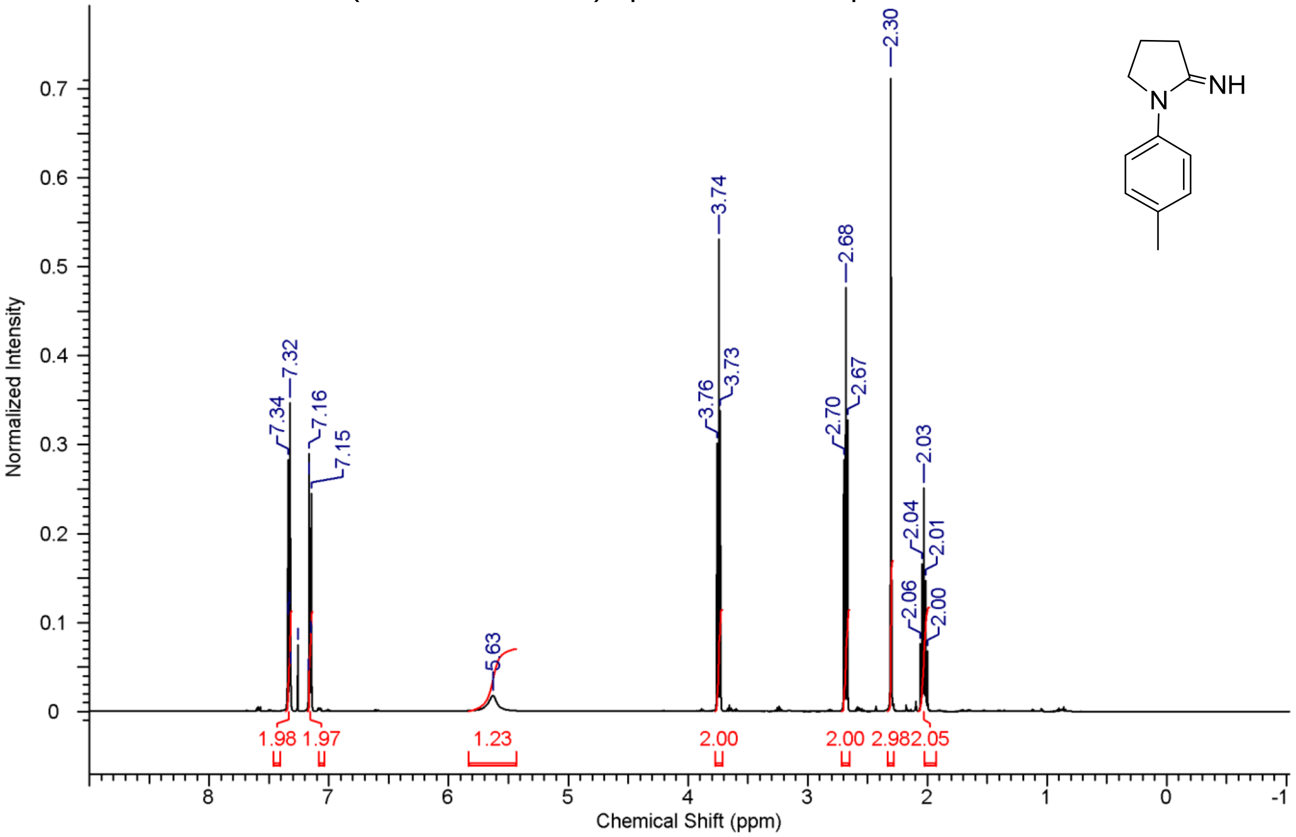

^{13}C NMR (125 MHz, $CDCl_3$) spectrum of compound **2a**

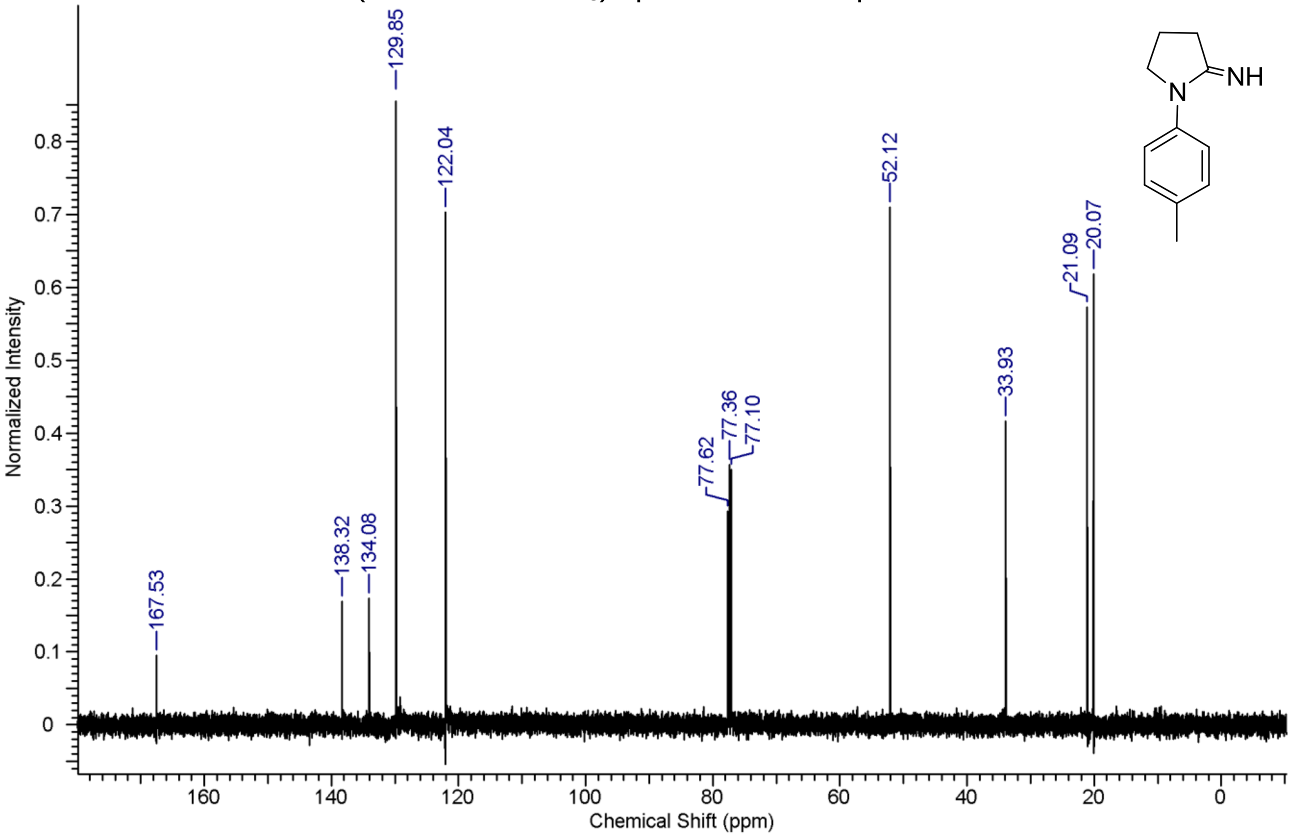

^{1}H NMR (500 MHz, $CDCl_3$) spectrum of compound **2b**

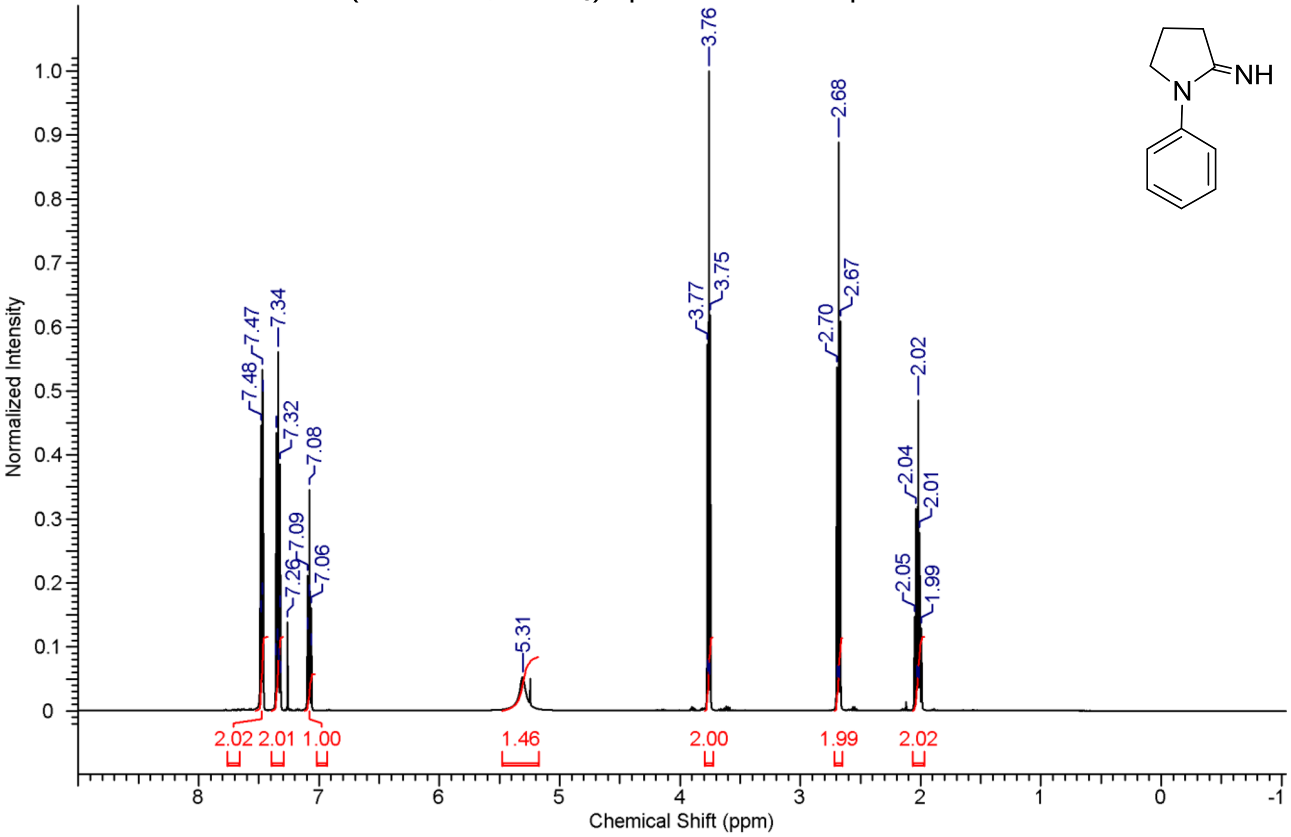

^{13}C NMR (125 MHz, $CDCl_3$) spectrum of compound **2b**

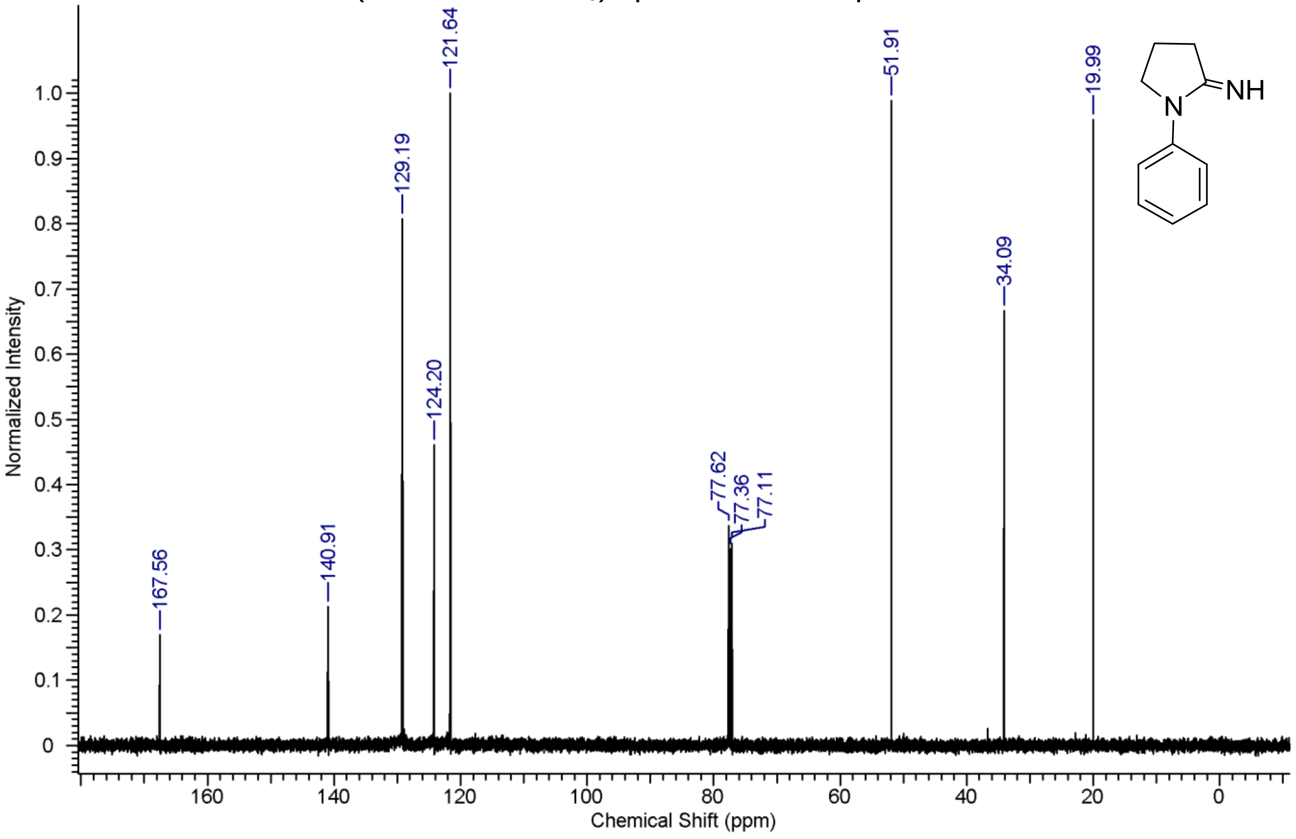

^{1}H NMR (600 MHz, $CDCl_3$) spectrum of compound **2c**

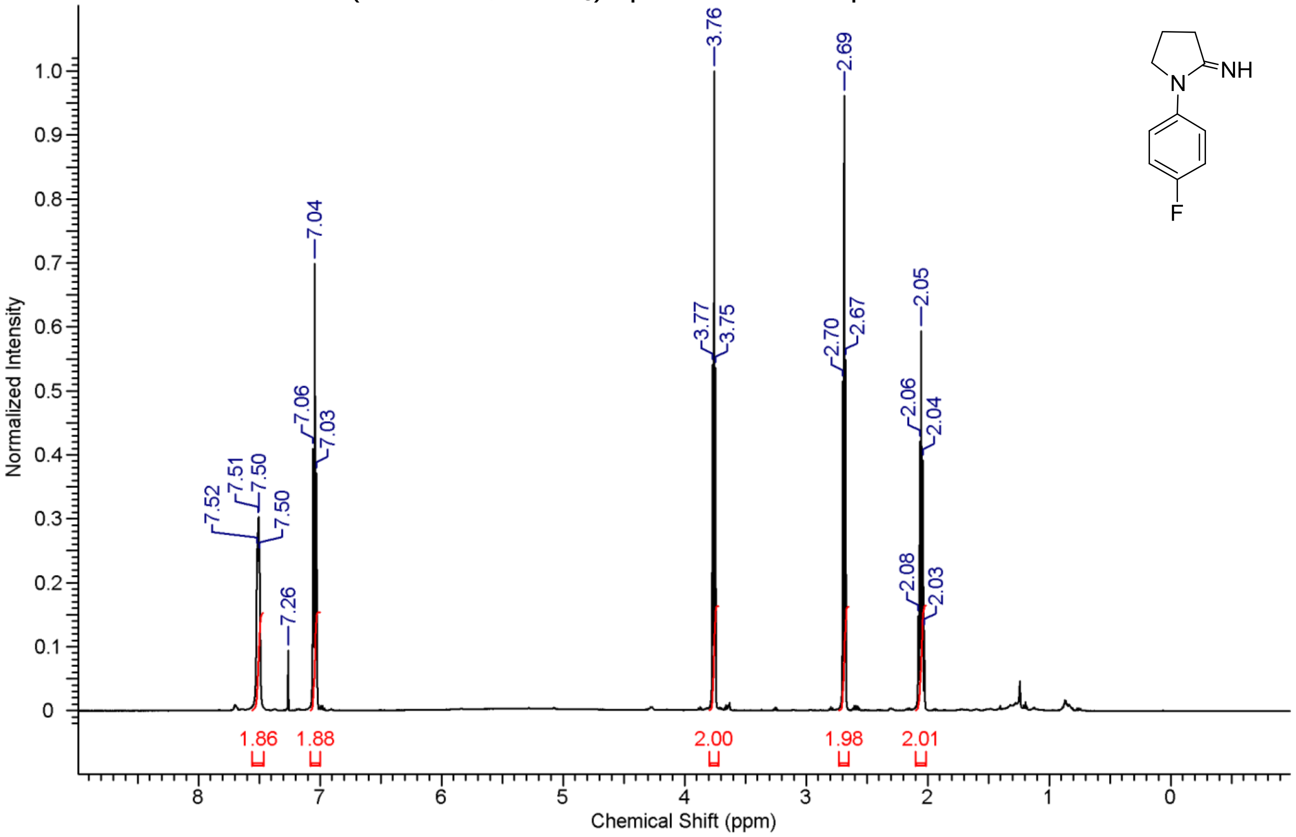

^{13}C NMR (150 MHz, $CDCl_3$) spectrum of compound **2c**

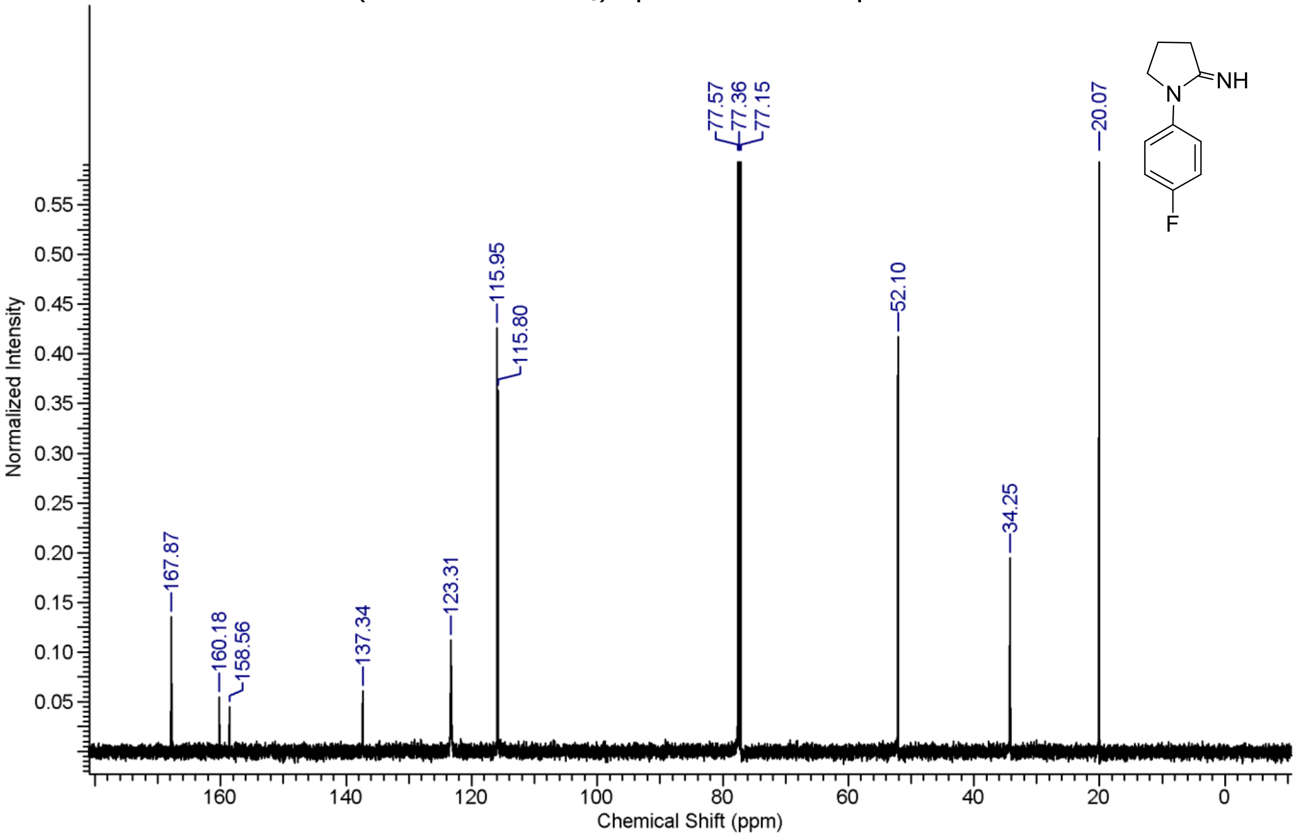

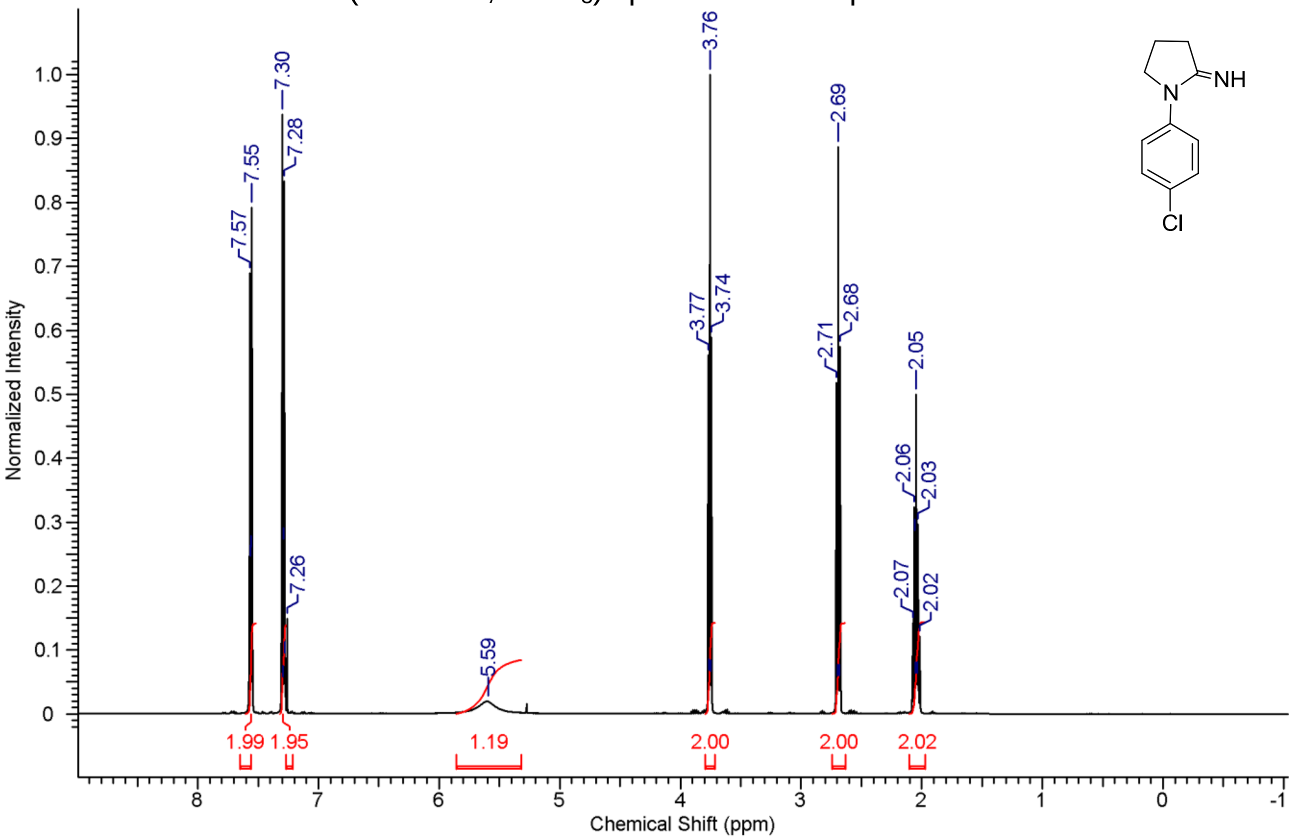

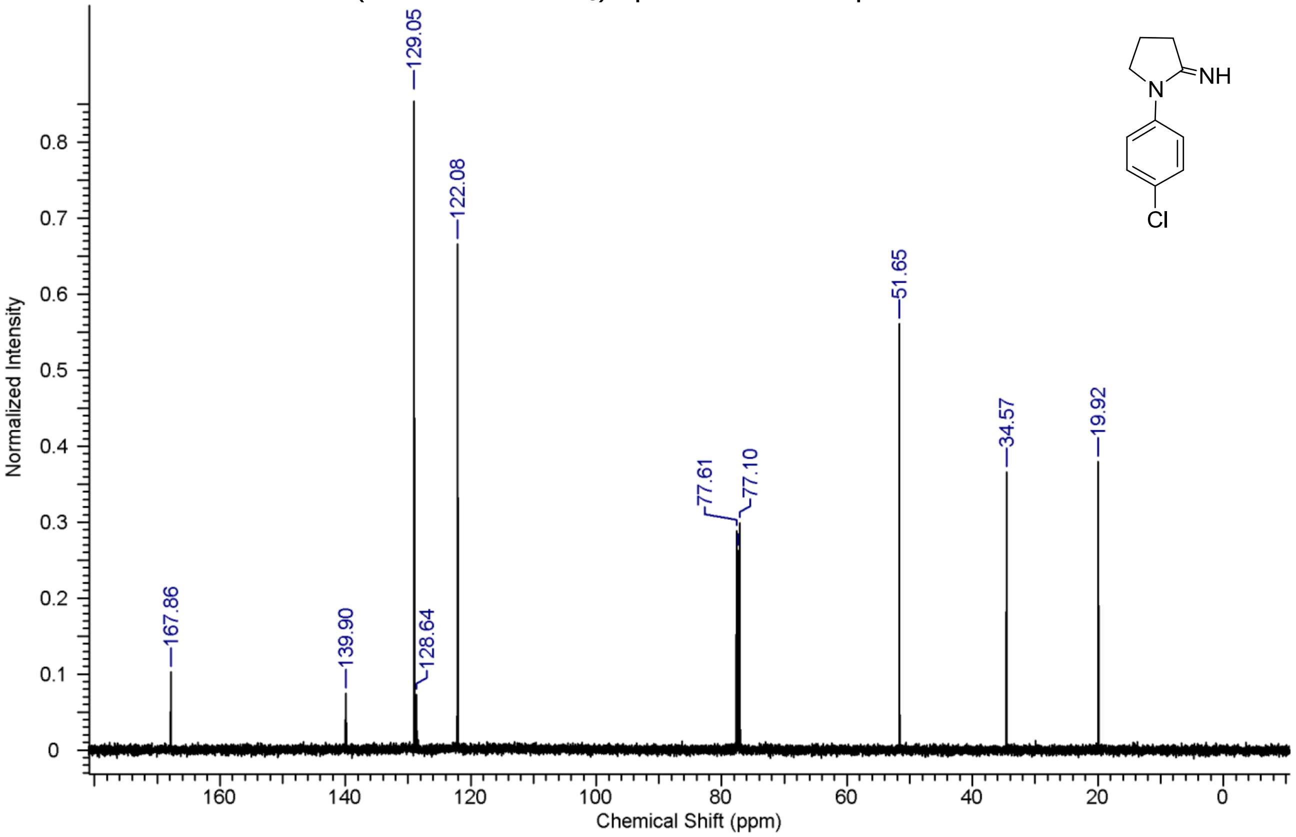

^{1}H NMR (600 MHz, $CDCl_3$) spectrum of compound **2e**

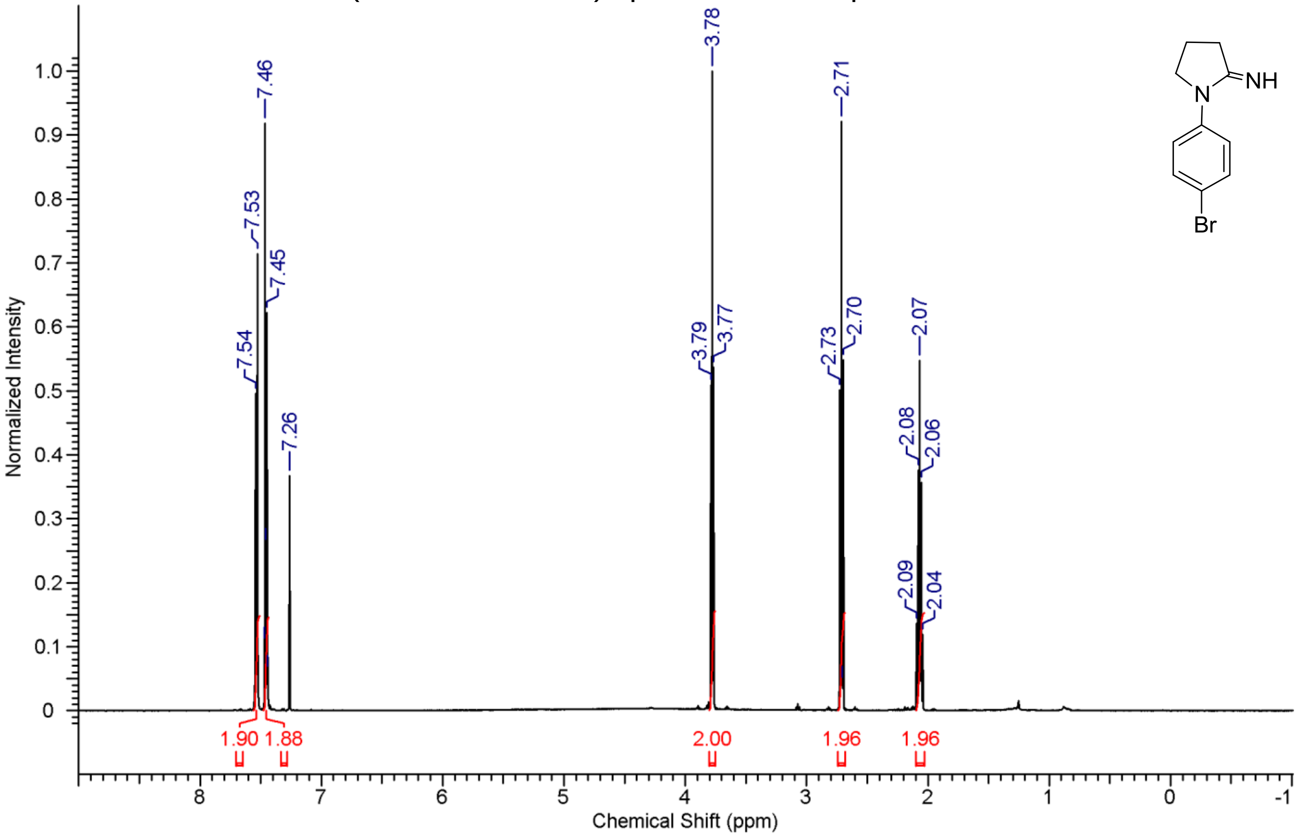

^{13}C NMR (125 MHz, $CDCl_3$) spectrum of compound **2e**

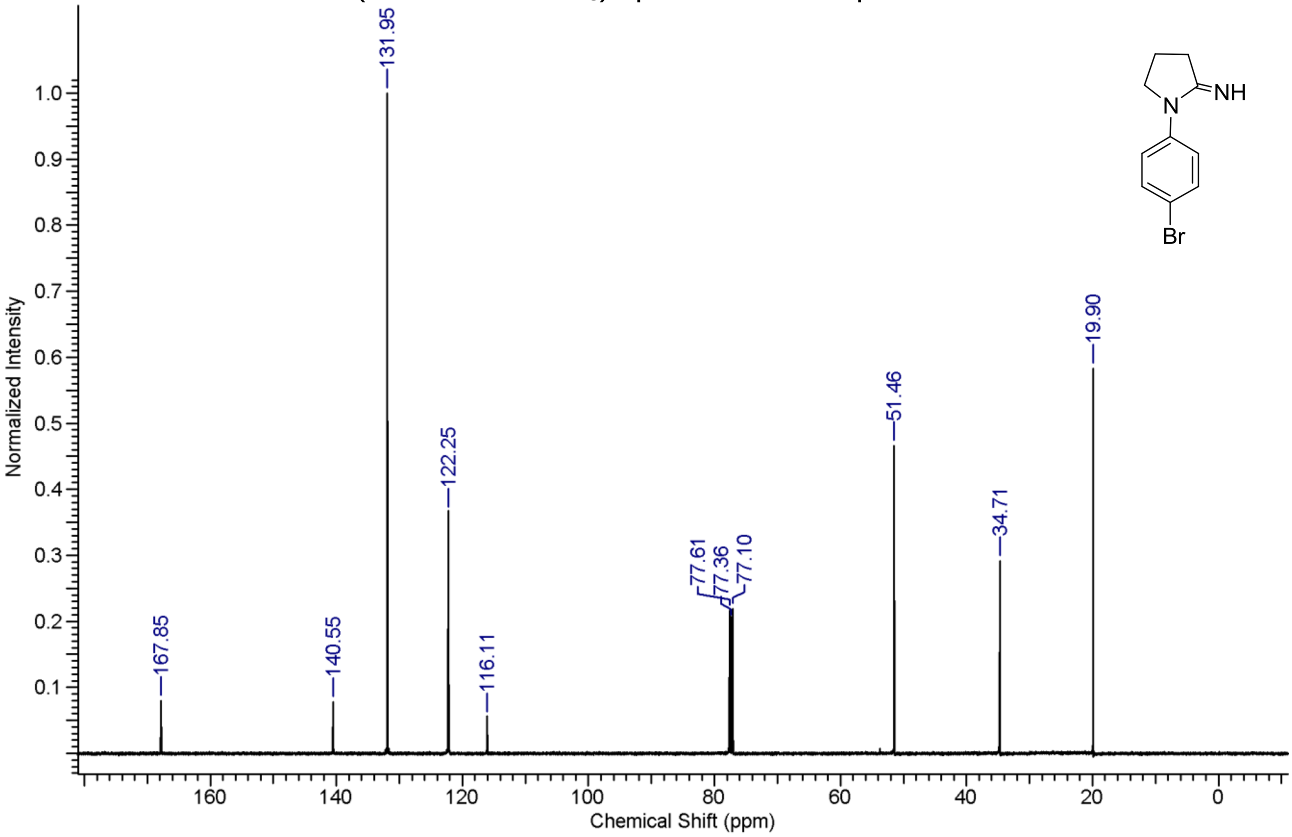

^{1}H NMR (500 MHz, $CDCl_3$) spectrum of compound **2f**

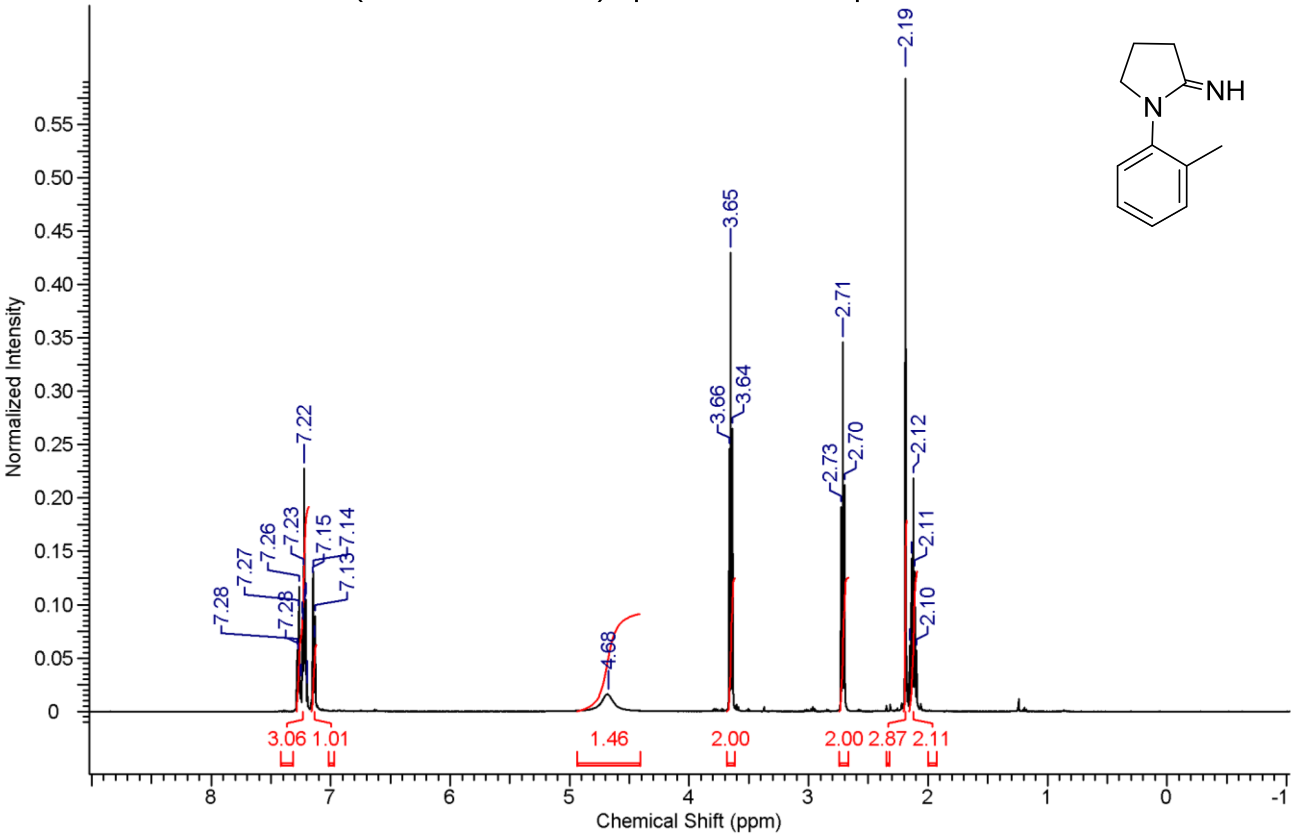

^{13}C NMR (125 MHz, $CDCl_3$) spectrum of compound **2f**

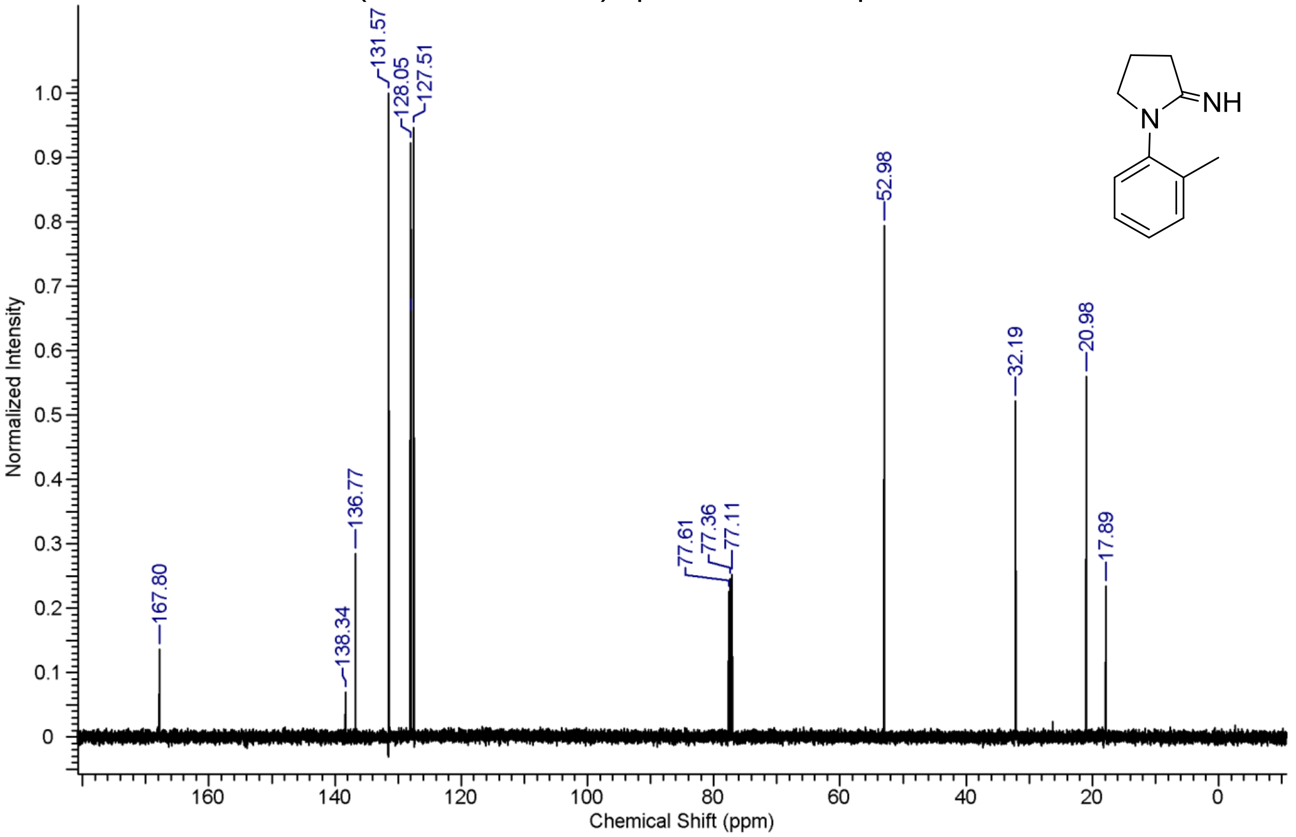

^{1}H NMR (500 MHz, $CDCl_3$) spectrum of compound **2g**

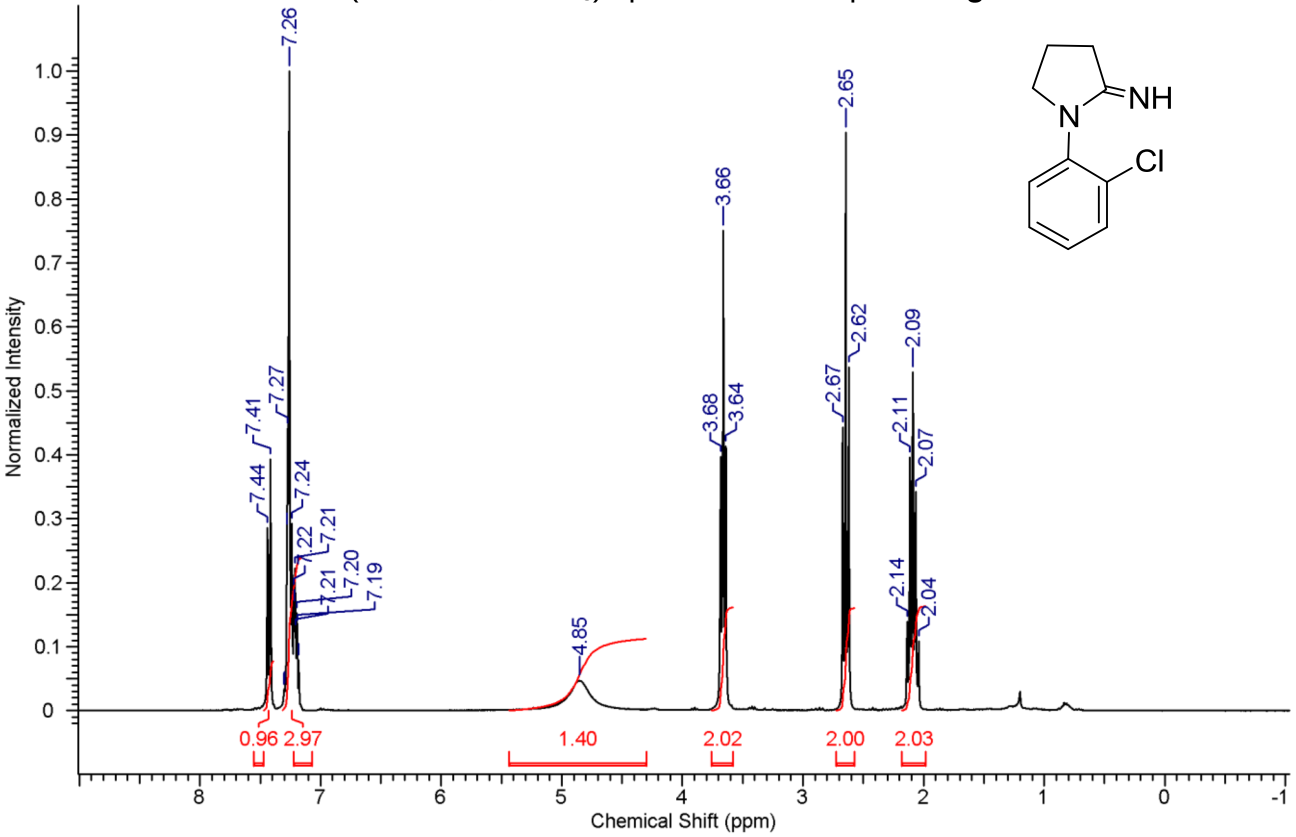

^{13}C NMR (125 MHz, $CDCl_3$) spectrum of compound **2g**

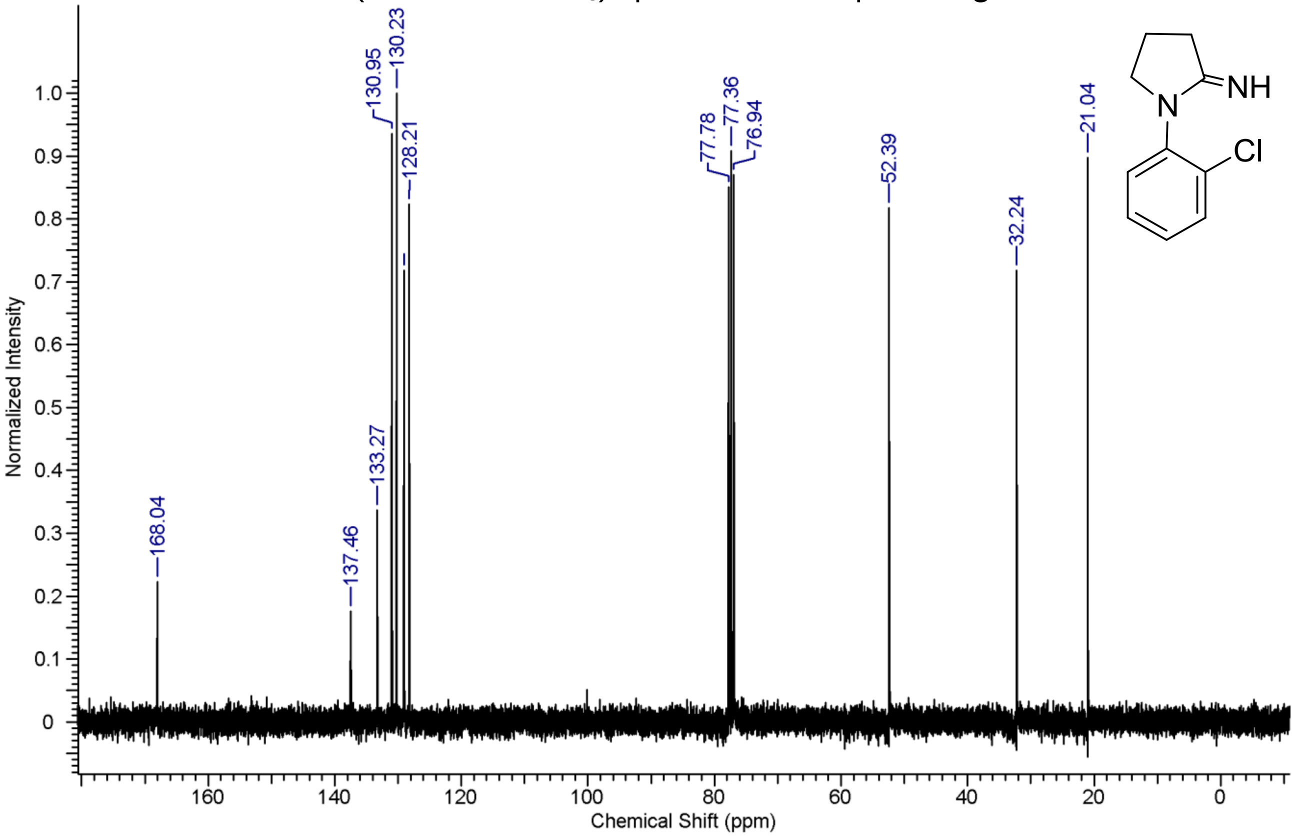

^{1}H NMR (600 MHz, $CDCl_3$) spectrum of compound **2h**

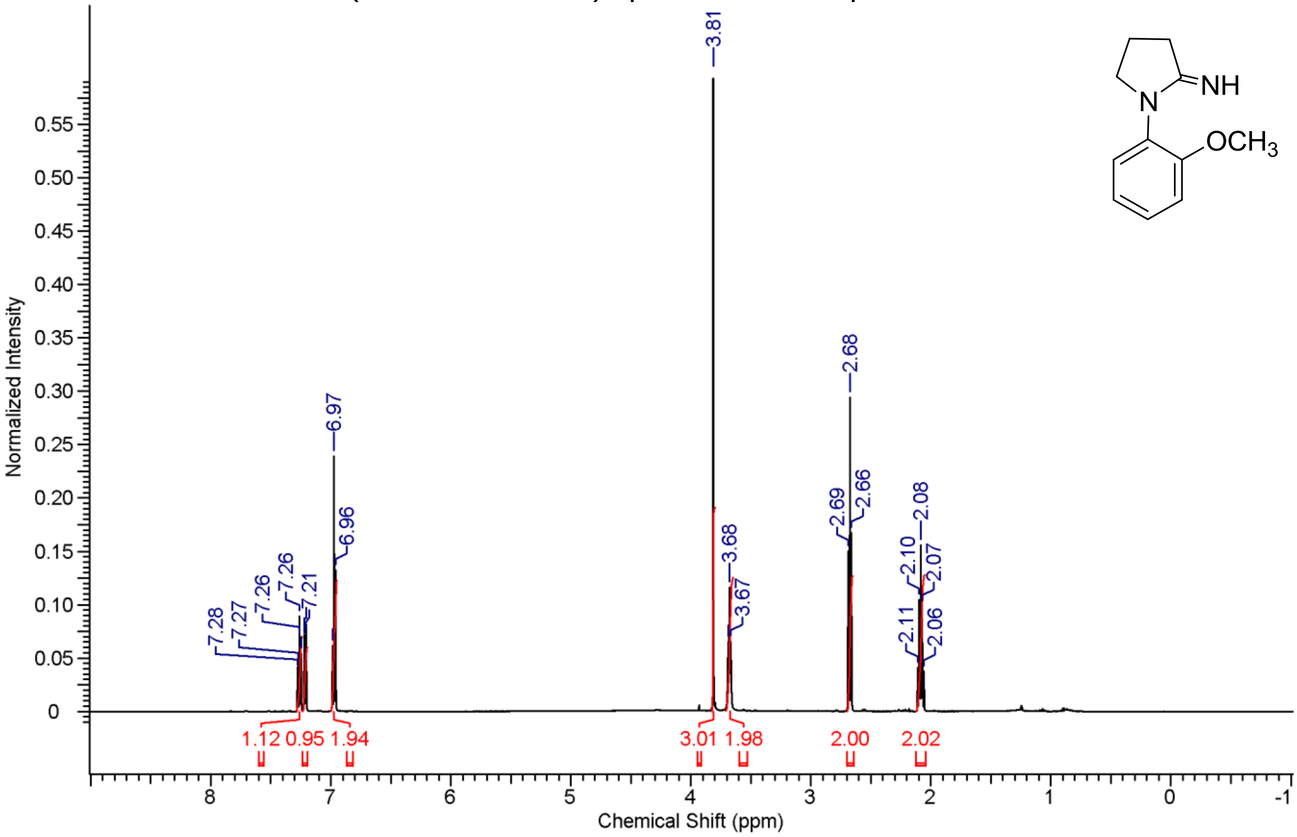

^{13}C NMR (150 MHz, $CDCl_3$) spectrum of compound **2h**

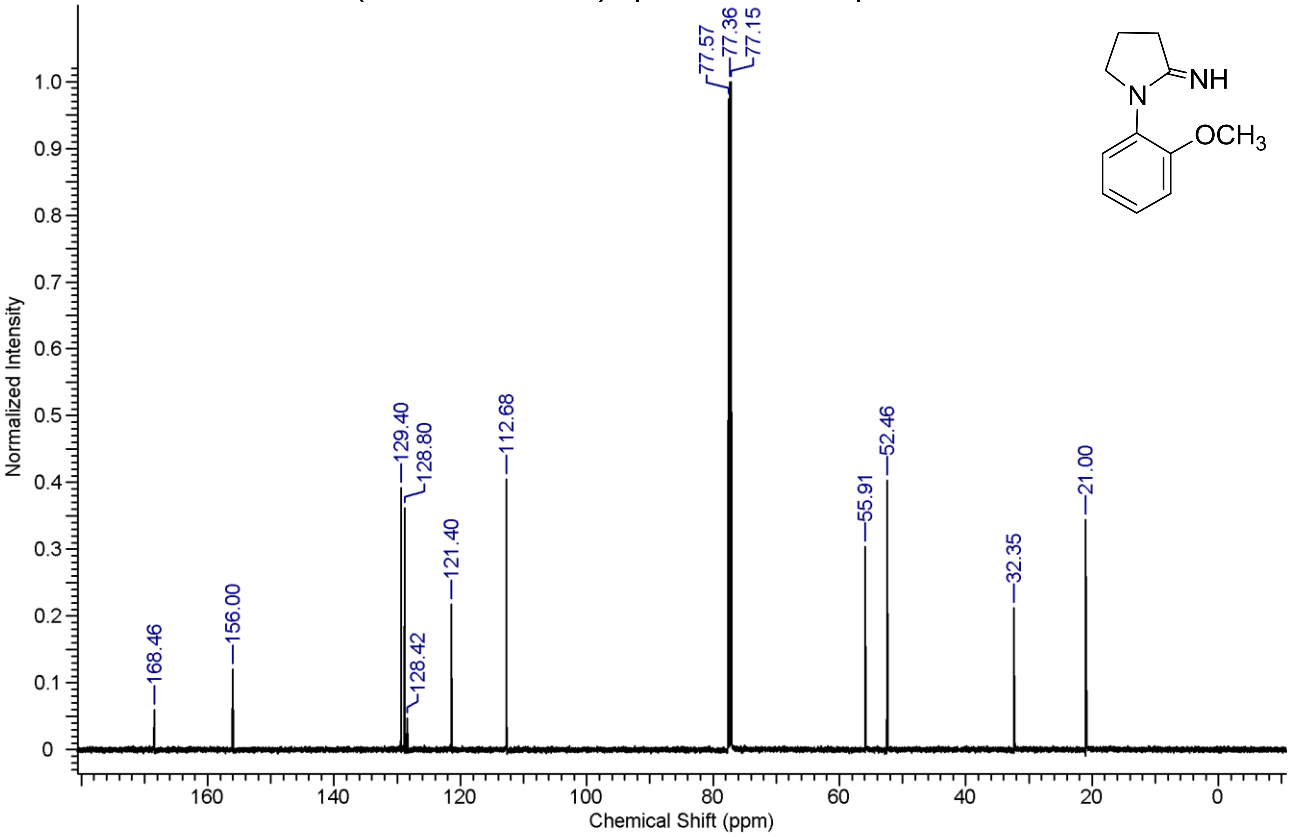

^1H NMR (500 MHz, $CDCl_3$) spectrum of compound **4a**

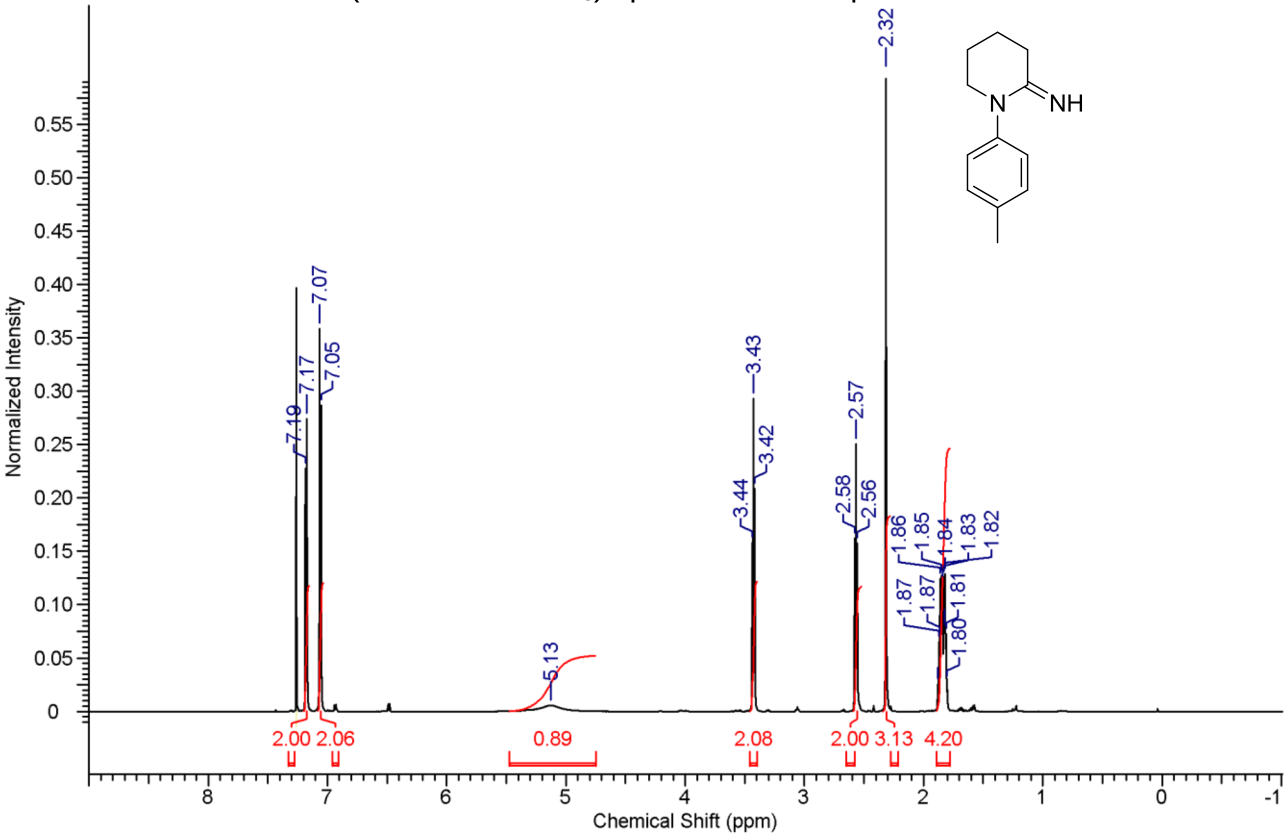

^{13}C NMR (125 MHz, $CDCl_3$) spectrum of compound **4a**

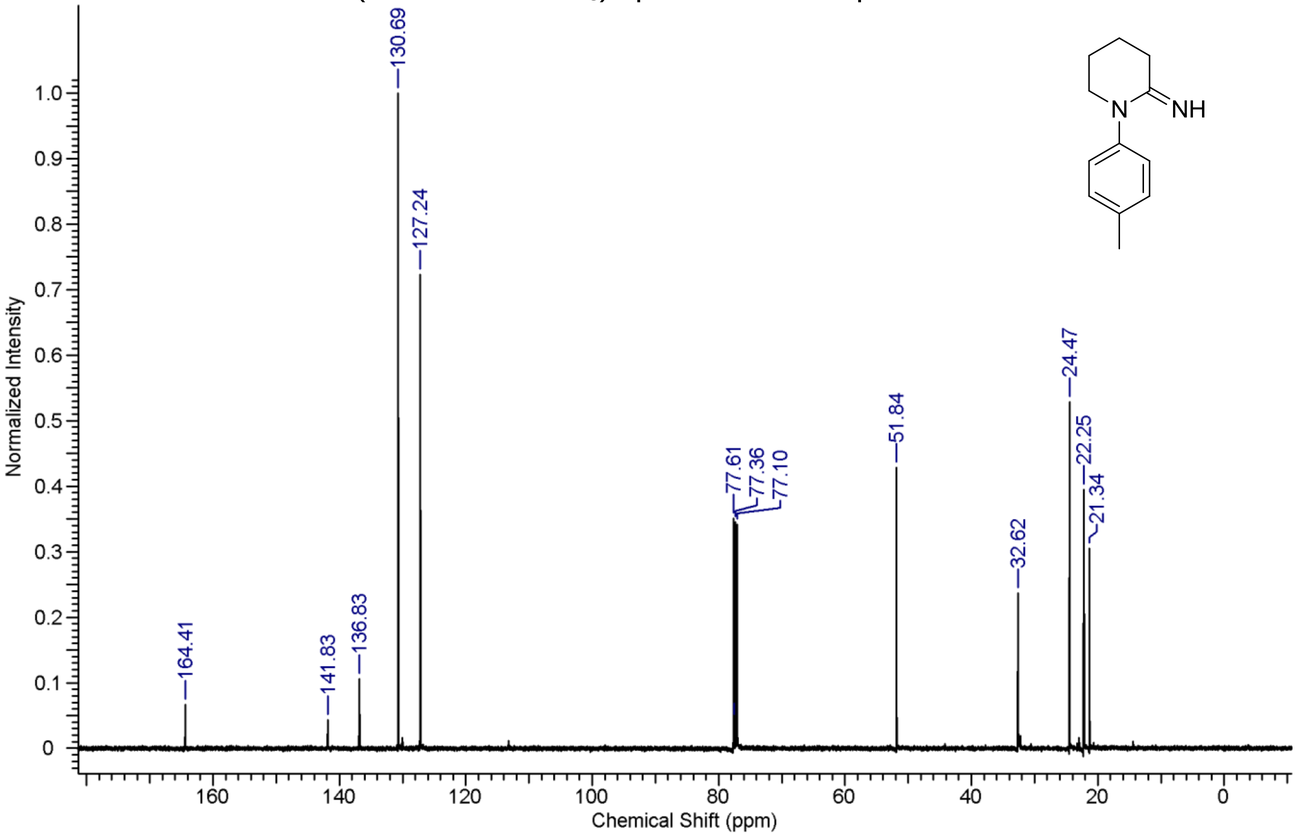

^{1}H NMR (300 MHz, $CDCl_3$) spectrum of compound **5a**

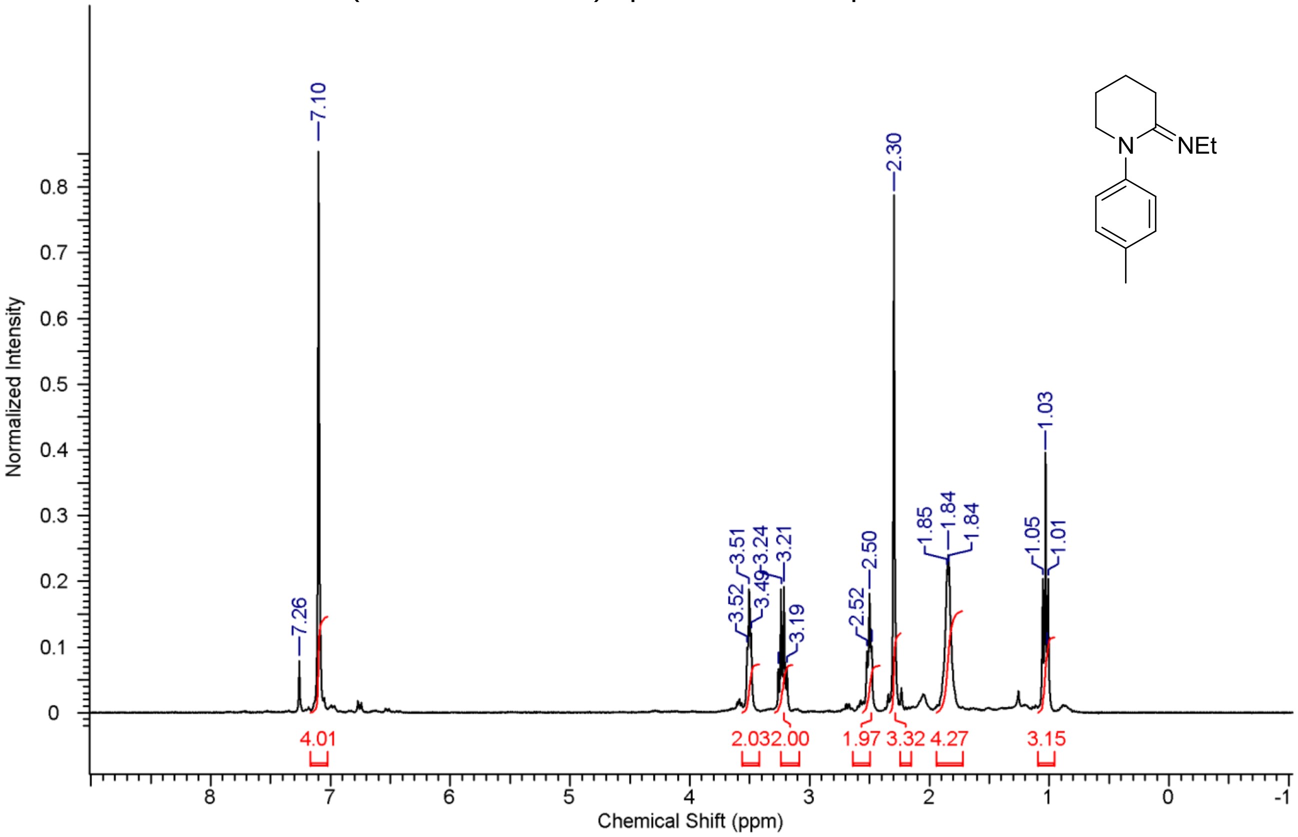

^{13}C NMR (75 MHz, $CDCl_3$) spectrum of compound **5a**

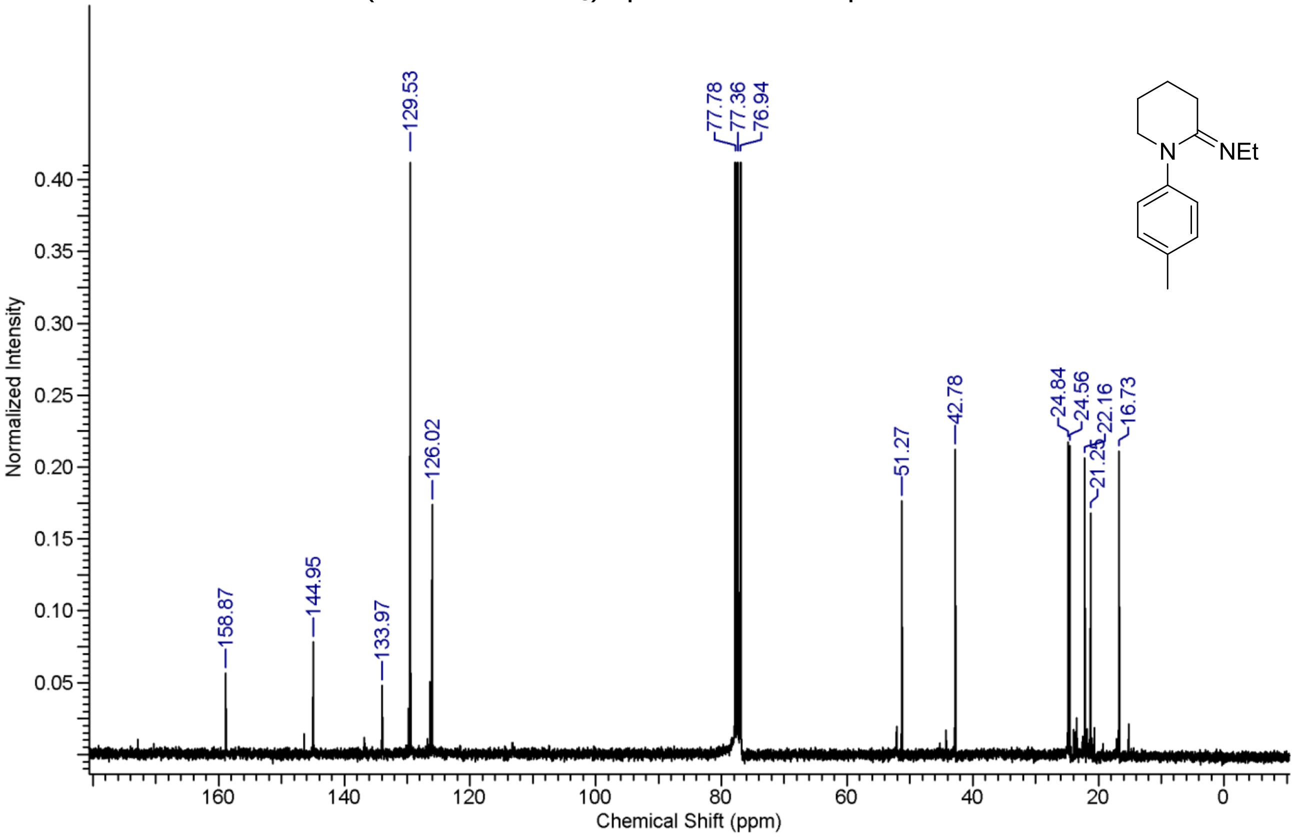

^{1}H NMR (500 MHz, $CDCl_3$) spectrum of compound **4b**

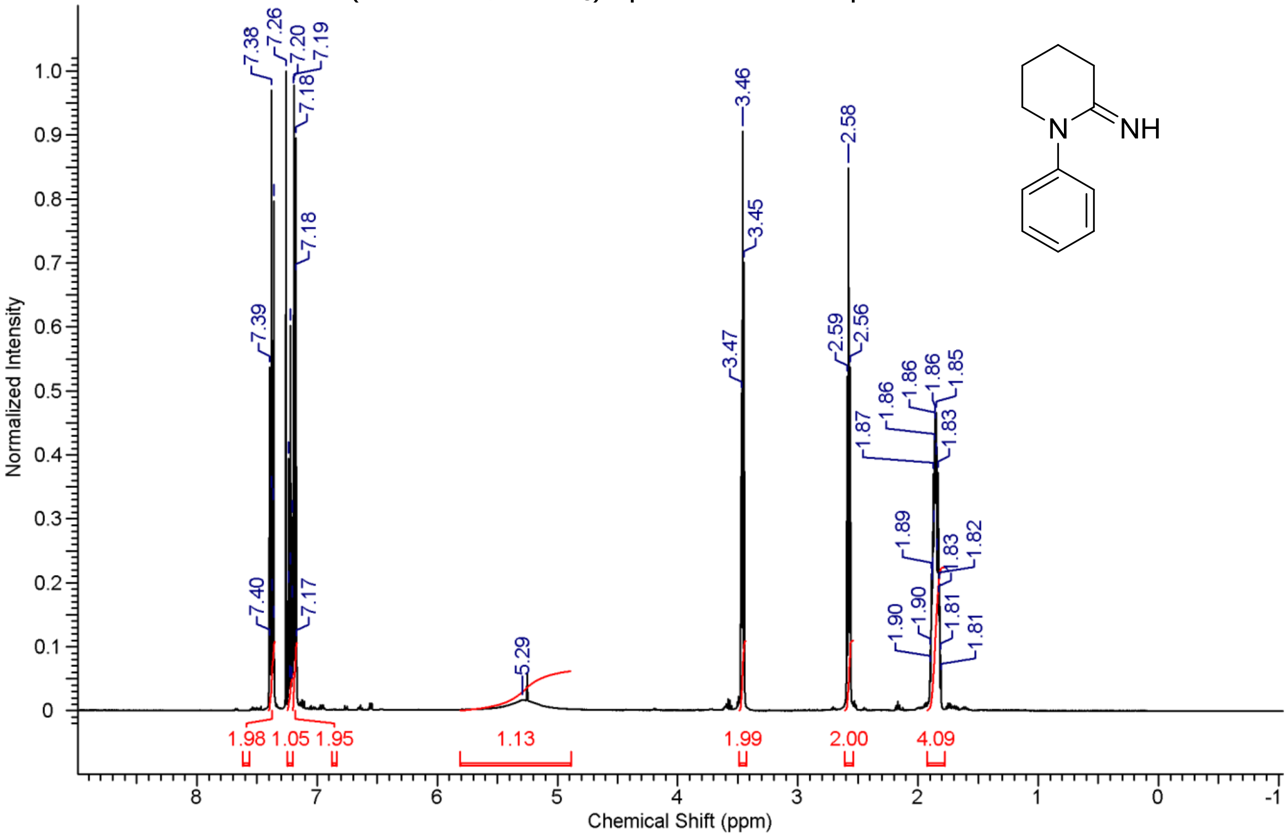

^{13}C NMR (125 MHz, $CDCl_3$) spectrum of compound **4b**

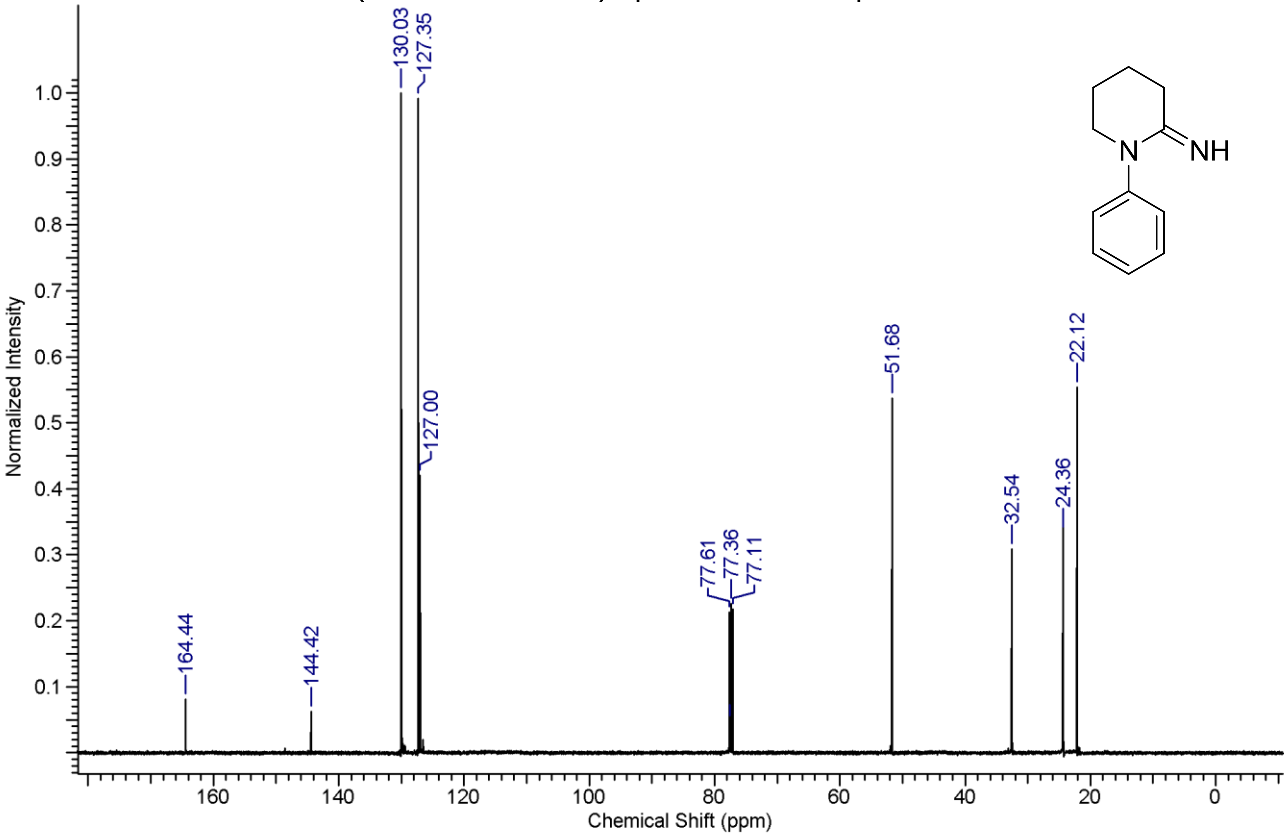

^{1}H NMR (500 MHz, $CDCl_3$) spectrum of compound **4c**

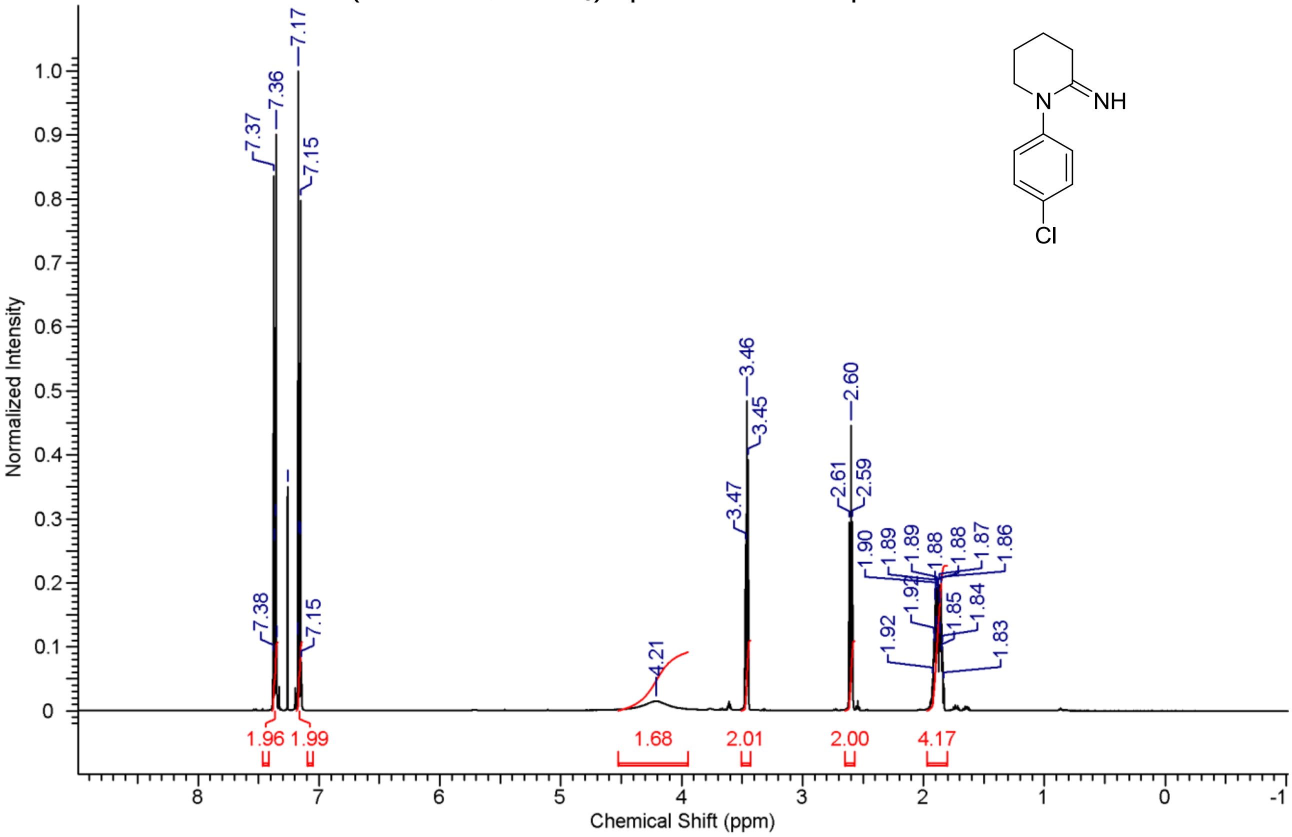

^{13}C NMR (125 MHz, $CDCl_3$) spectrum of compound **4c**

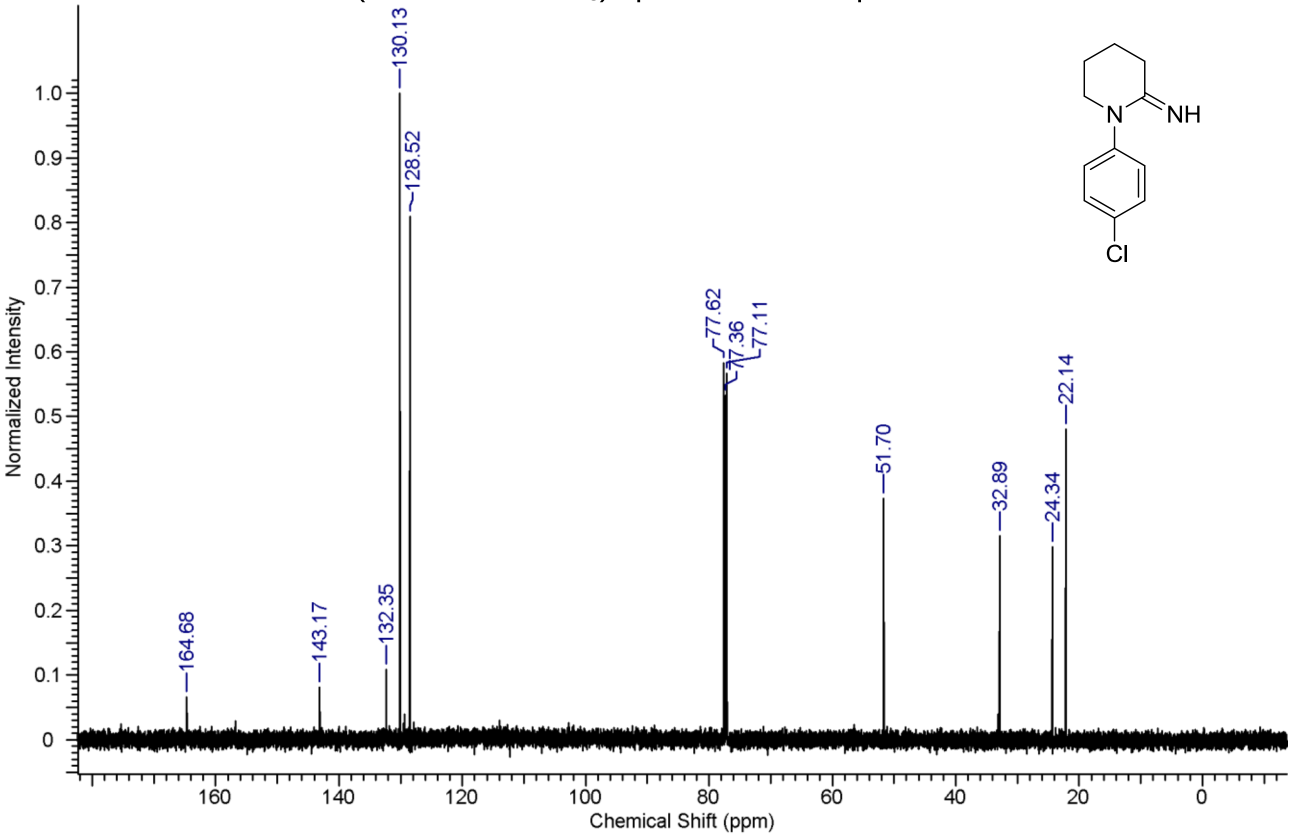

^{1}H NMR (500 MHz, $CDCl_3$) spectrum of compound **4d**

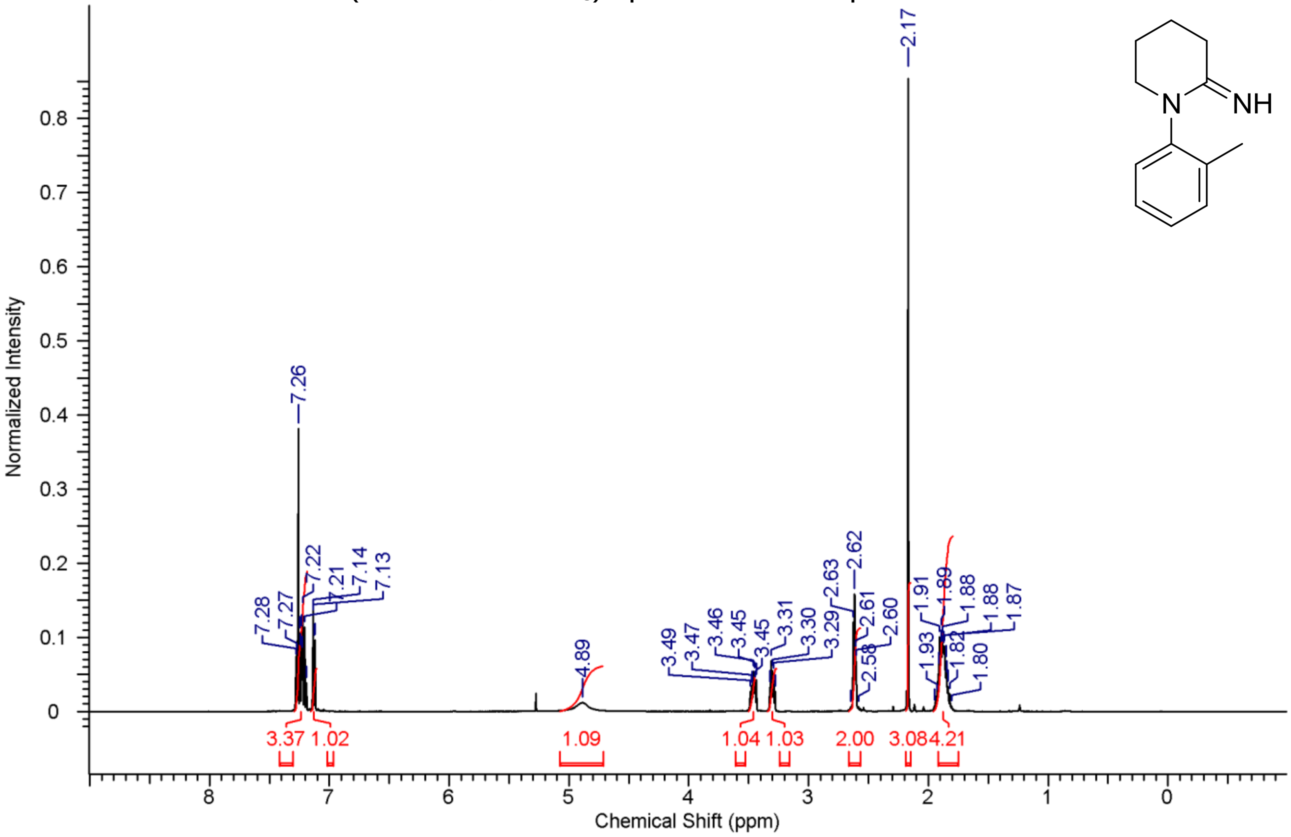

^{13}C NMR (125 MHz, $CDCl_3$) spectrum of compound **4d**

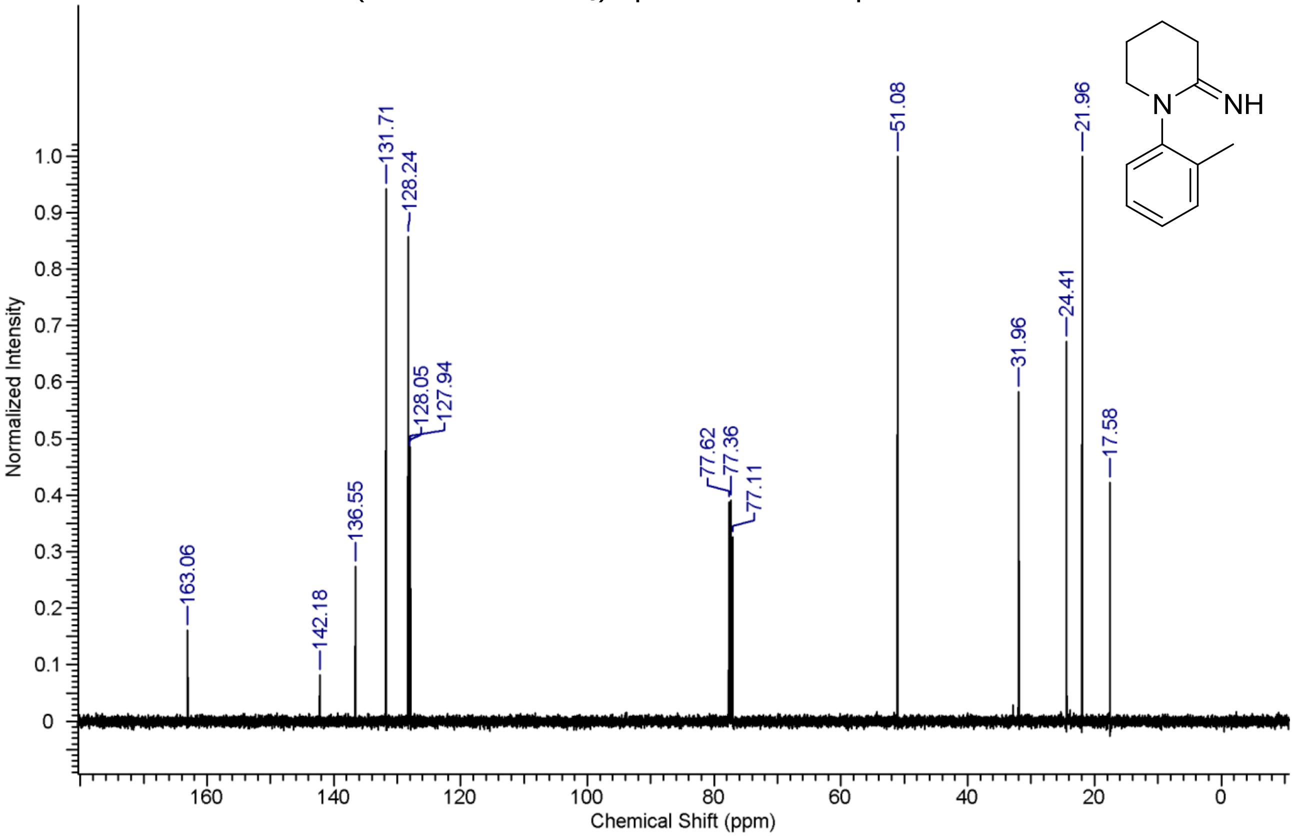

^{1}H NMR (500 MHz, $CDCl_3$) spectrum of compound **4e**

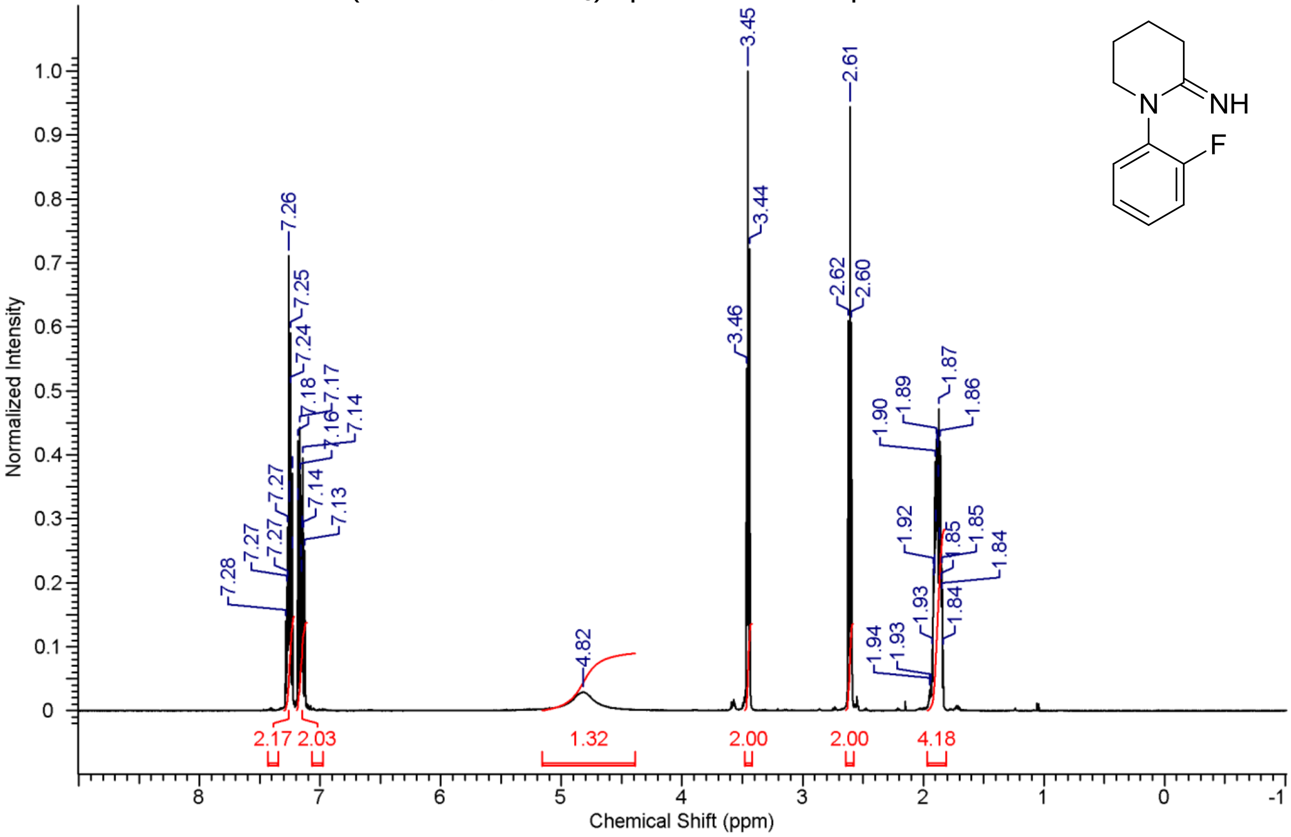

^{13}C NMR (125 MHz, $CDCl_3$) spectrum of compound **4e**

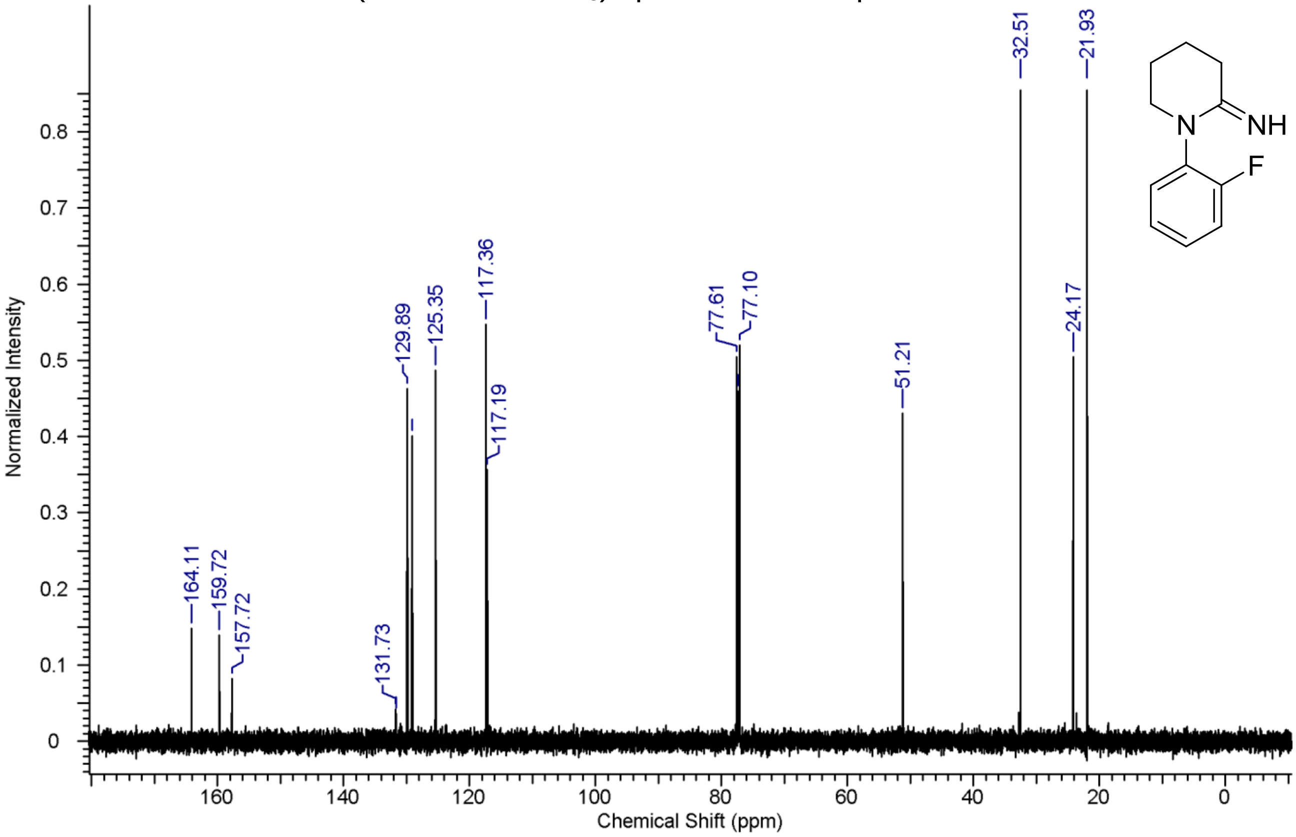

^{1}H NMR (500 MHz, $CDCl_3$) spectrum of compound **4f**

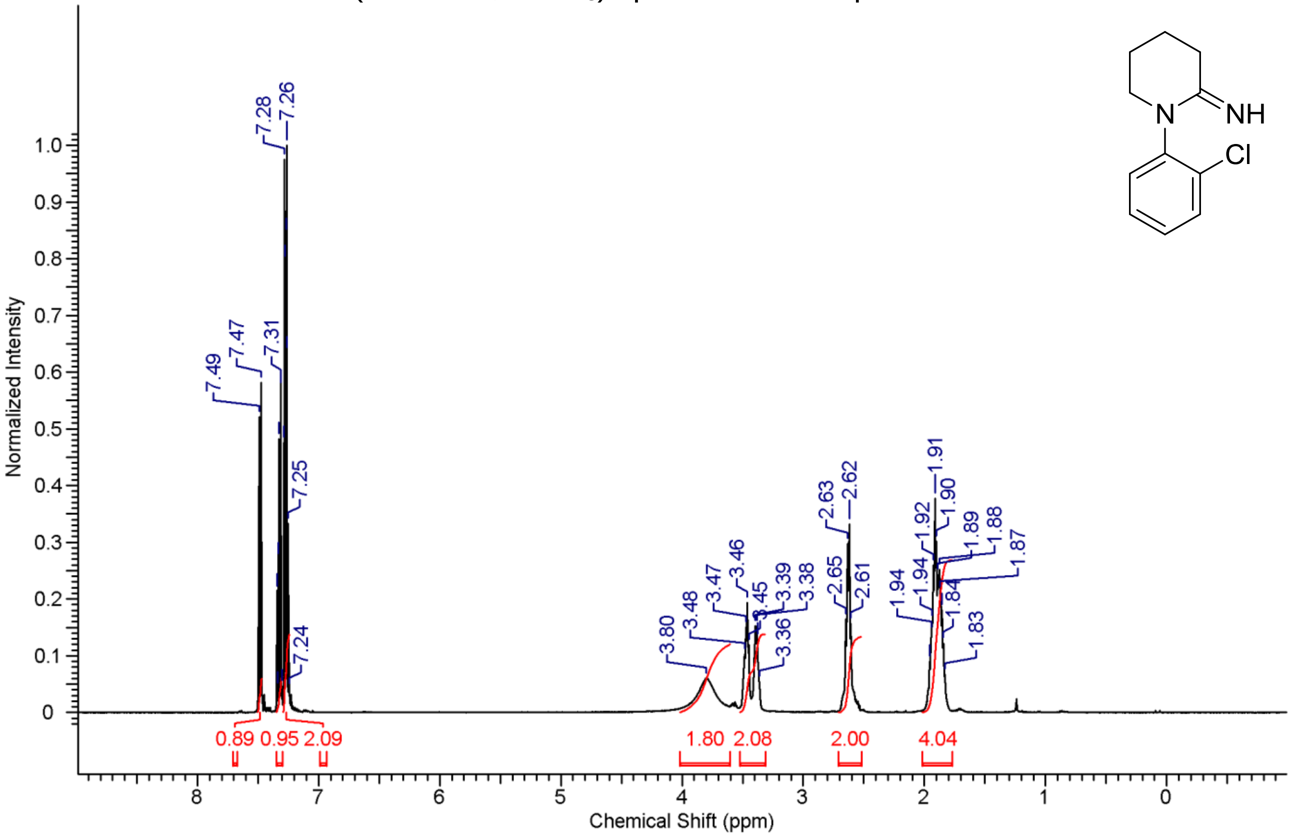

^{13}C NMR (125 MHz, $CDCl_3$) spectrum of compound **4f**

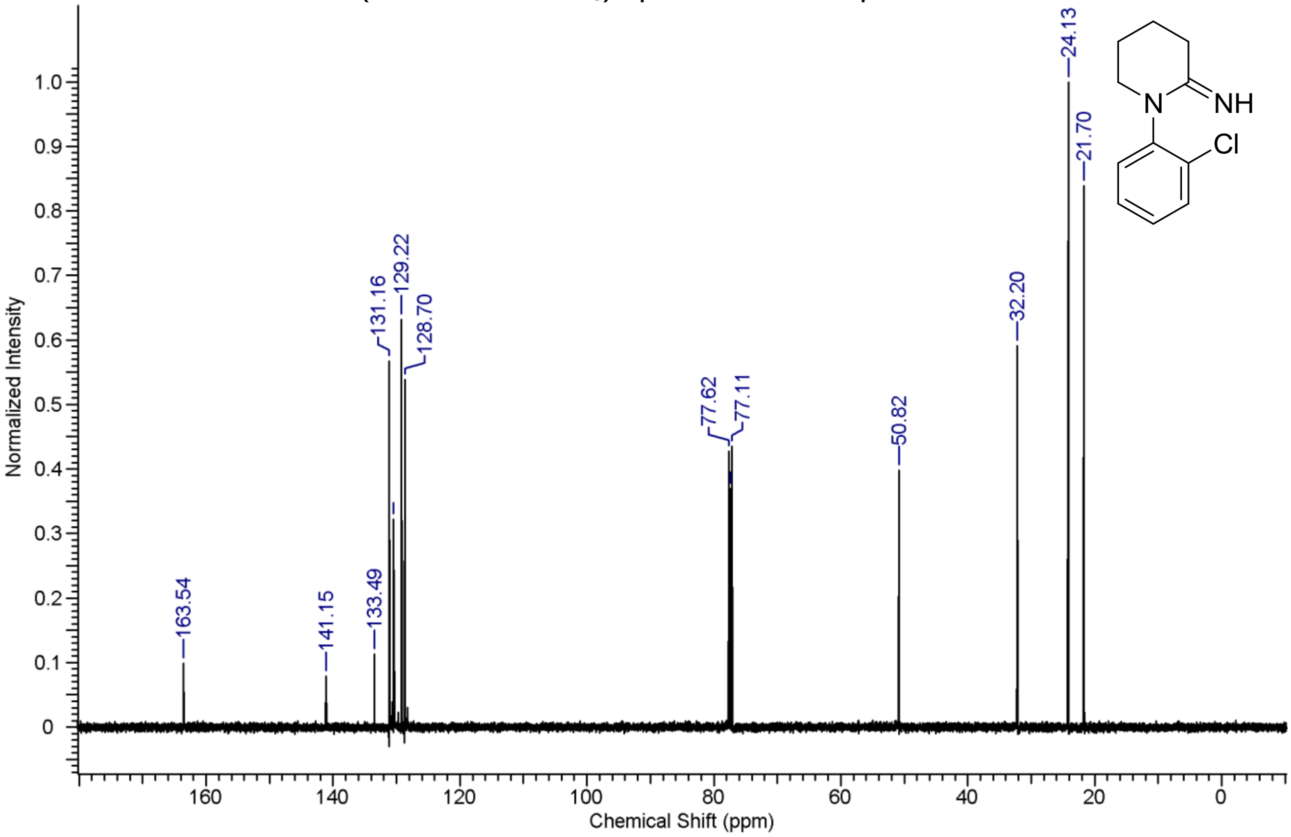

^{1}H NMR (600 MHz, $CDCl_3$) spectrum of compound **4g**

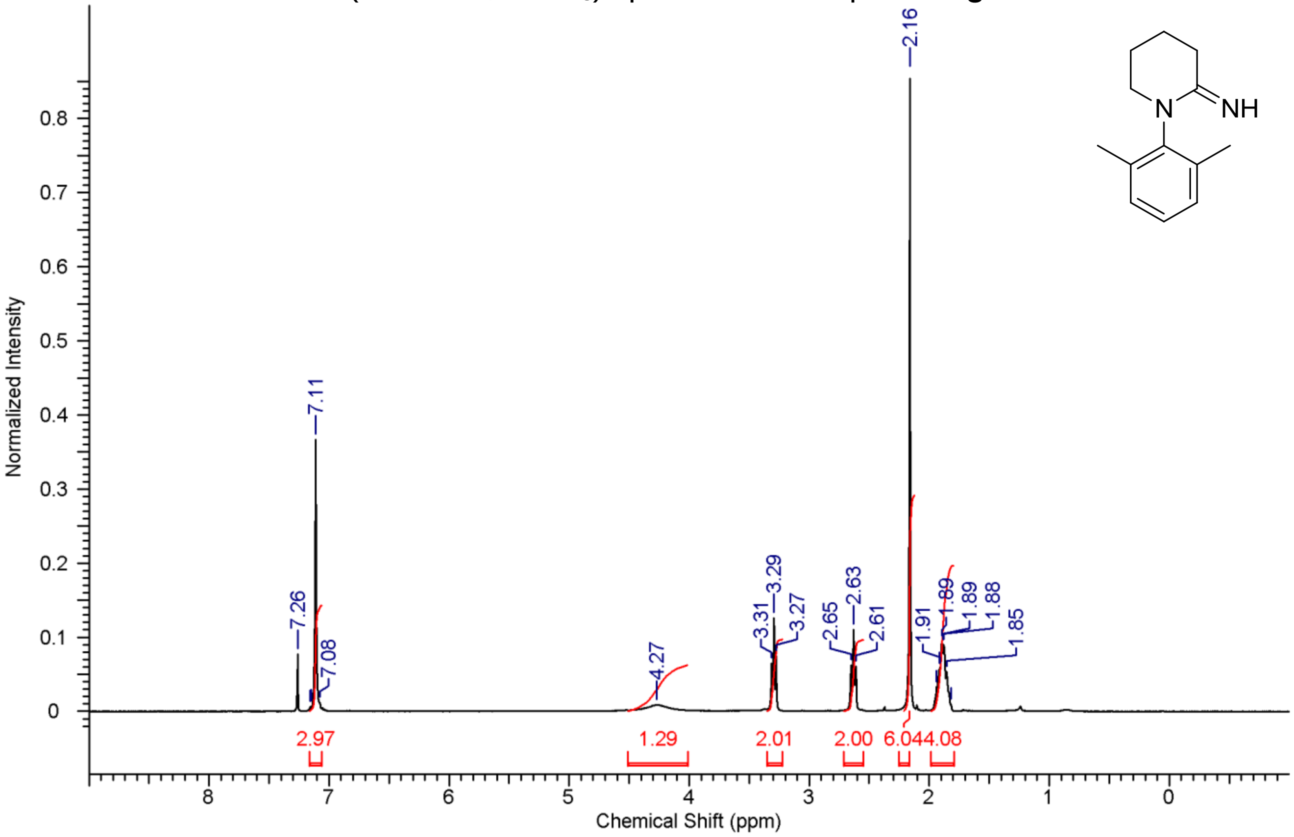

^{13}C NMR (150 MHz, $CDCl_3$) spectrum of compound **4g**

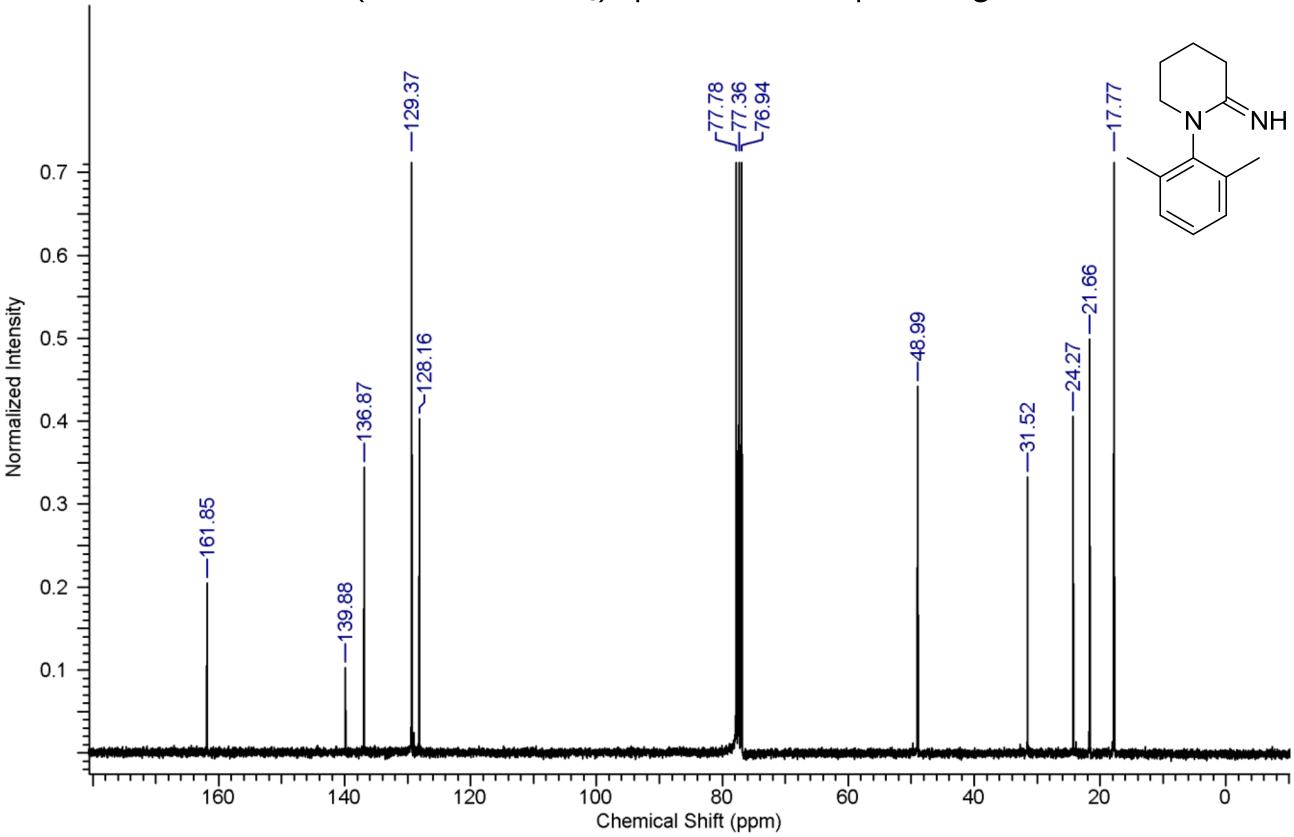

^{1}H NMR (500 MHz, $CDCl_3$) spectrum of compound **7a**

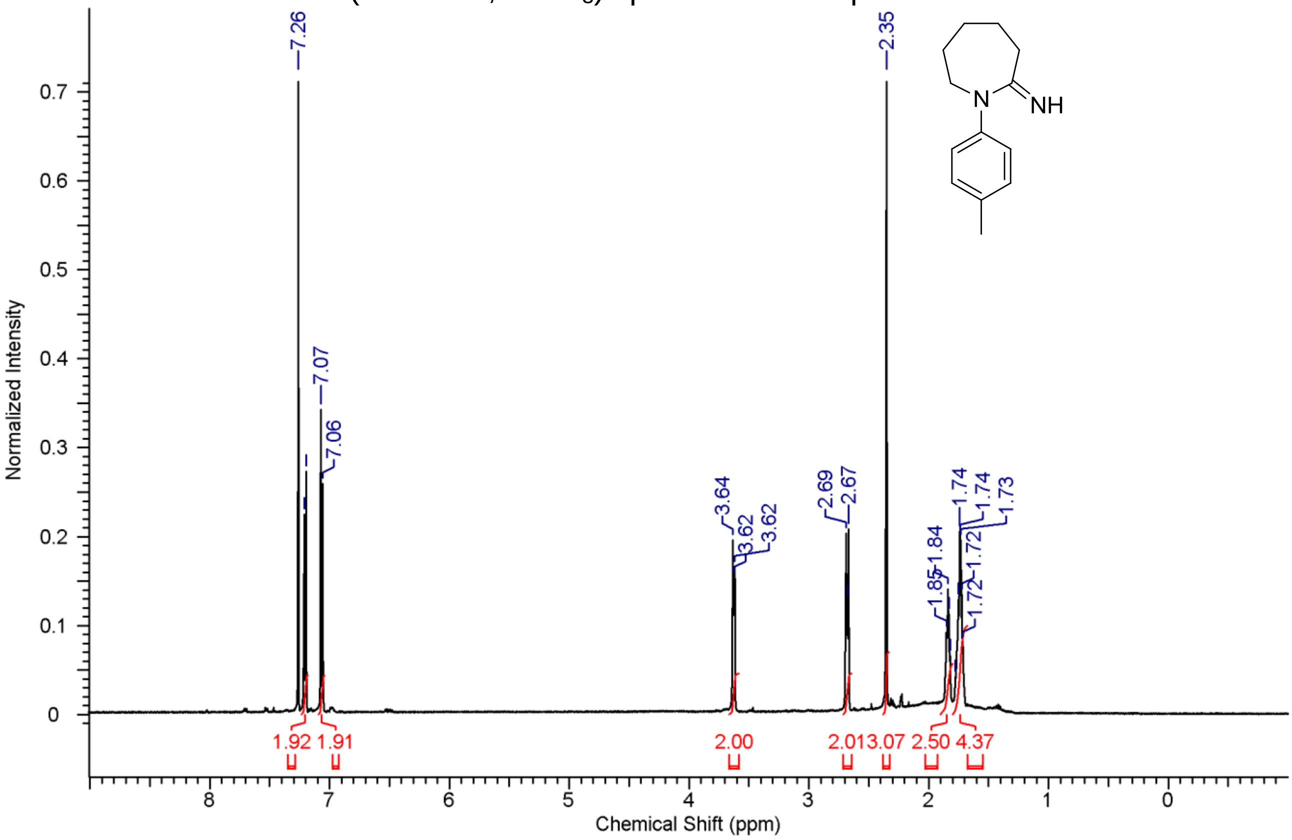

^{13}C NMR (125 MHz, $CDCl_3$) spectrum of compound **7a**

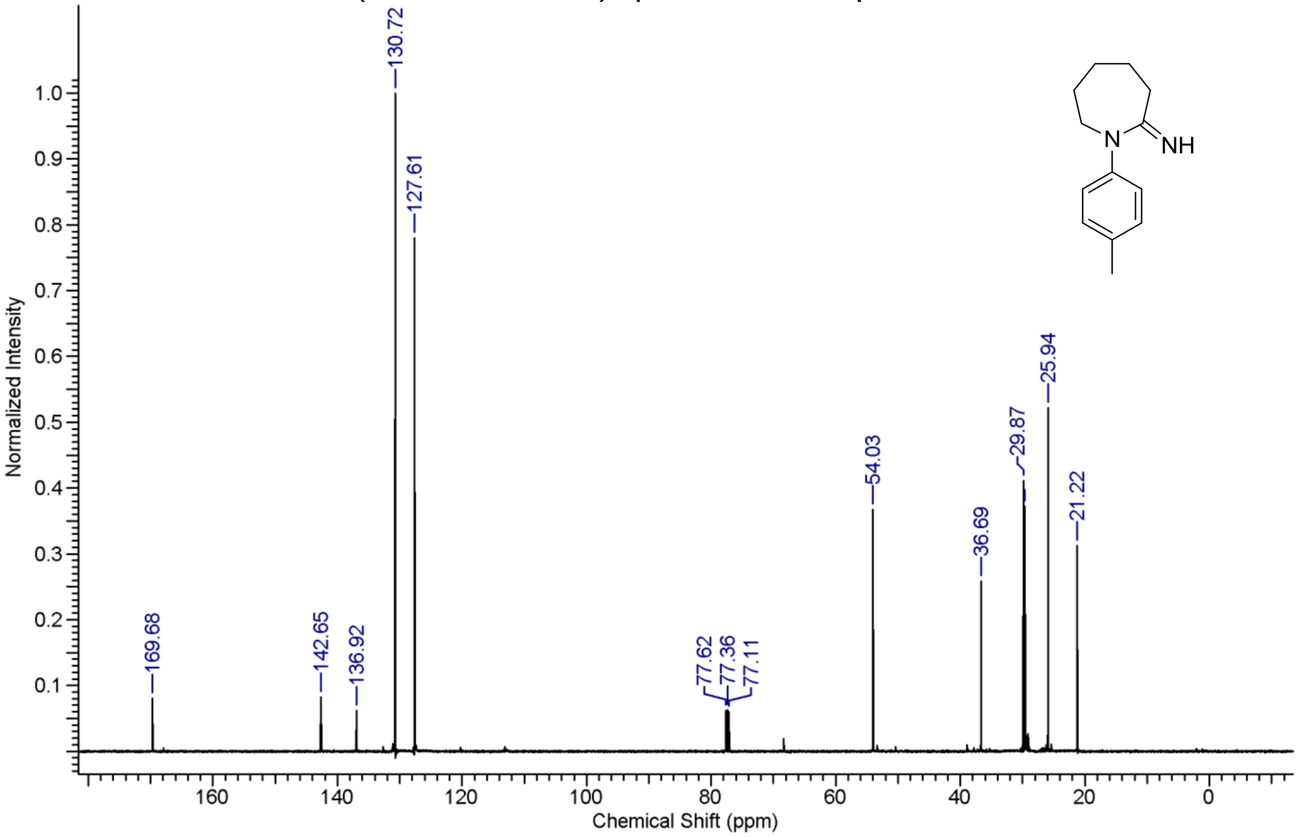

^{1}H NMR (600 MHz, $CDCl_3$) spectrum of compound **8a**

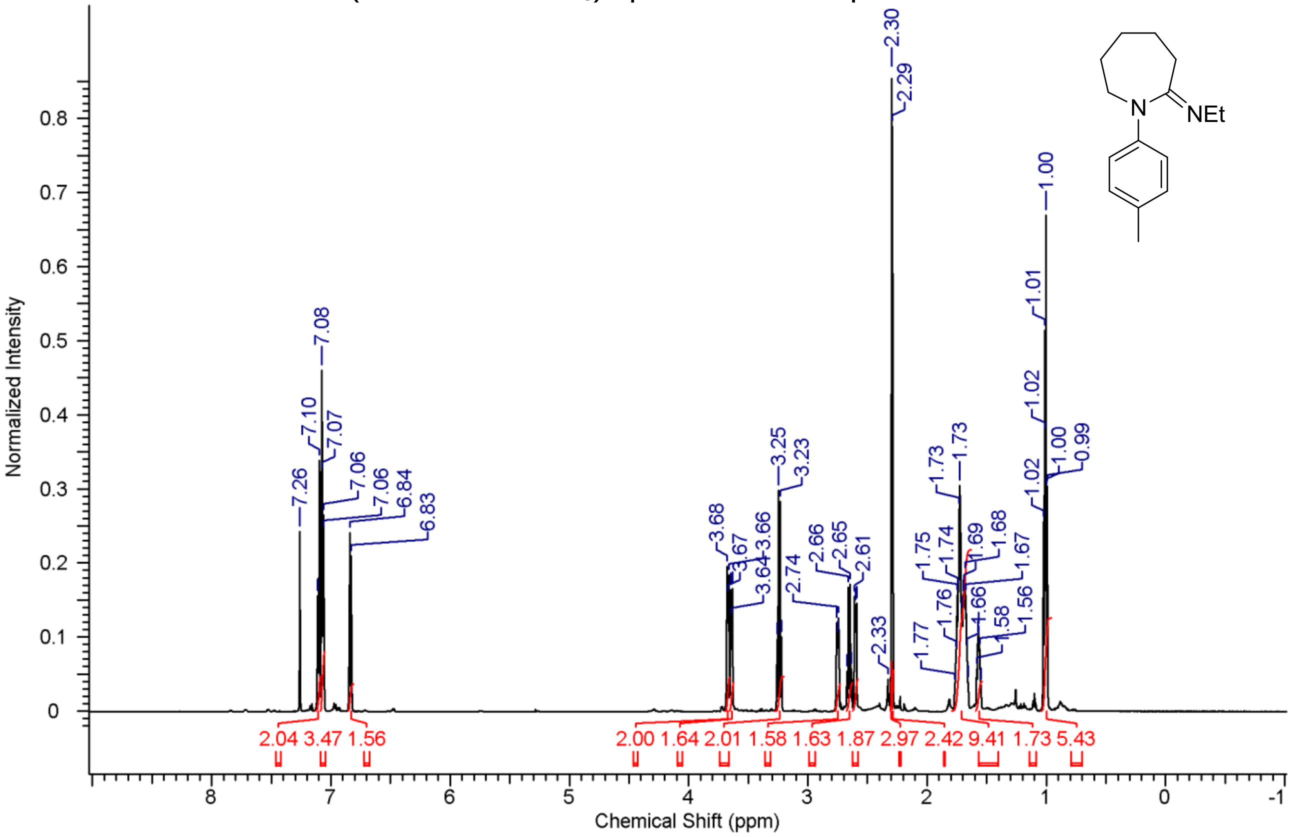

^{13}C NMR (150 MHz, $CDCl_3$) spectrum of compound **8a**

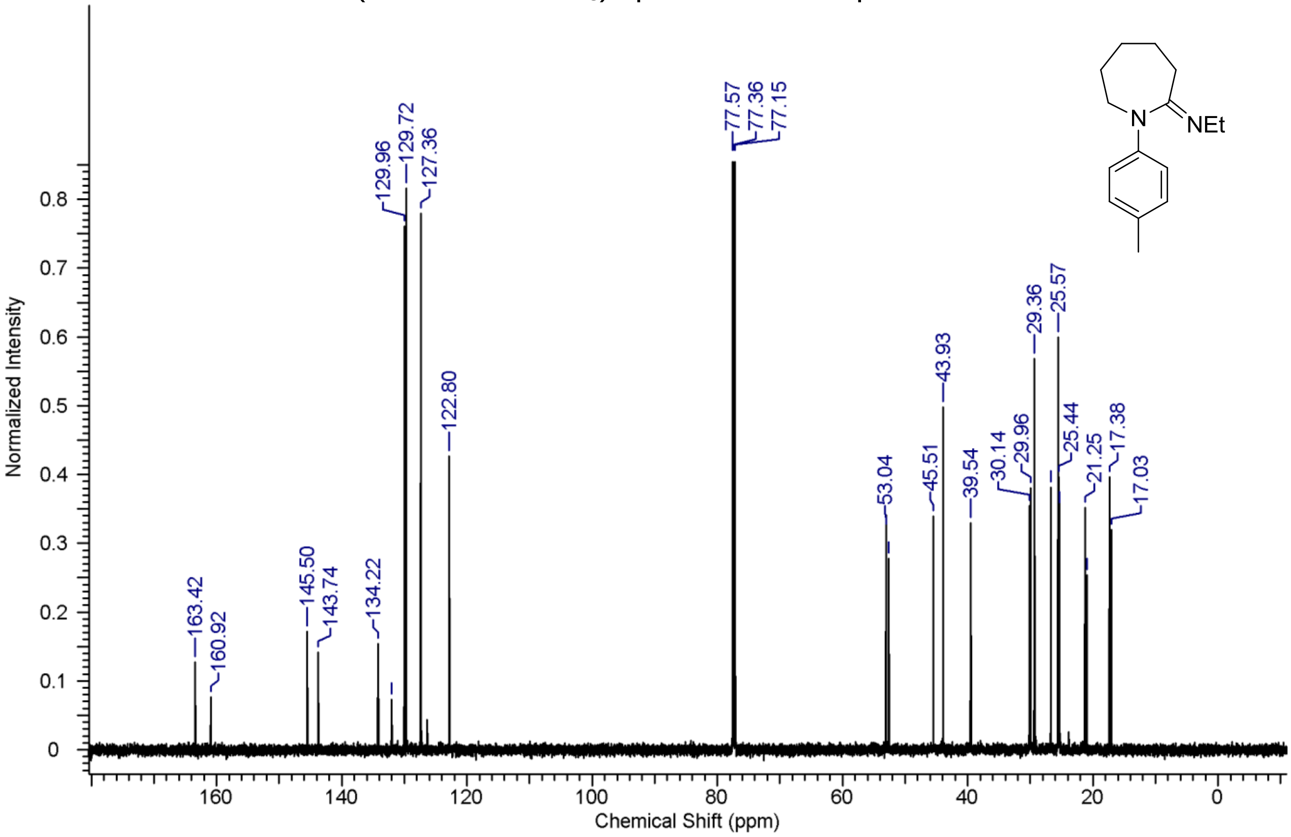

^{1}H NMR (500 MHz, $CDCl_3$) spectrum of compound **7b**

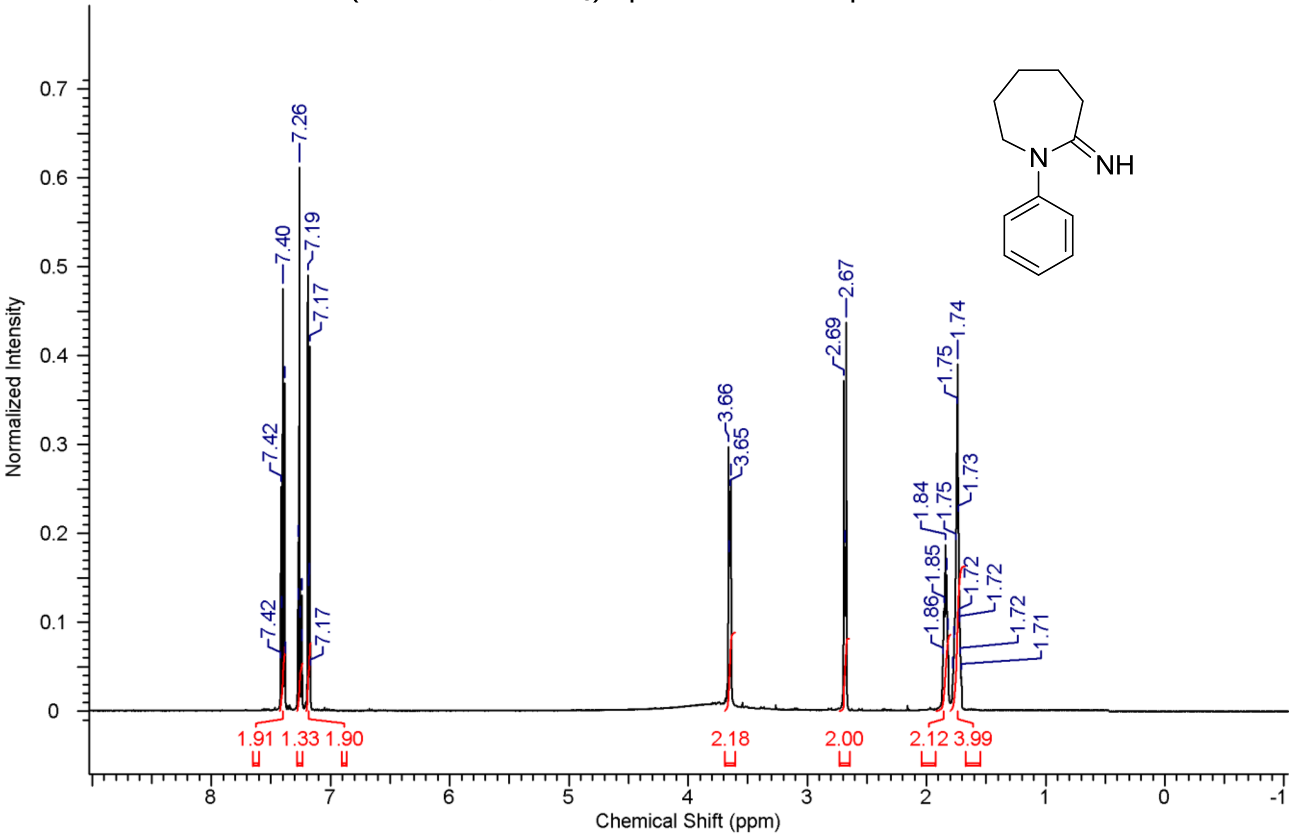

^{13}C NMR (125 MHz, $CDCl_3$) spectrum of compound **7b**

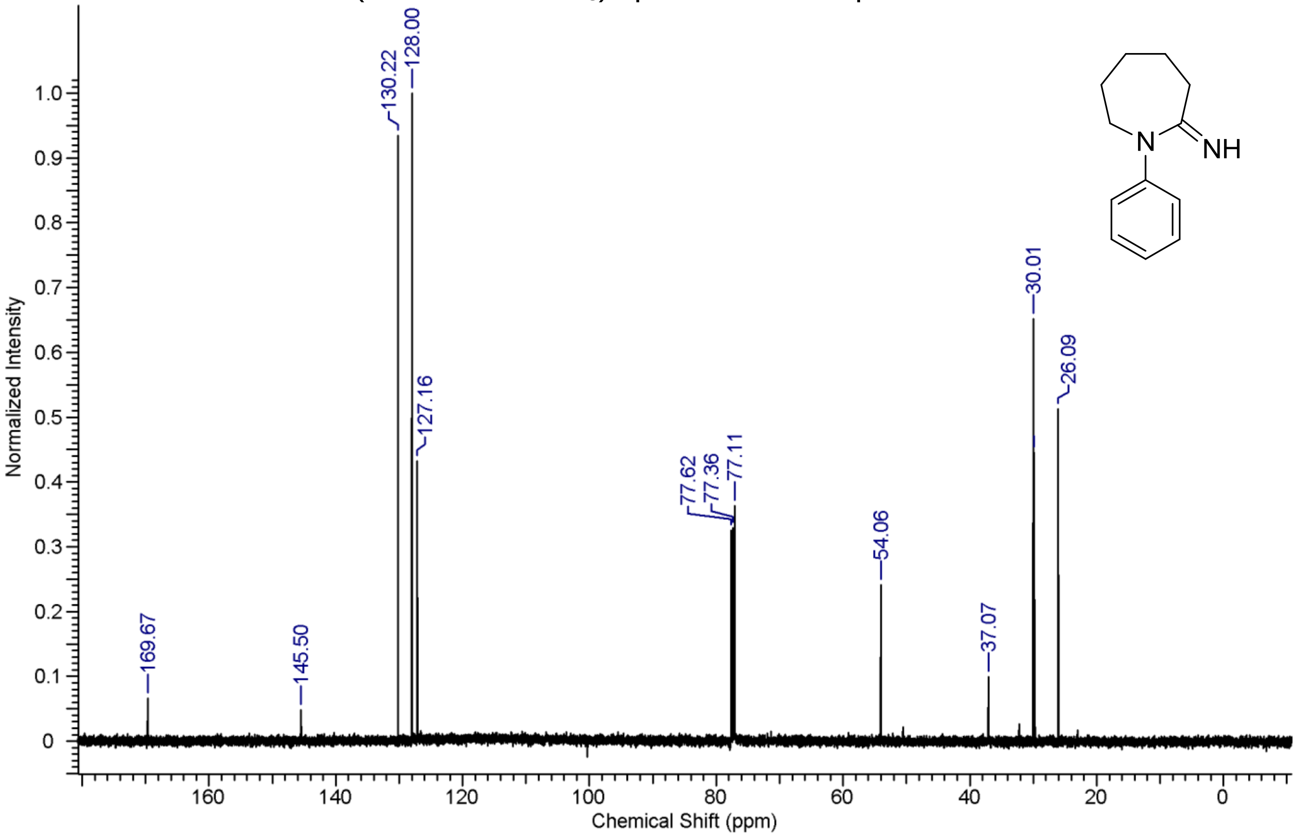

^{1}H NMR (500 MHz, $CDCl_3$) spectrum of compound **7c**

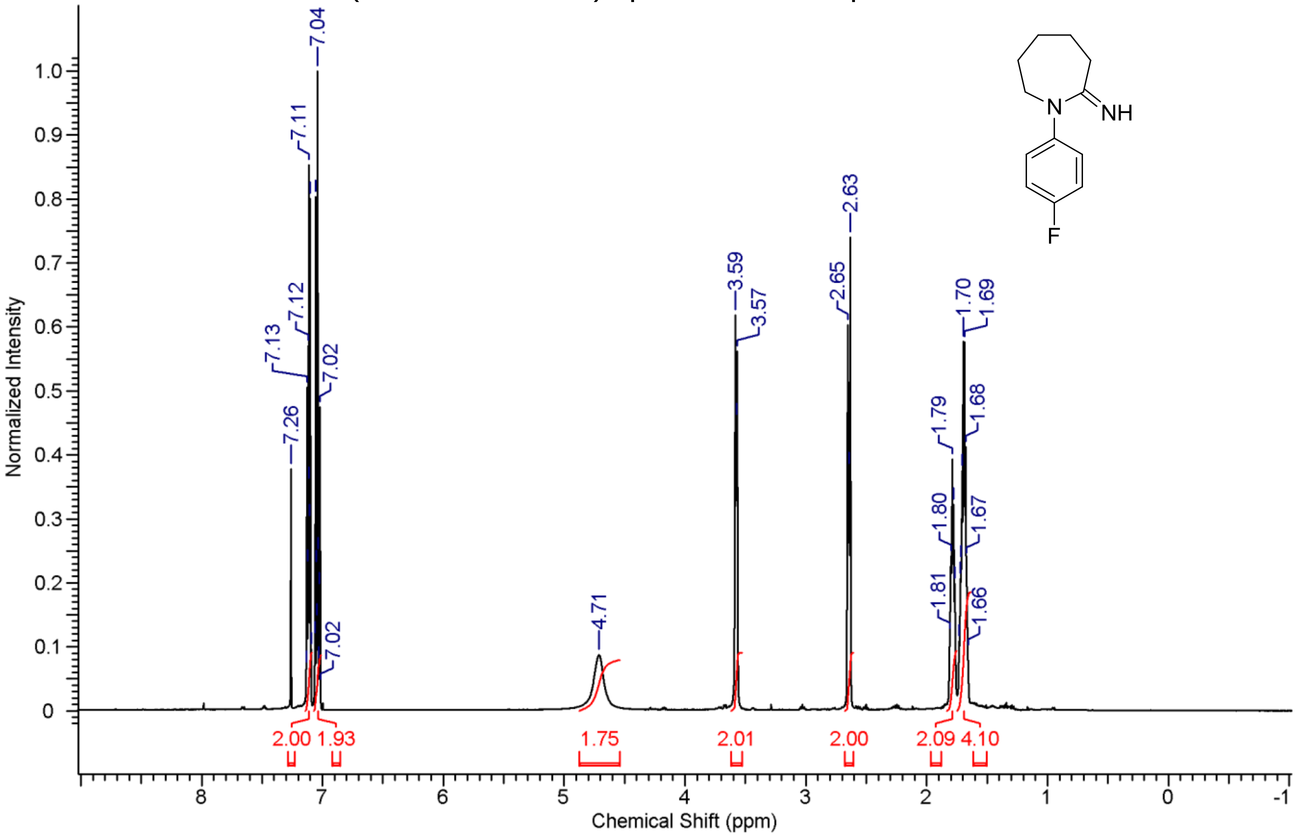

^{13}C NMR (125 MHz, $CDCl_3$) spectrum of compound **7c**

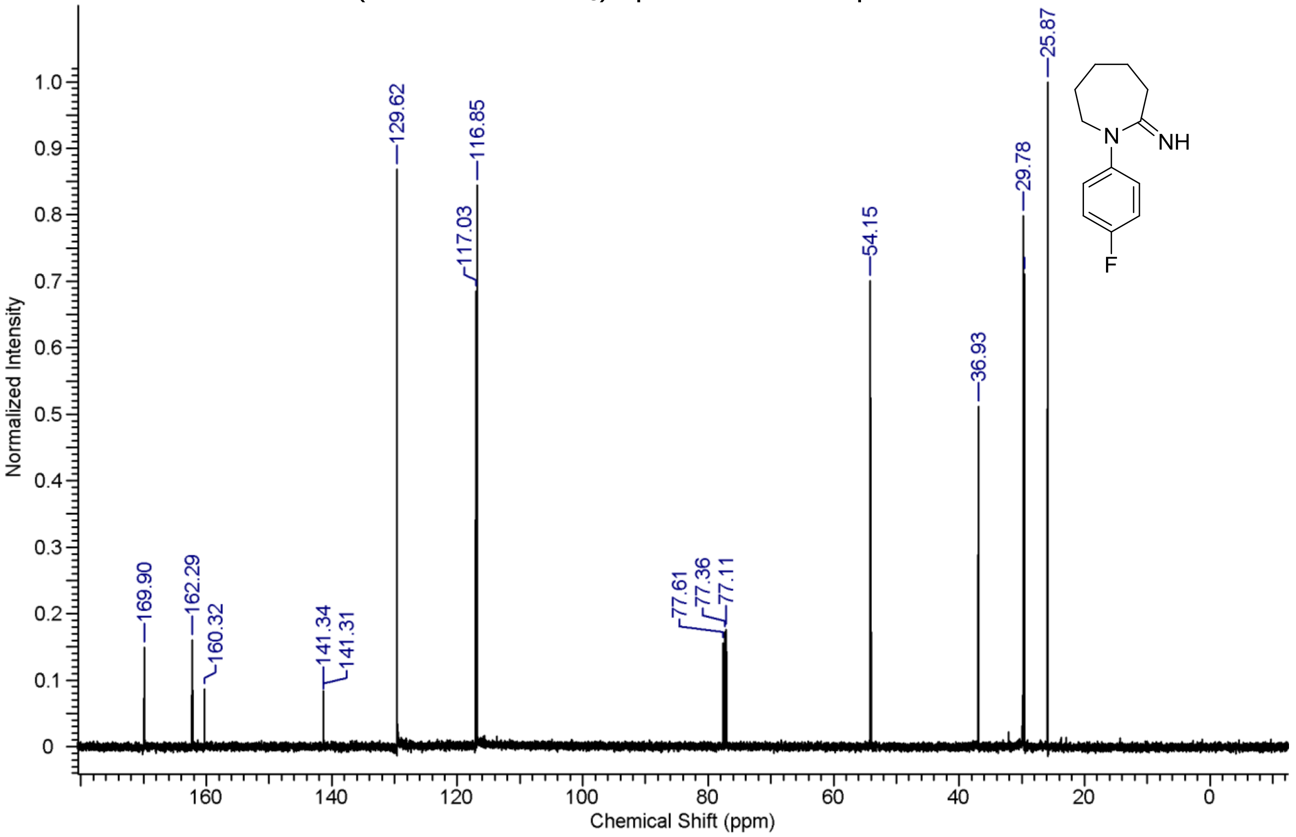

^{1}H NMR (500 MHz, $CDCl_3$) spectrum of compound **7d**

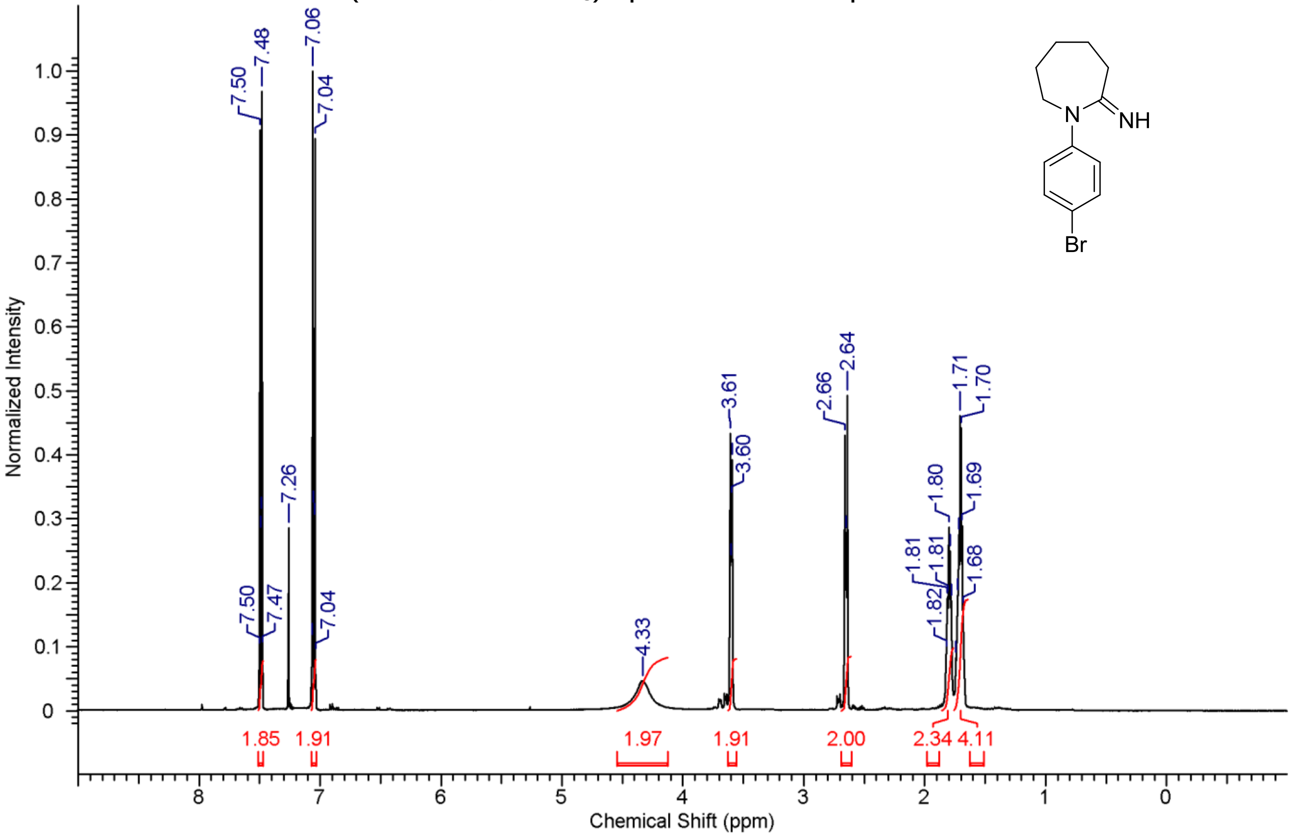

^{13}C NMR (125 MHz, $CDCl_3$) spectrum of compound **7d**

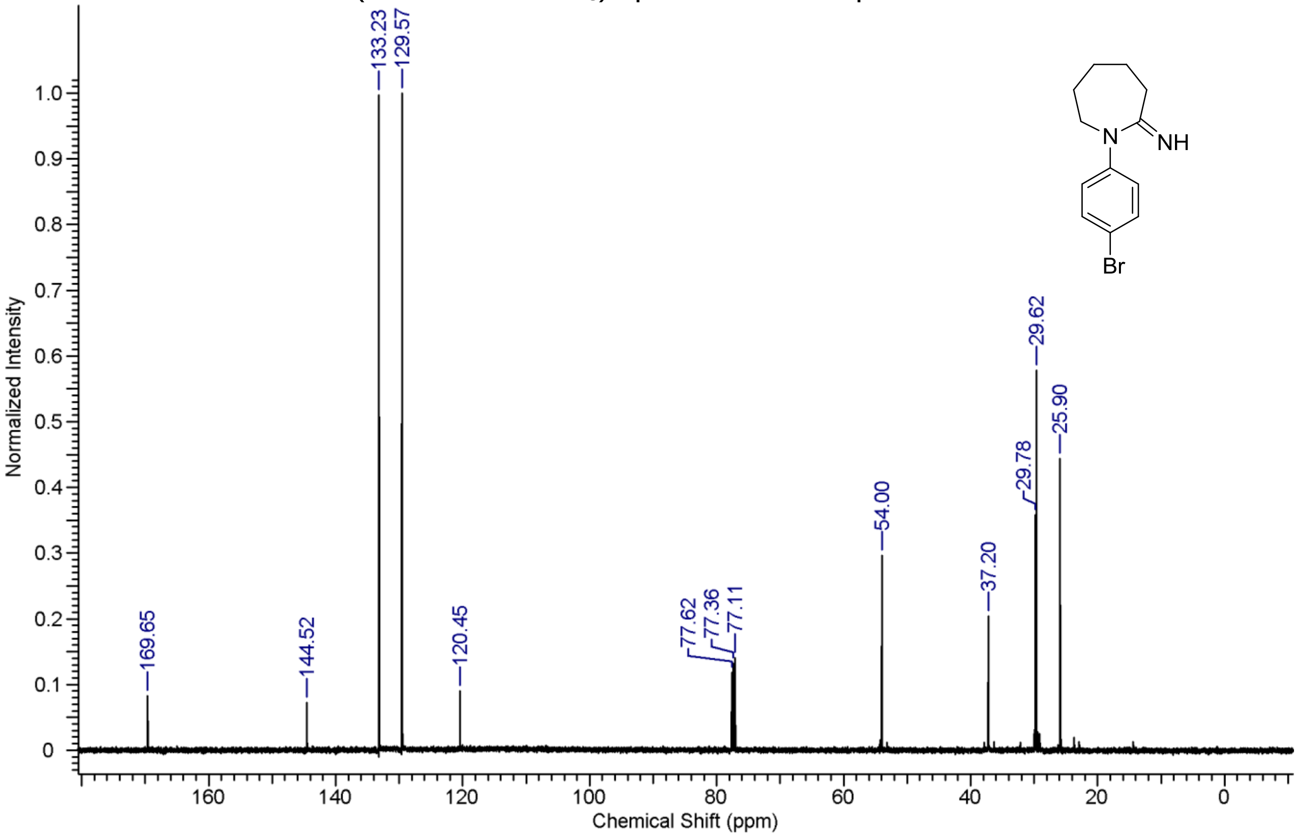

^{1}H NMR (500 MHz, $CDCl_3$) spectrum of compound **7e**

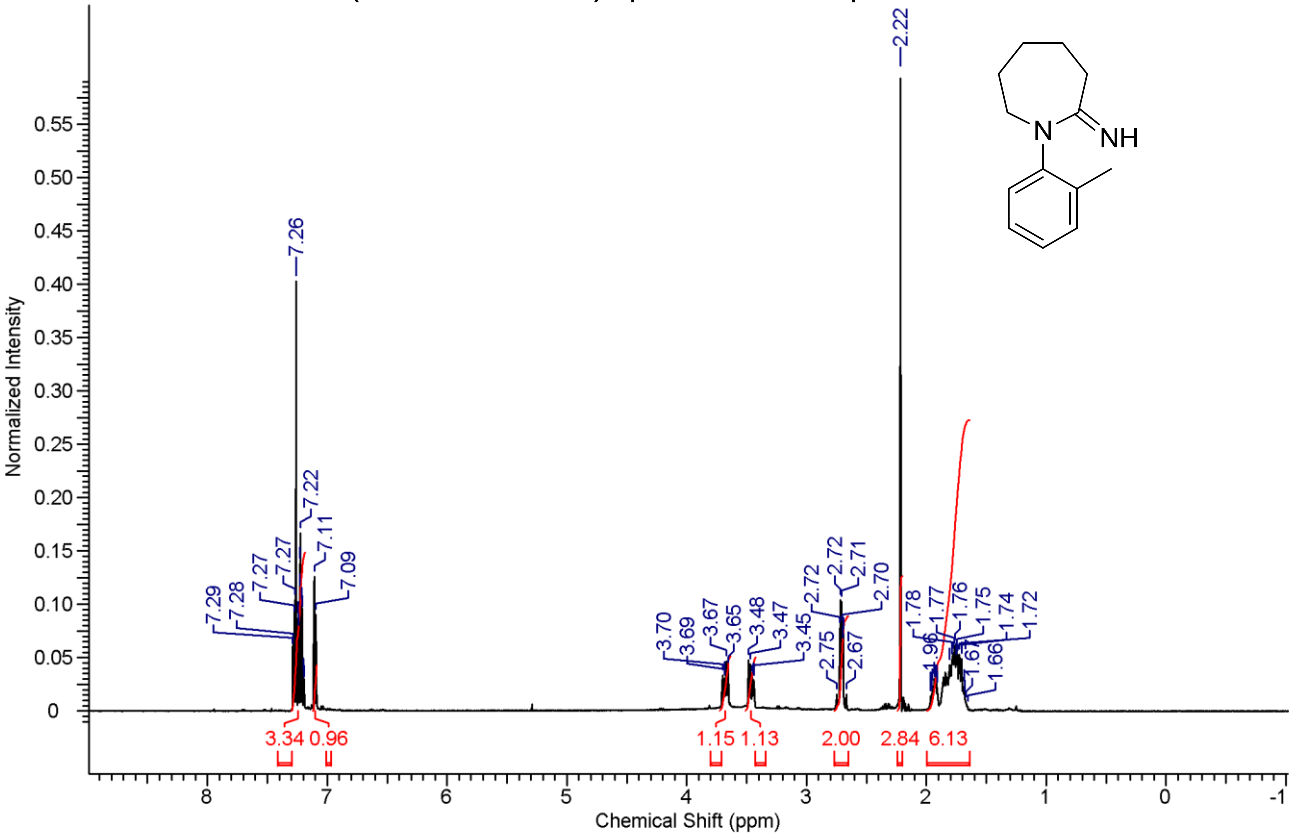

^{13}C NMR (125 MHz, $CDCl_3$) spectrum of compound **7e**

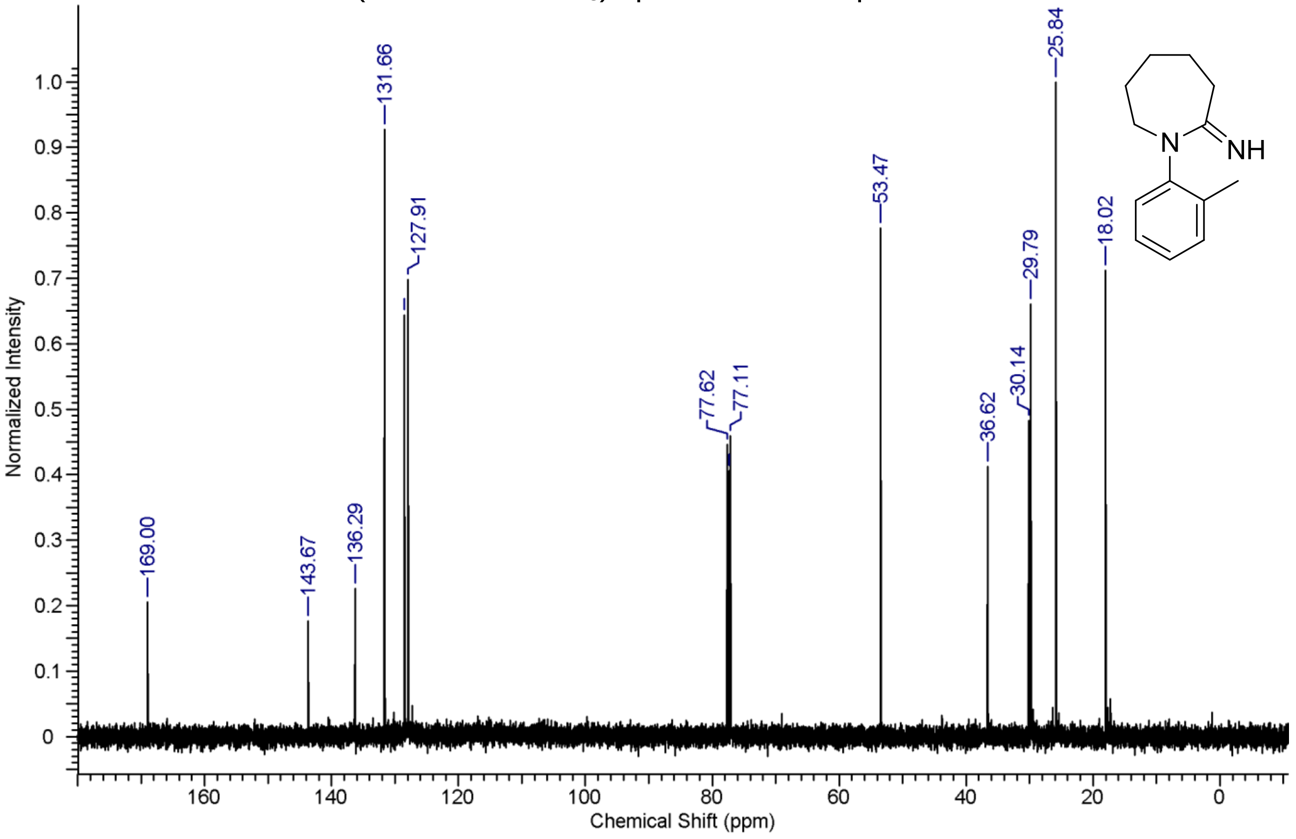

^{1}H NMR (500 MHz, $CDCl_3$) spectrum of compound **7f**

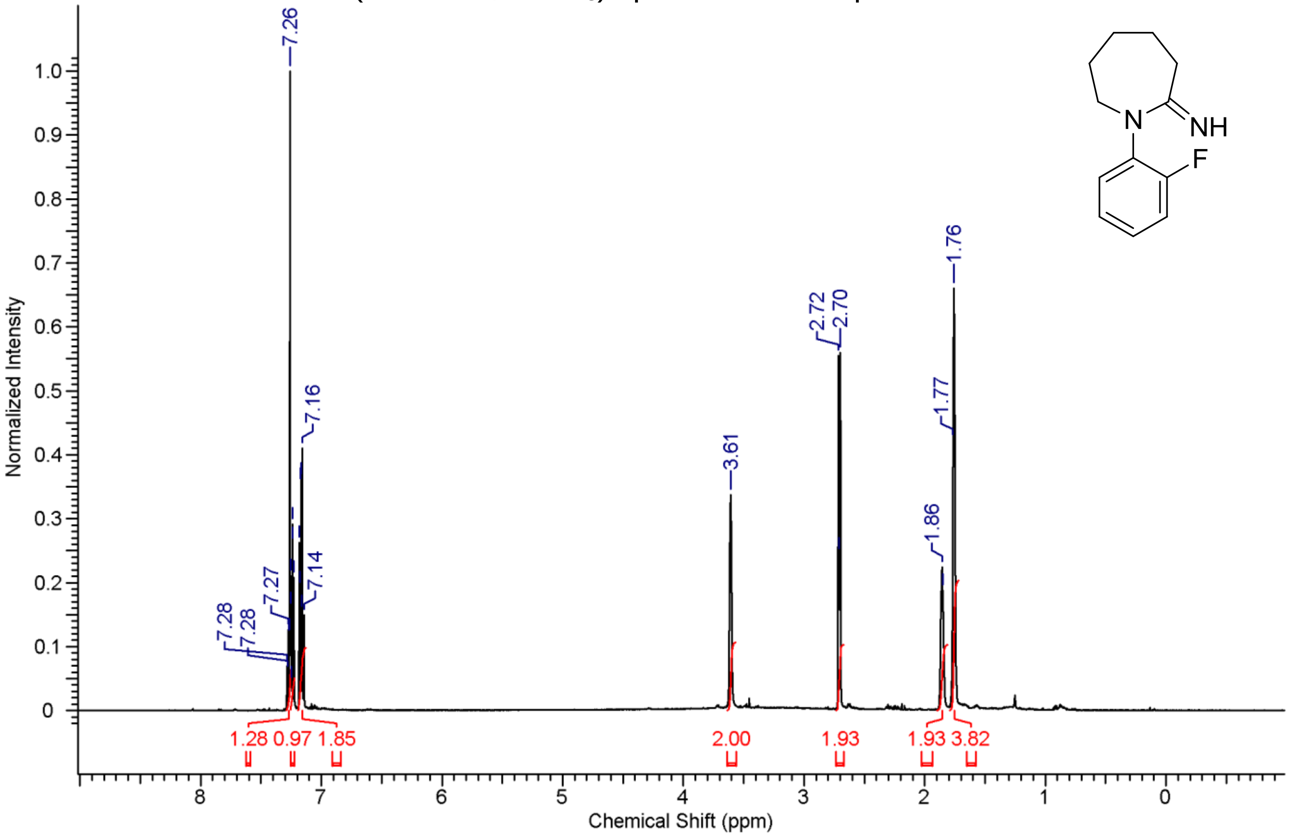

^{13}C NMR (125 MHz, $CDCl_3$) spectrum of compound **7f**

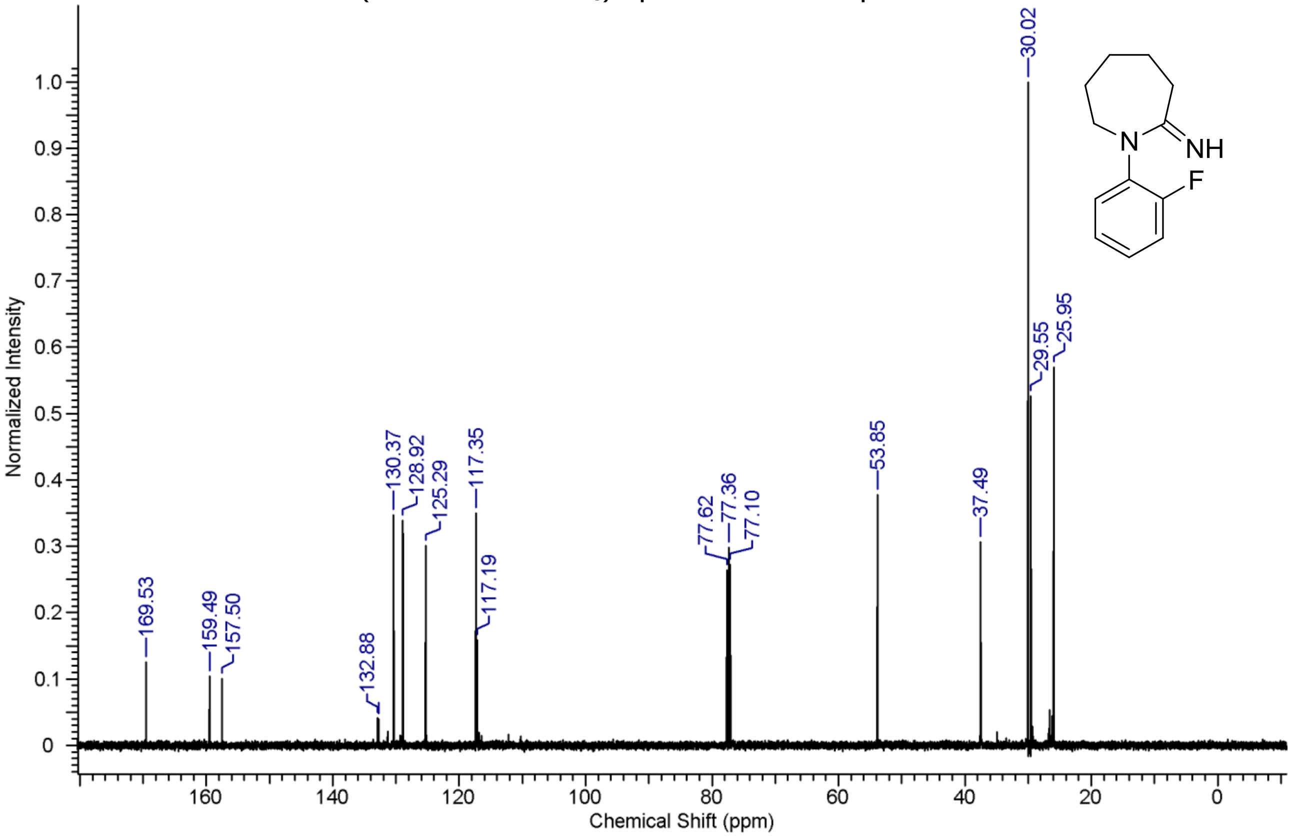

^1H NMR (500 MHz, $CDCl_3$) spectrum of compound **7g**

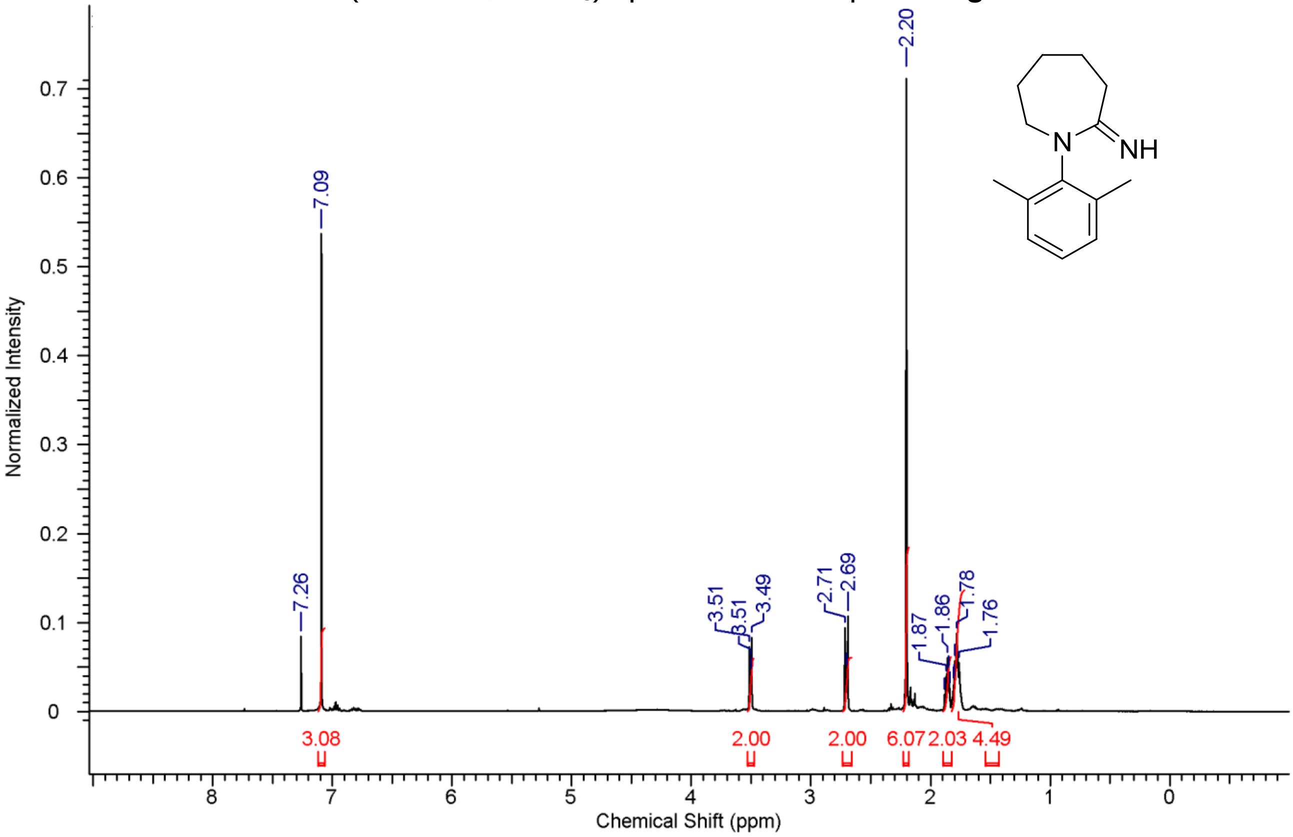

^{13}C NMR (125 MHz, $CDCl_3$) spectrum of compound **7g**

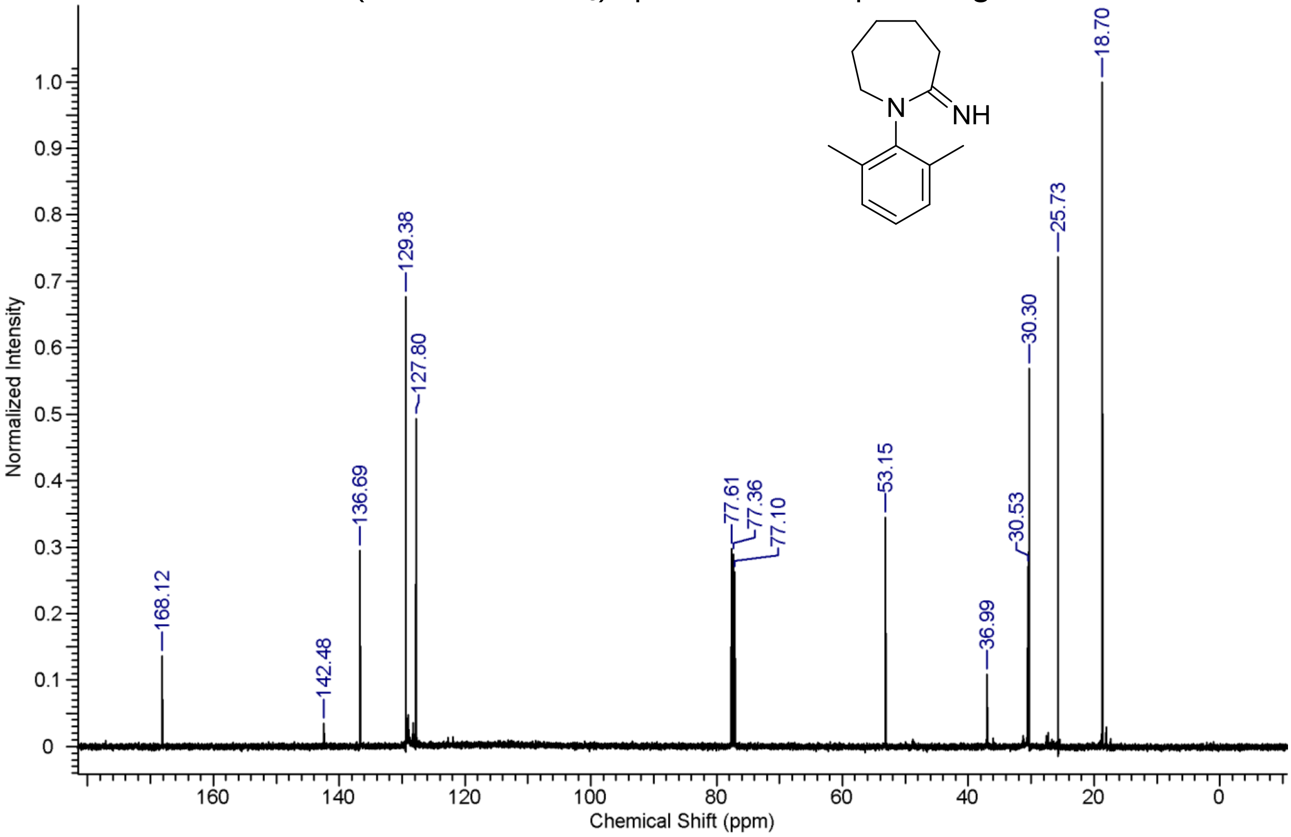

^{1}H NMR (600 MHz, $CDCl_3$) spectrum of compound **1g**

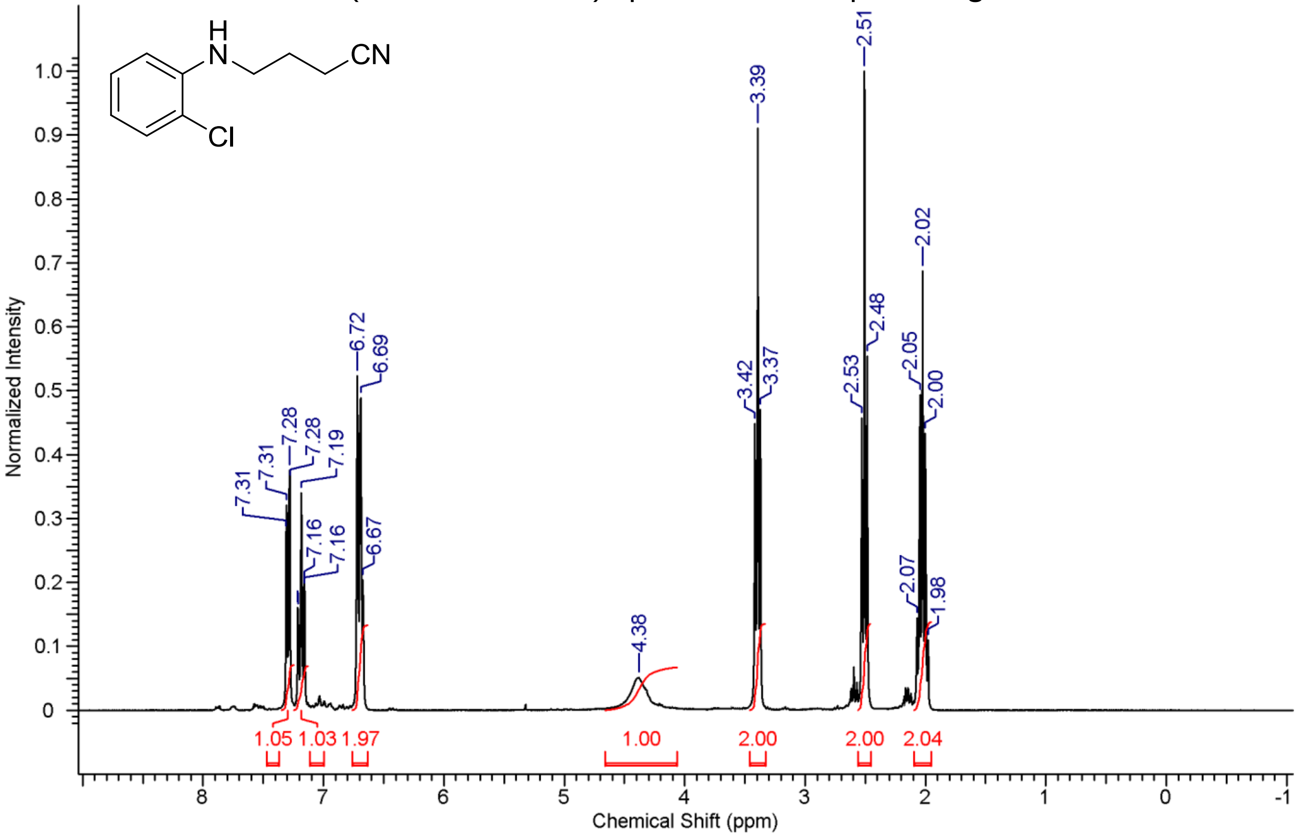

^{13}C NMR (150 MHz, $CDCl_3$) spectrum of compound **1g**

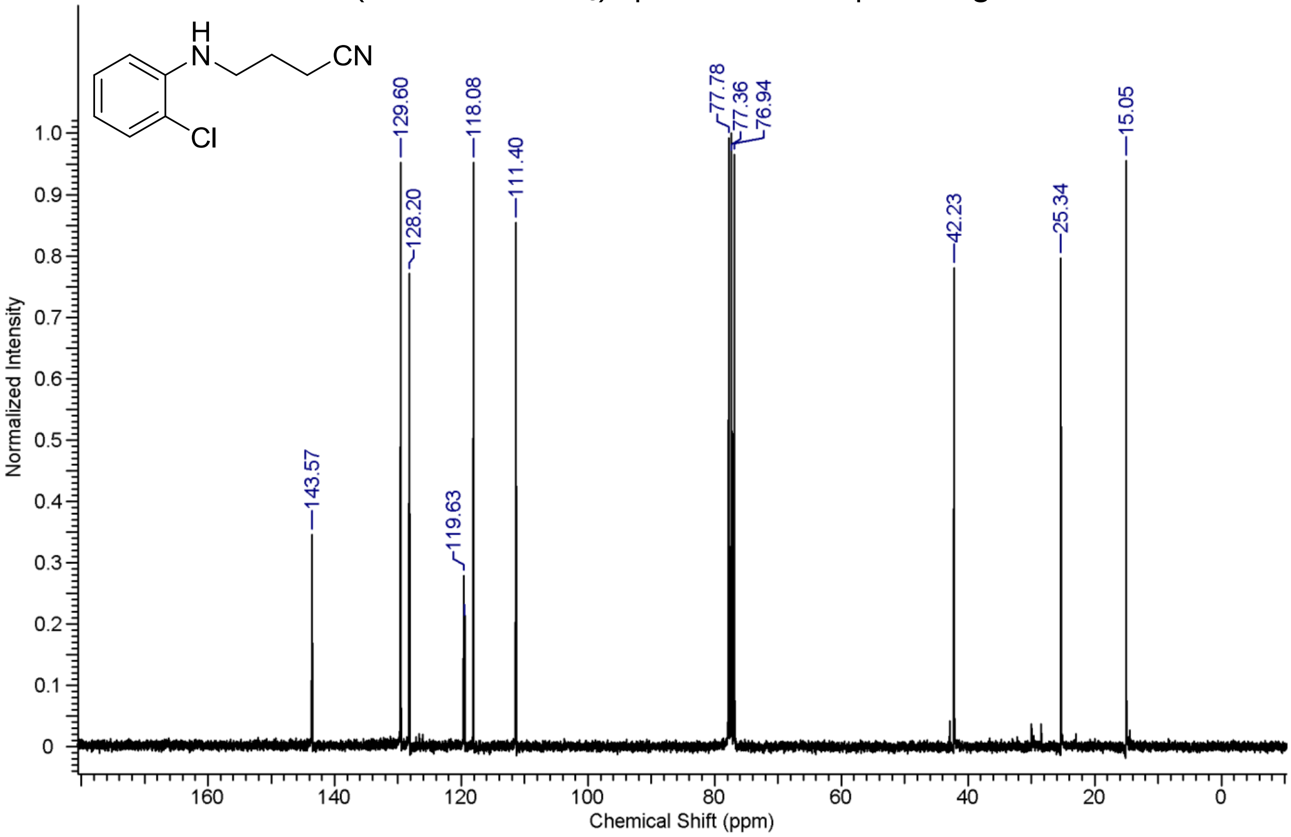

^1H NMR (600 MHz, $CDCl_3$) spectrum of compound **1h**

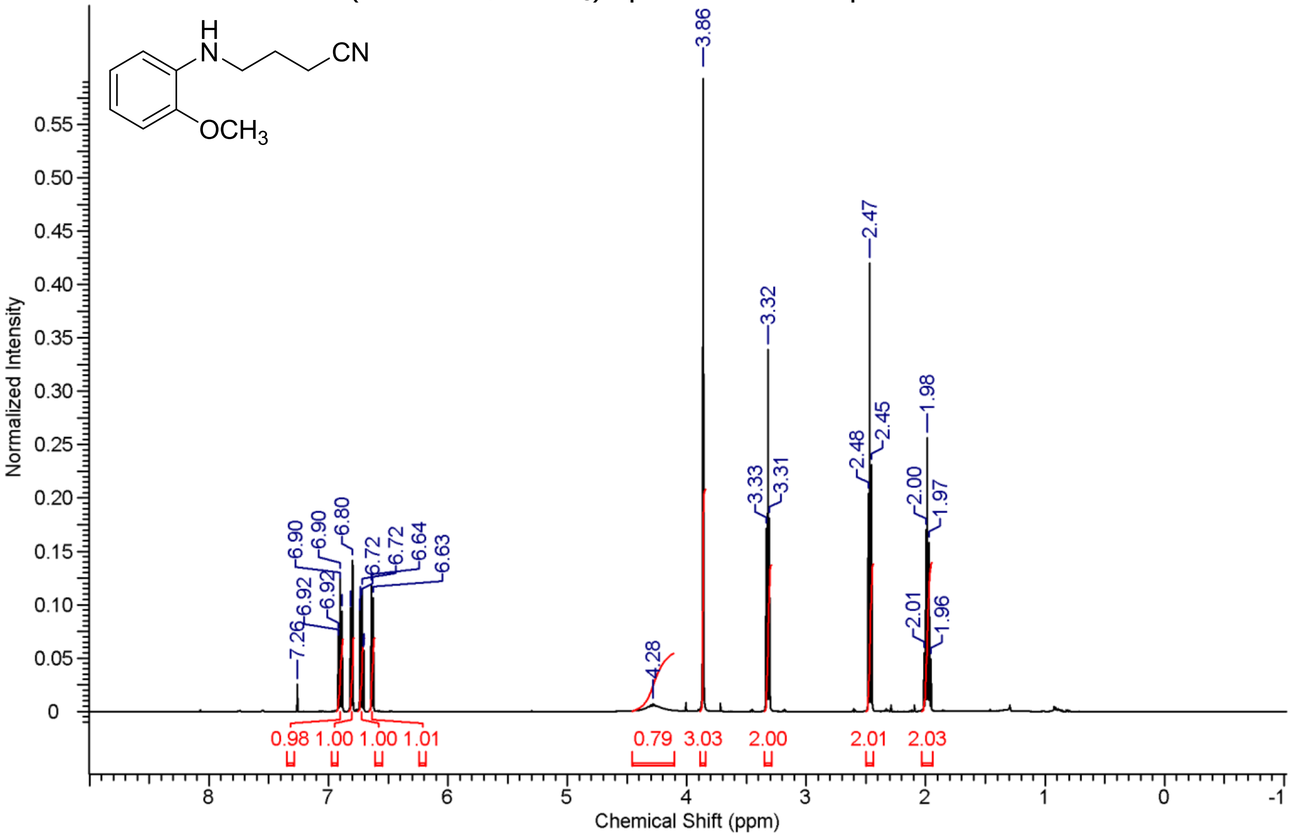

^{13}C NMR (150 MHz, $CDCl_3$) spectrum of compound **1h**

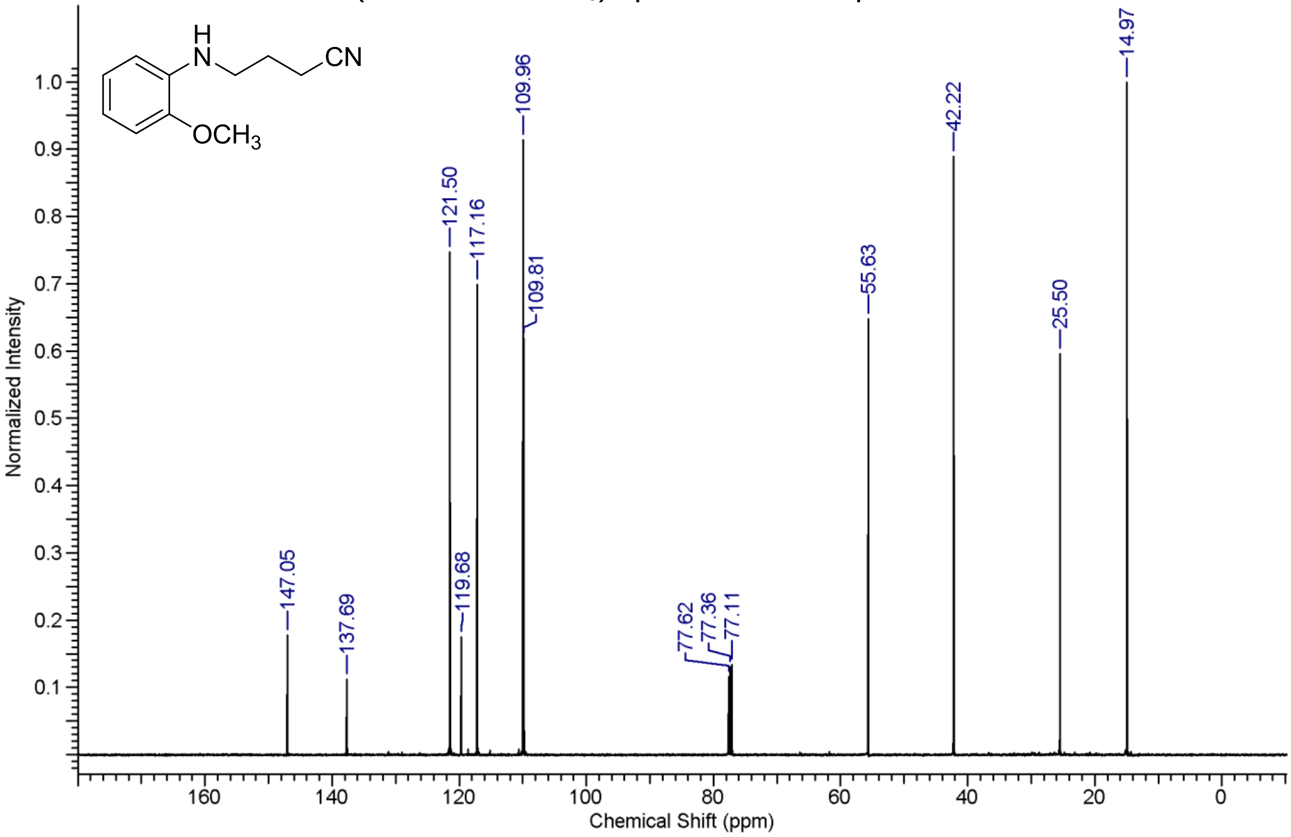

^{1}H NMR (500 MHz, $CDCl_3$) spectrum of compound **3e**

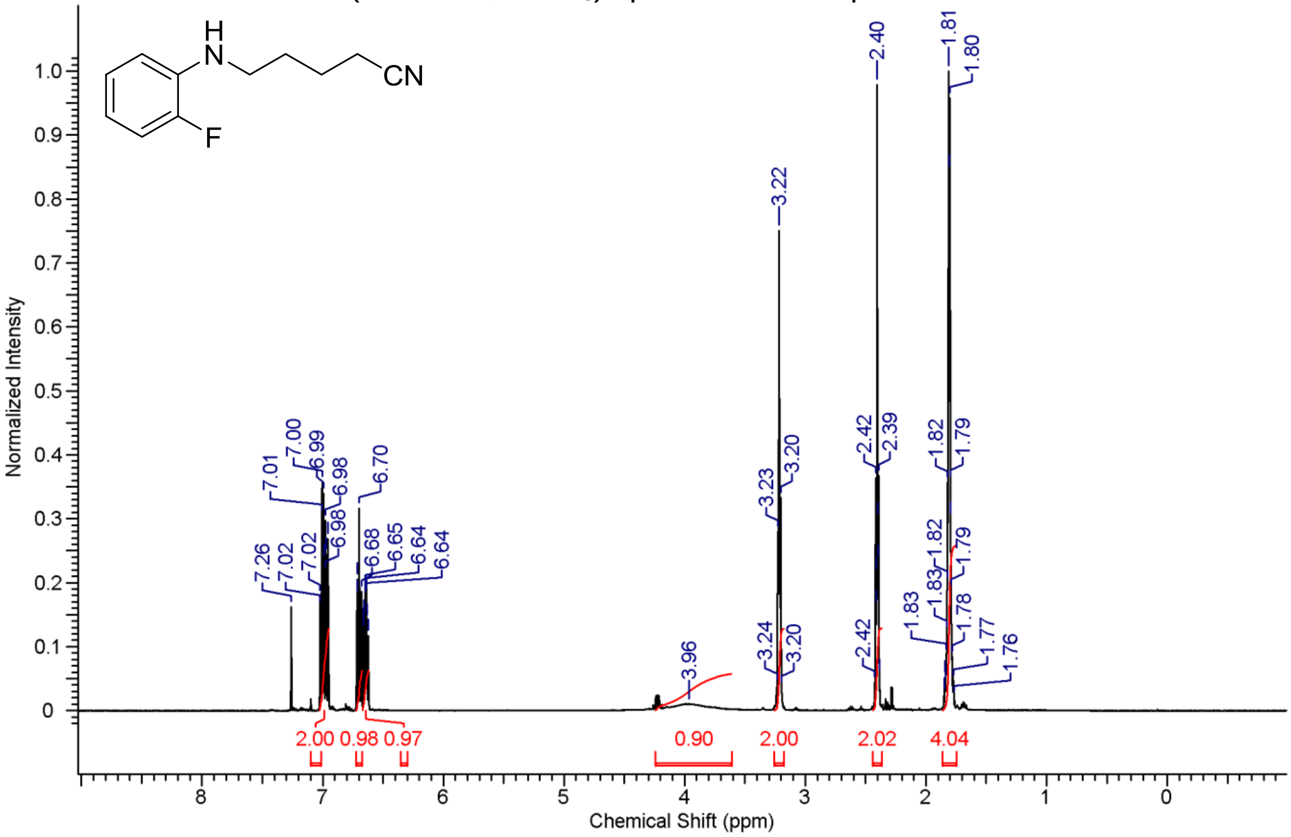

^{13}C NMR (125 MHz, $CDCl_3$) spectrum of compound **3e**

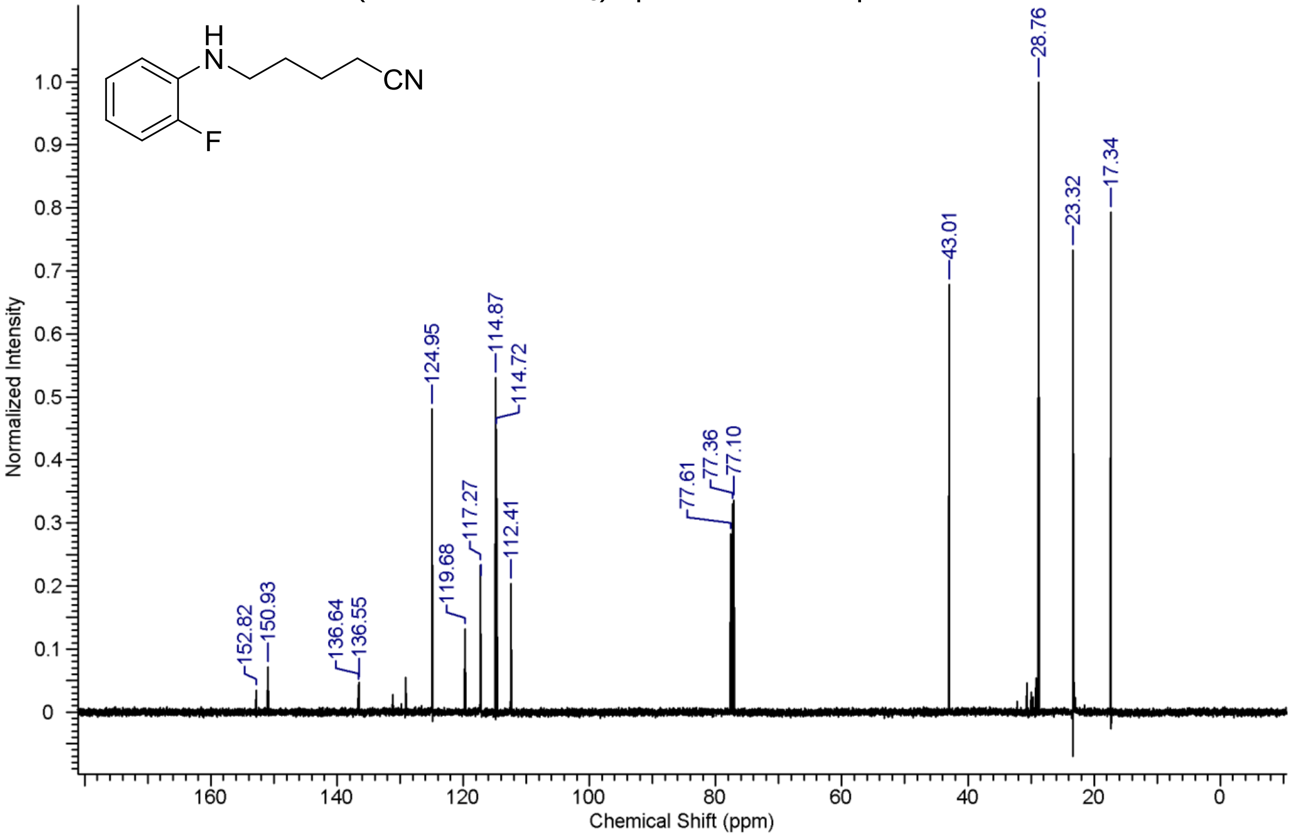

^{1}H NMR (500 MHz, $CDCl_3$) spectrum of compound **3f**

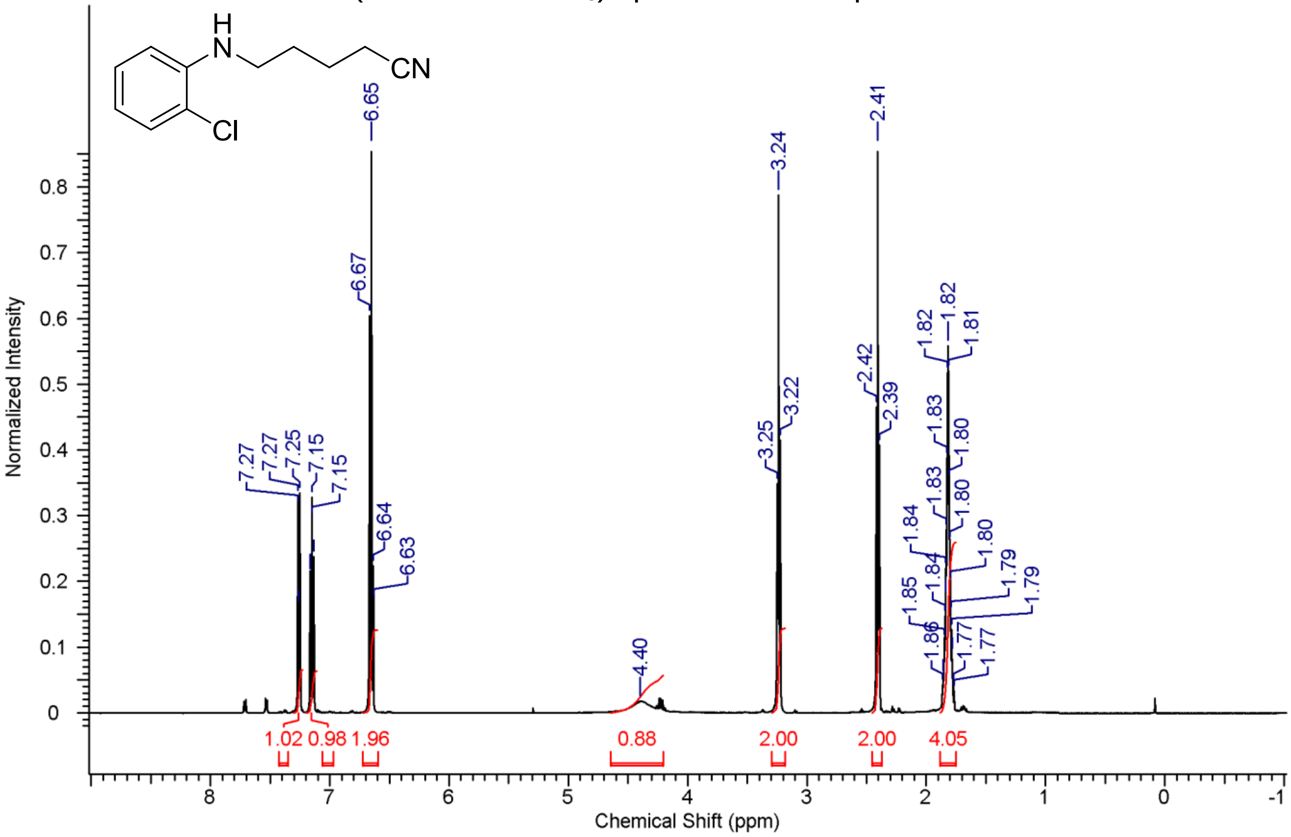

^{13}C NMR (125 MHz, $CDCl_3$) spectrum of compound **3f**

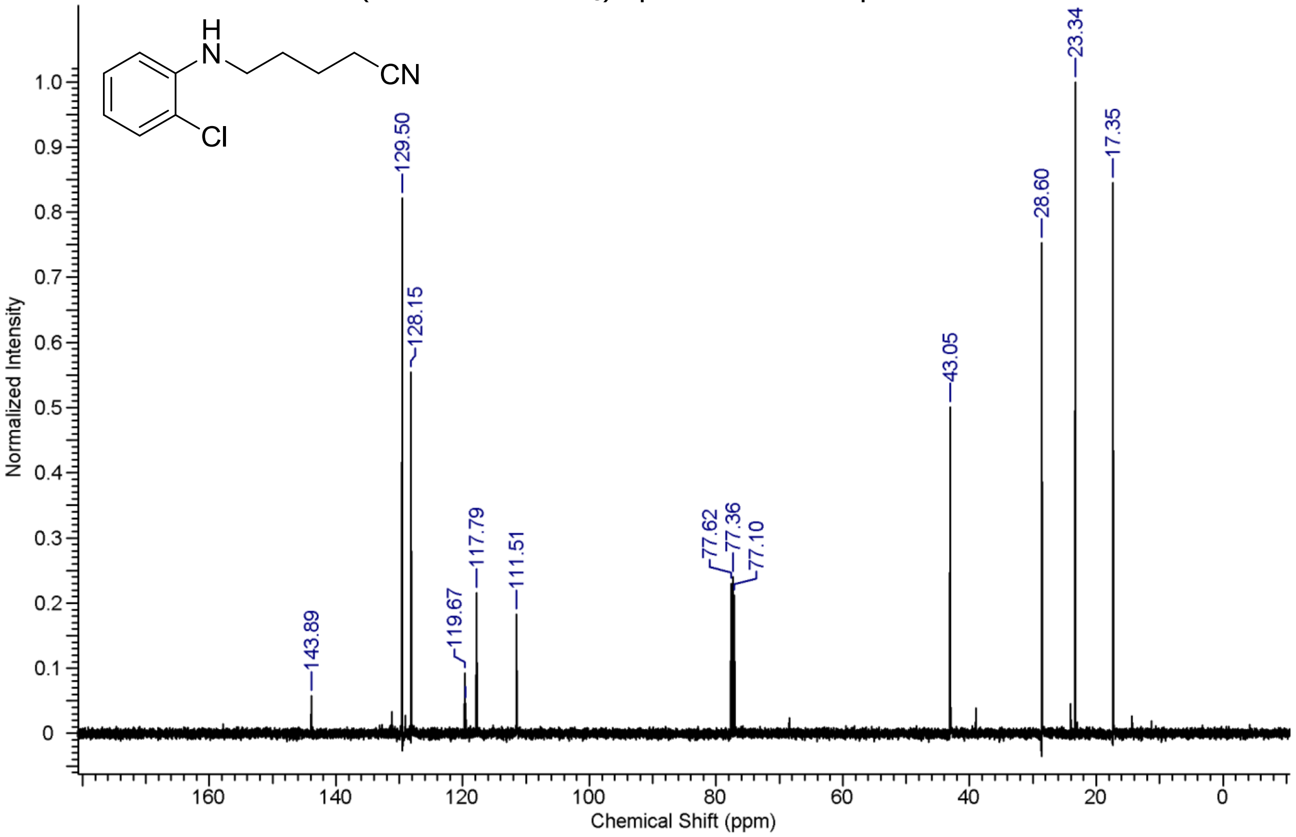

^{1}H NMR (500 MHz, $CDCl_3$) spectrum of compound **3g**

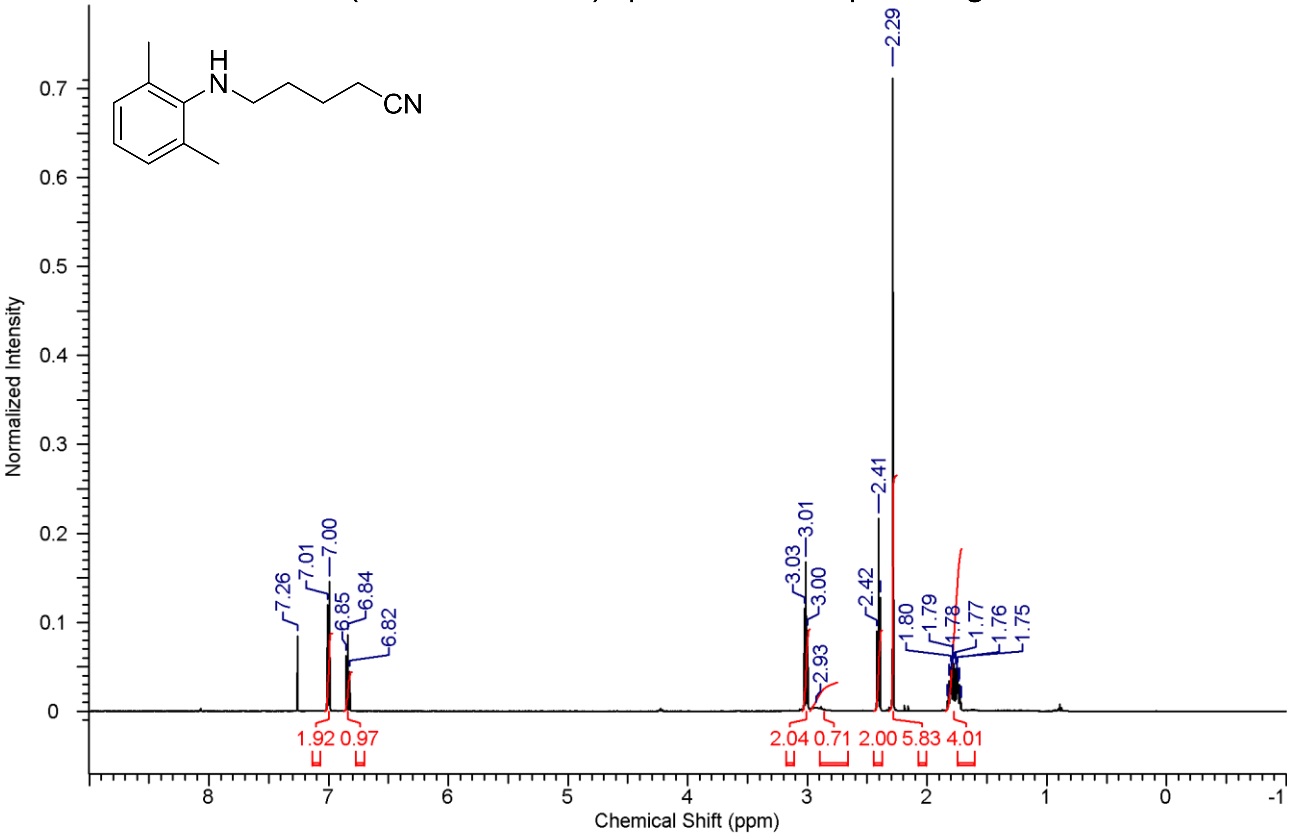

^{13}C NMR (125 MHz, $CDCl_3$) spectrum of compound **3g**

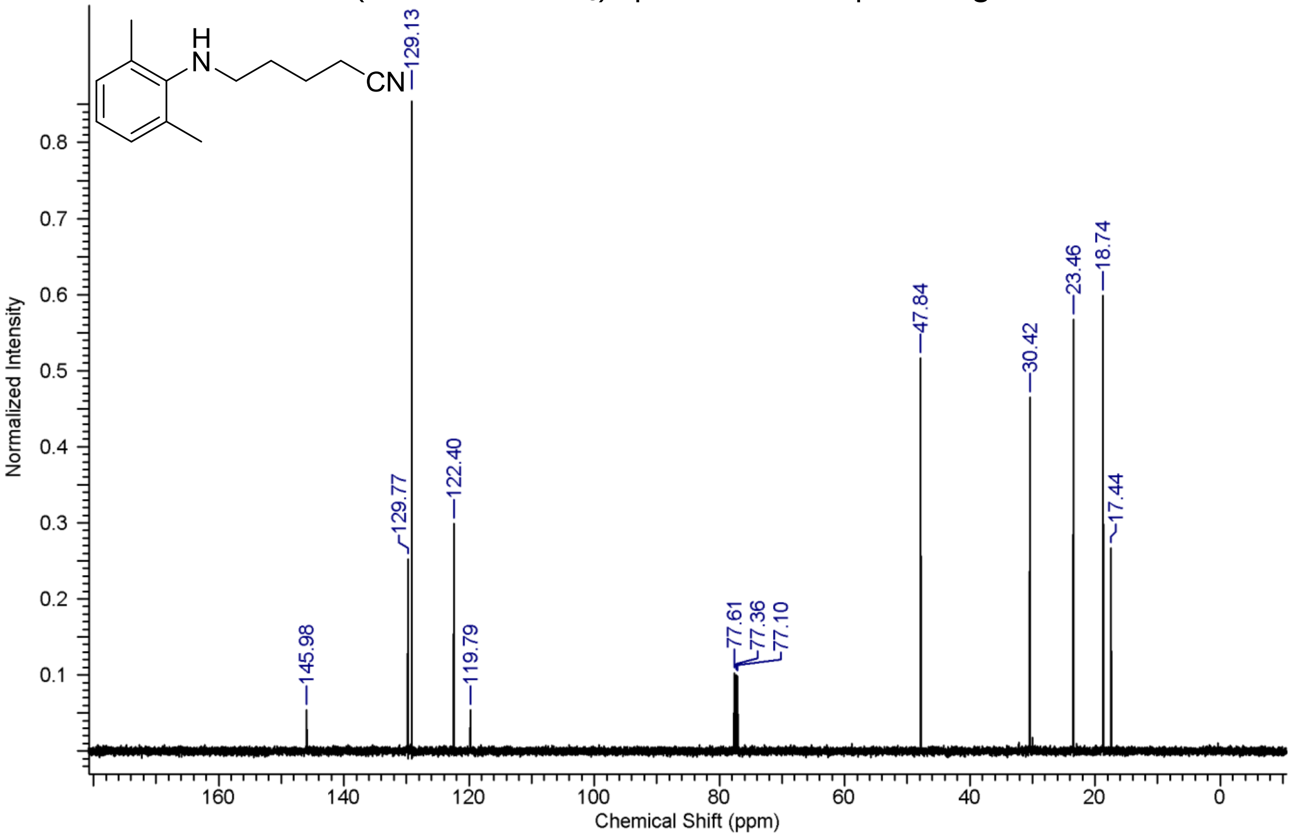

^{1}H NMR (500 MHz, $CDCl_3$) spectrum of compound **6a**

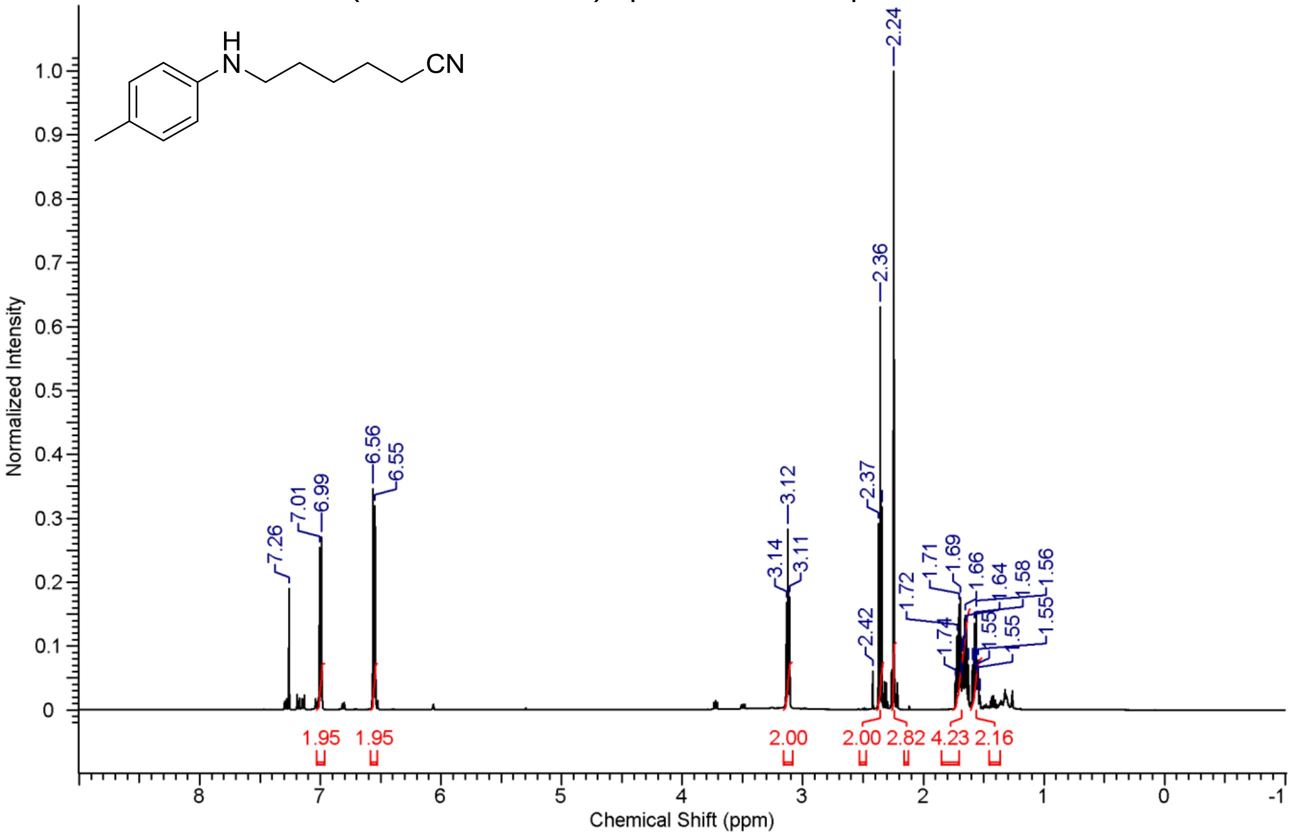

^{13}C NMR (125 MHz, $CDCl_3$) spectrum of compound **6a**

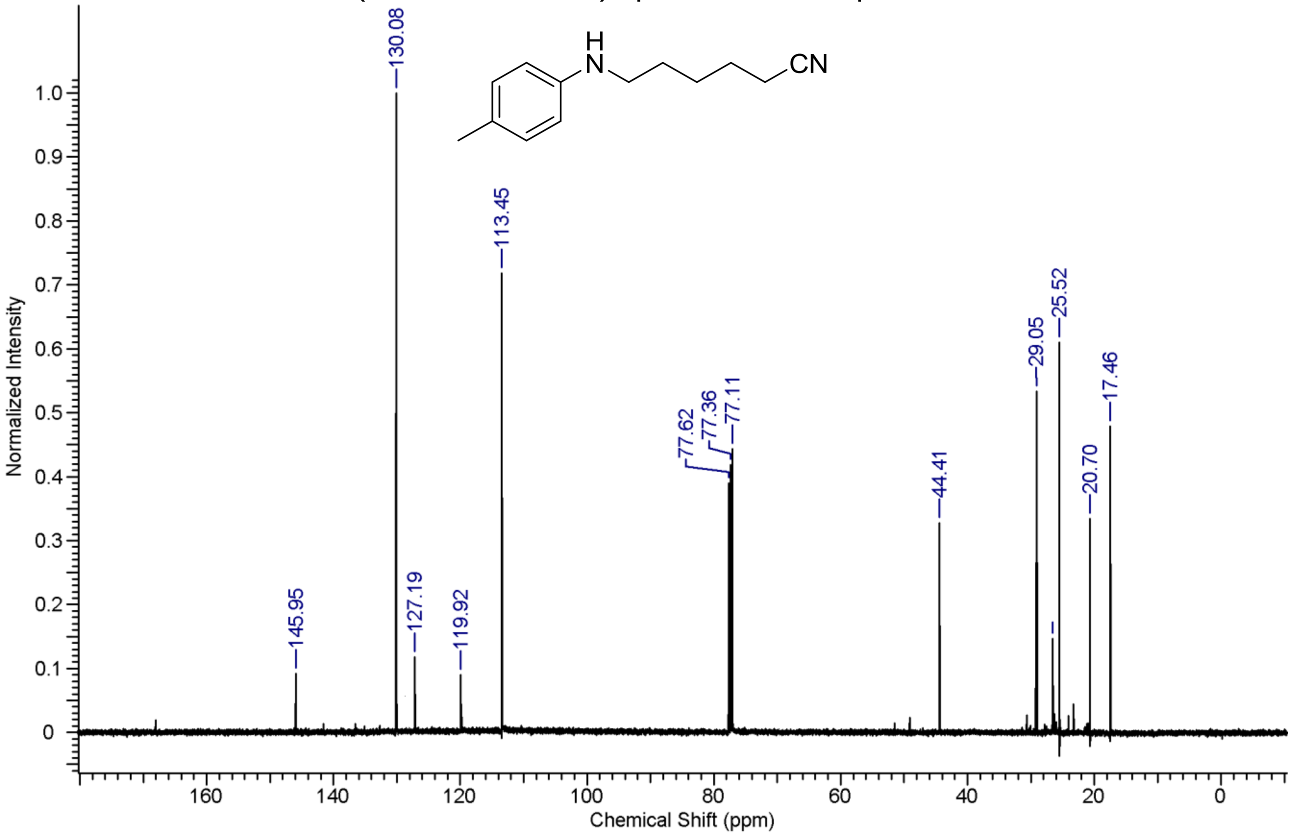

^{1}H NMR (500 MHz, $CDCl_3$) spectrum of compound **6b**

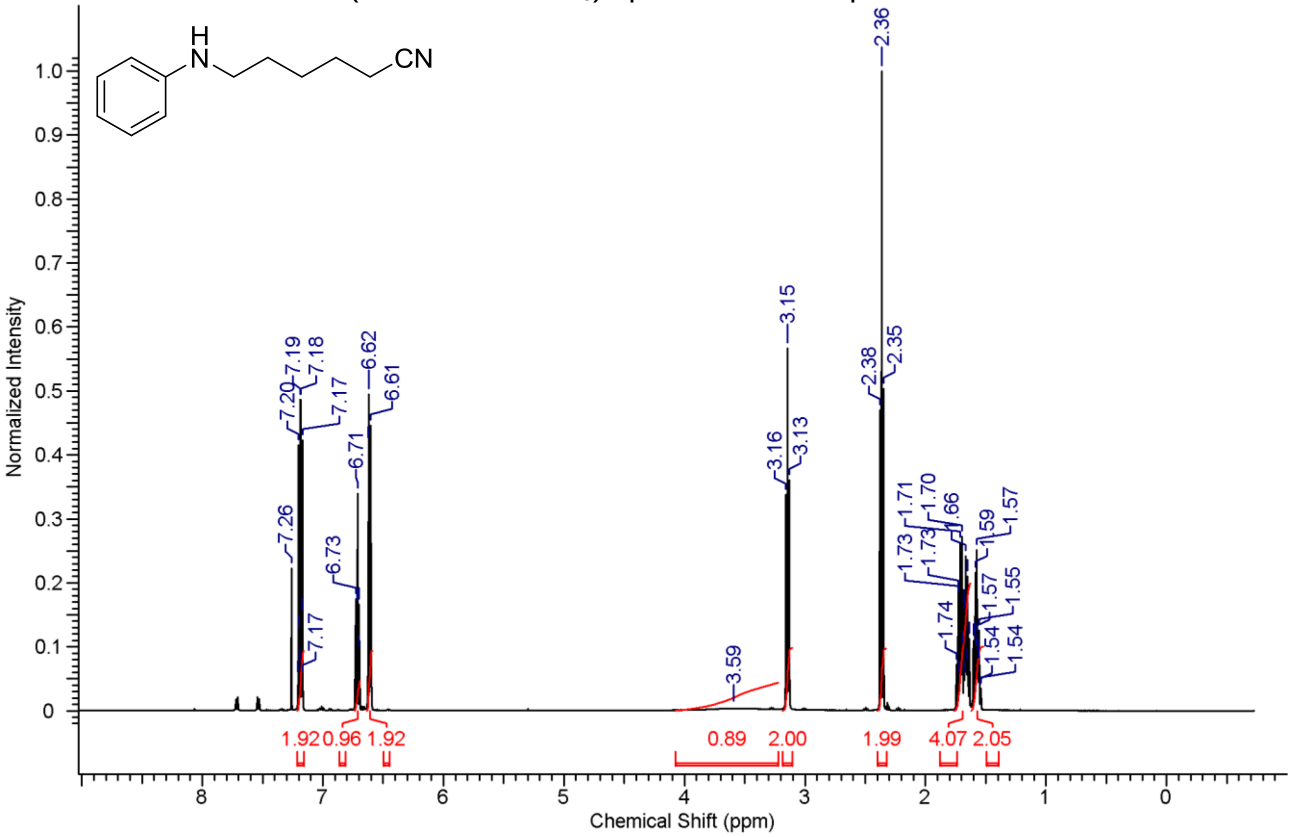

^{13}C NMR (125 MHz, $CDCl_3$)of compound **6b**

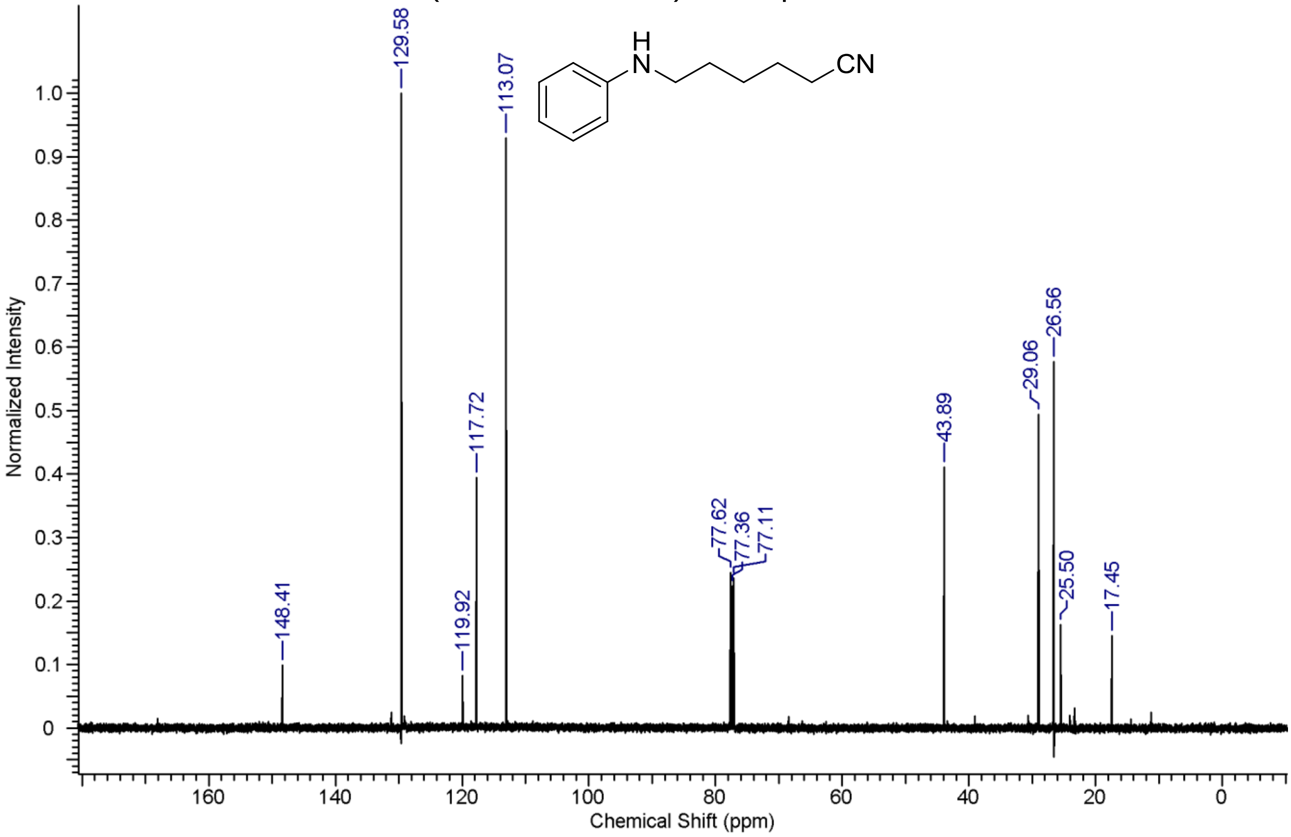

^{1}H NMR (600 MHz, $CDCl_3$) spectrum of compound **6c**

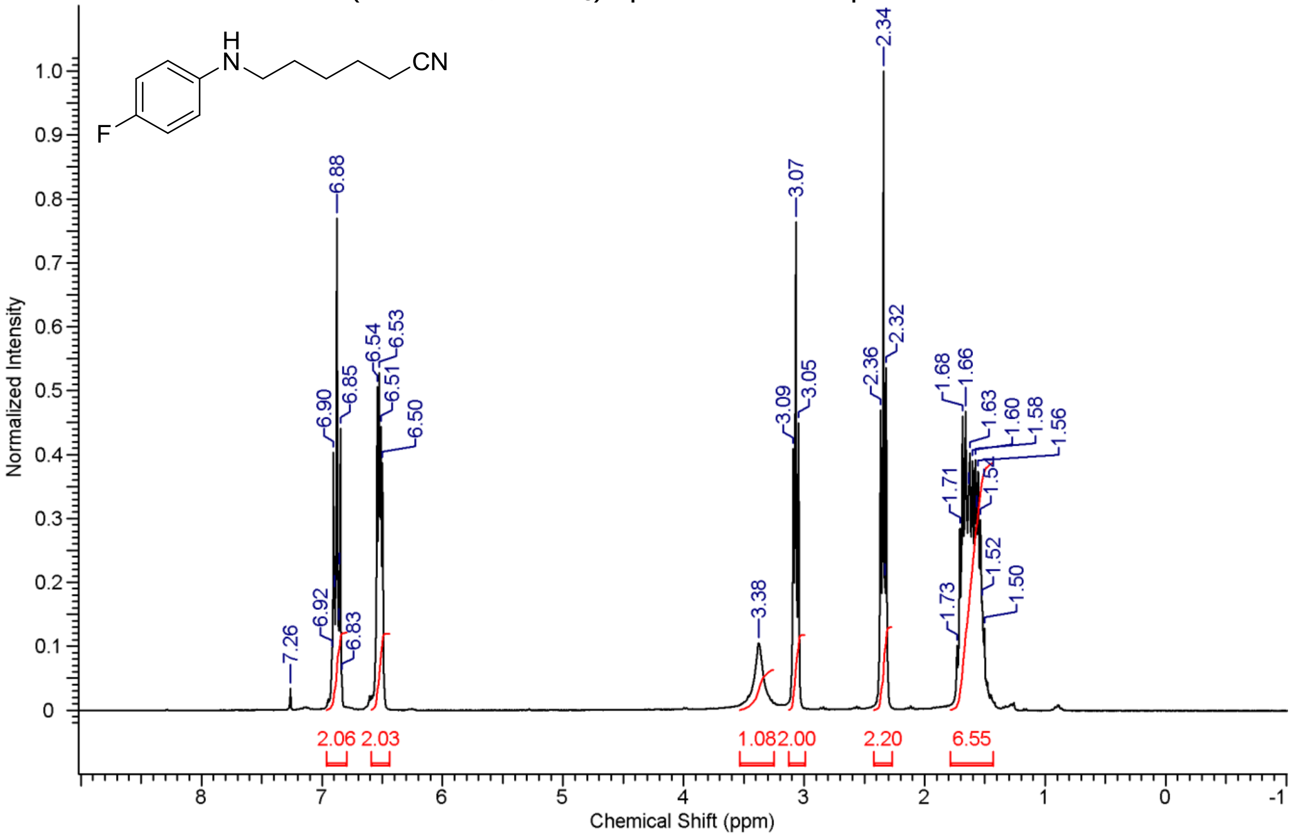

^{13}C NMR (150 MHz, $CDCl_3$)of compound **6c**

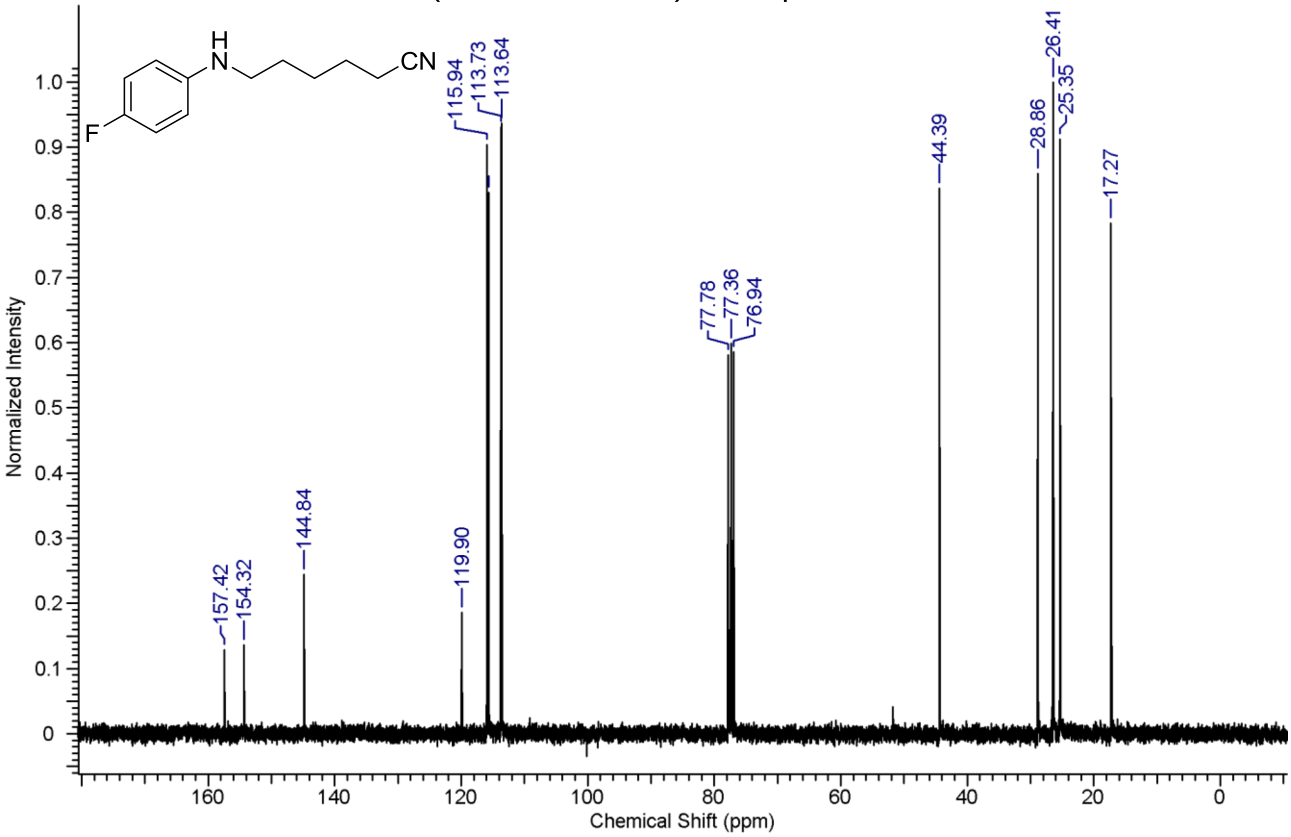

^{1}H NMR (500 MHz, $CDCl_3$) spectrum of compound **6d**

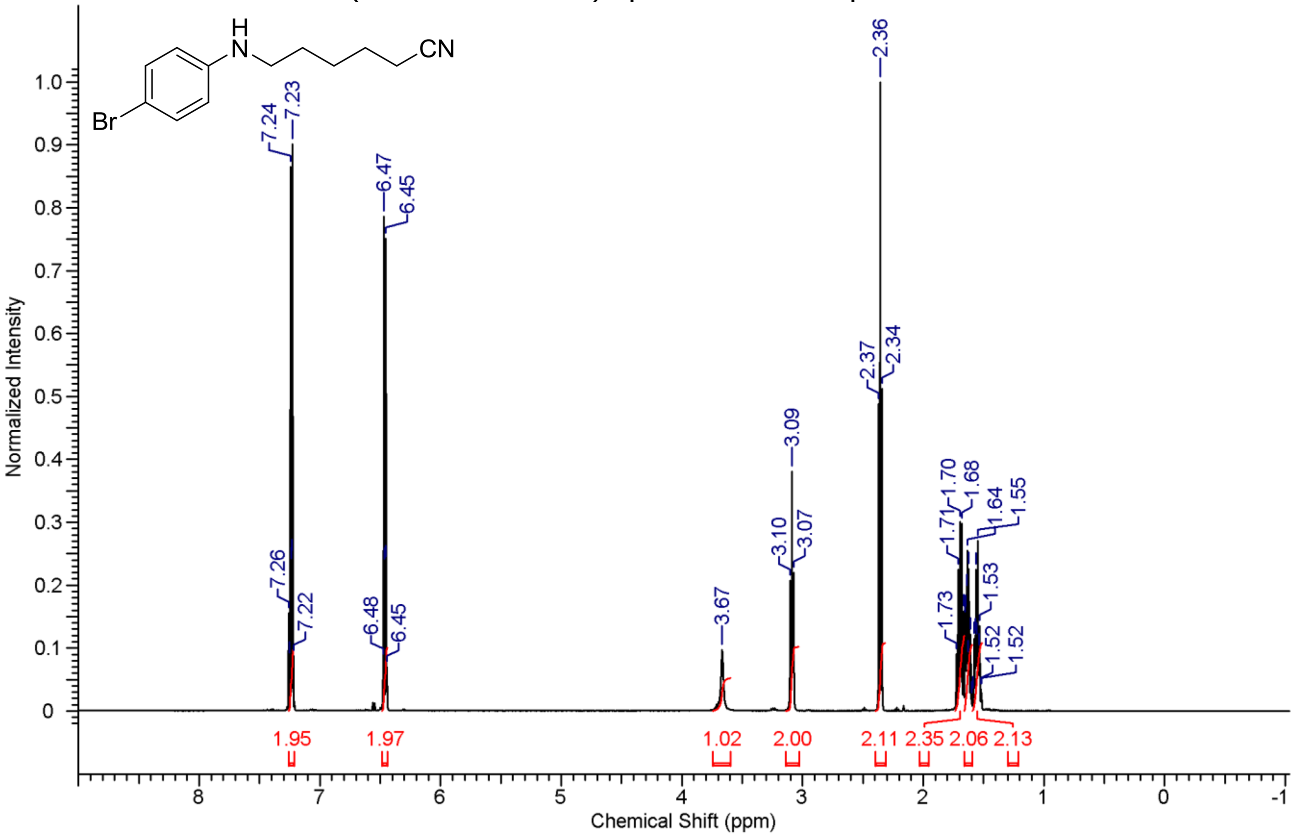

^{13}C NMR (125 MHz, $CDCl_3$) spectrum of compound **6d**

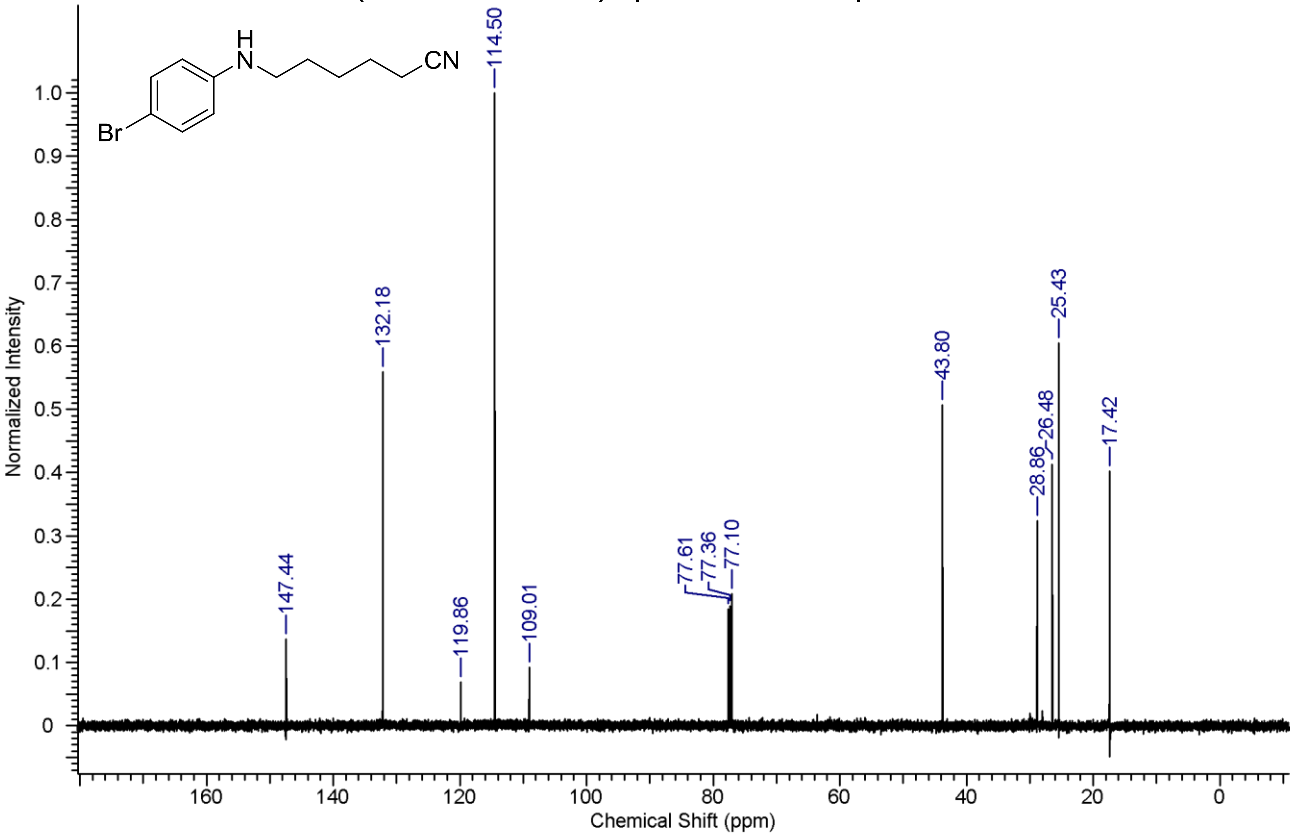

^{1}H NMR (600 MHz, $CDCl_3$) spectrum of compound **6e**

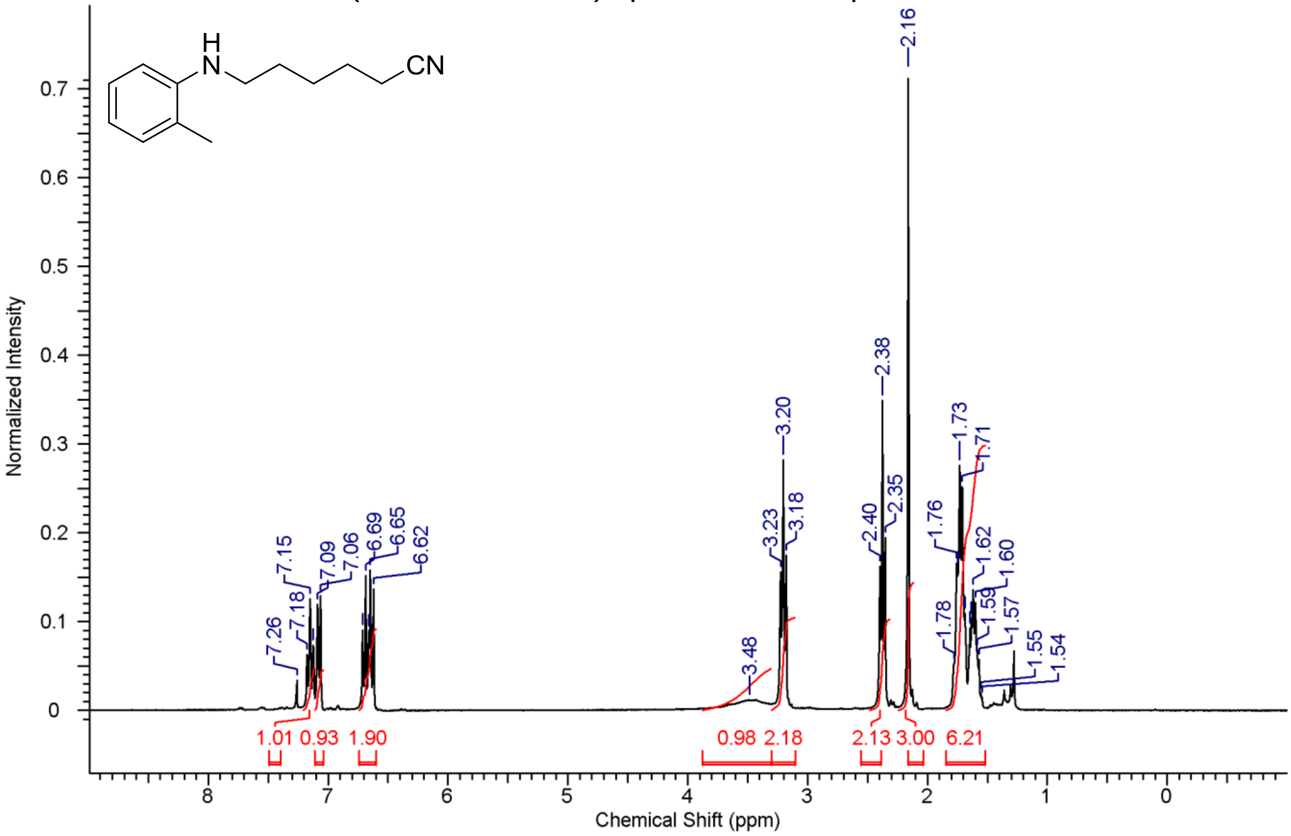

^{13}C NMR (150 MHz, $CDCl_3$) spectrum of compound **6e**

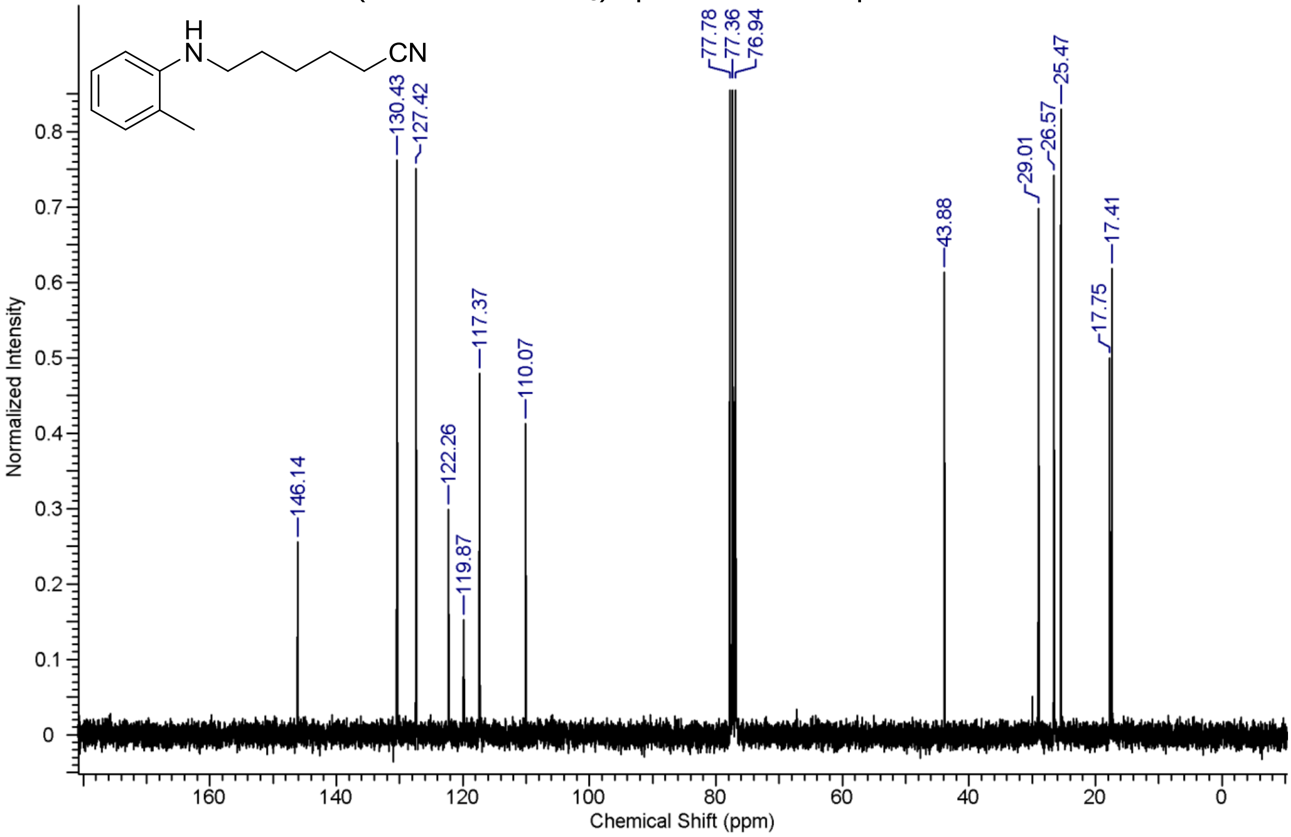

^{1}H NMR (500 MHz, $CDCl_3$) spectrum of compound **6f**

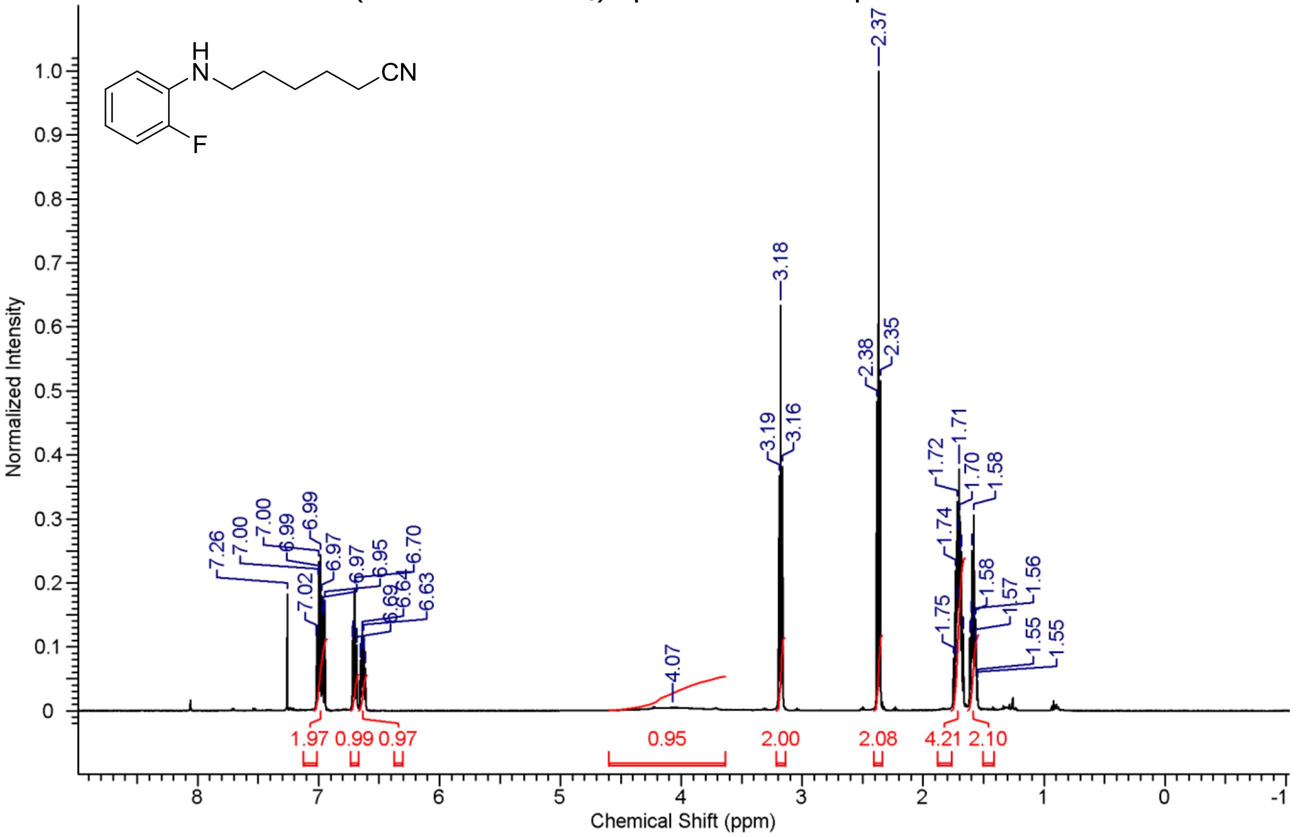

^{13}C NMR (125 MHz, $CDCl_3$) spectrum of compound **6f**

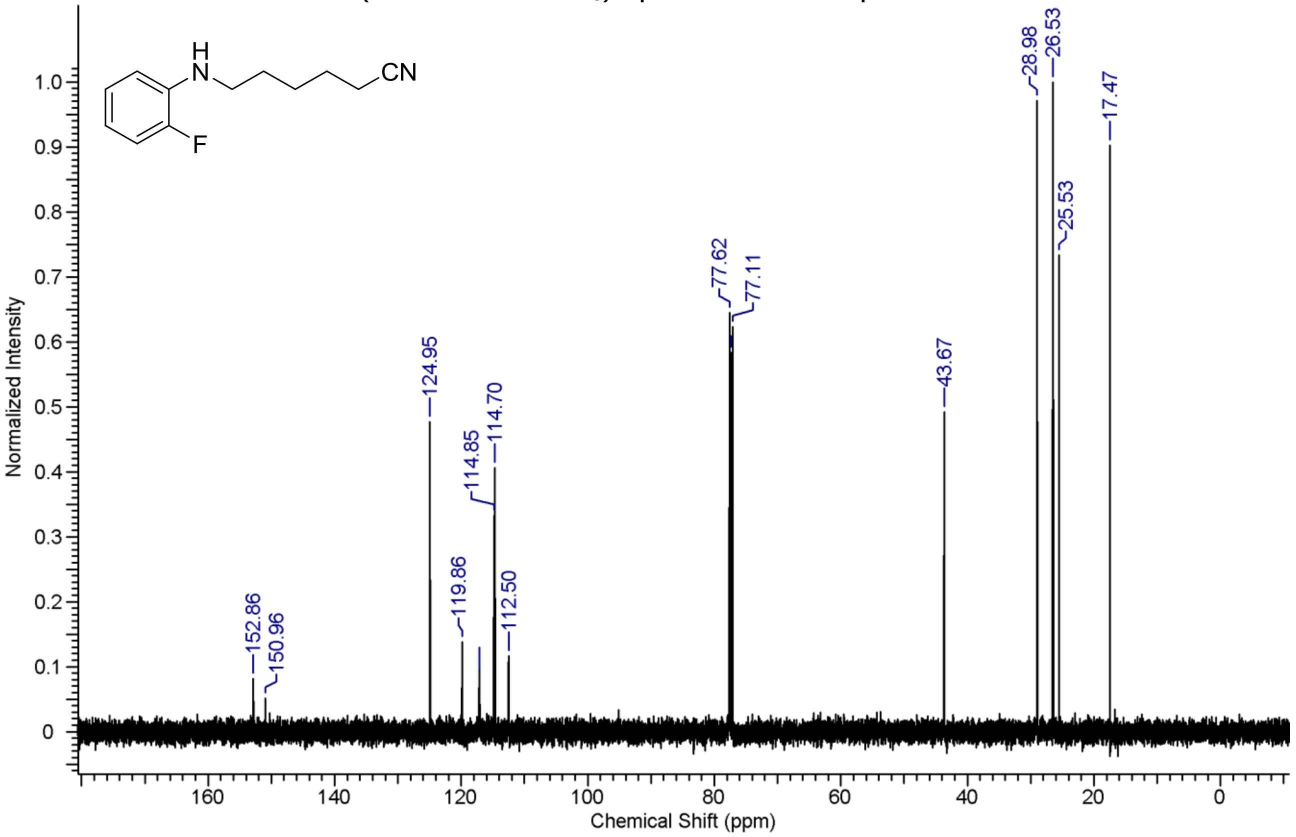

^{1}H NMR (500 MHz, $CDCl_3$) spectrum of compound **6g**

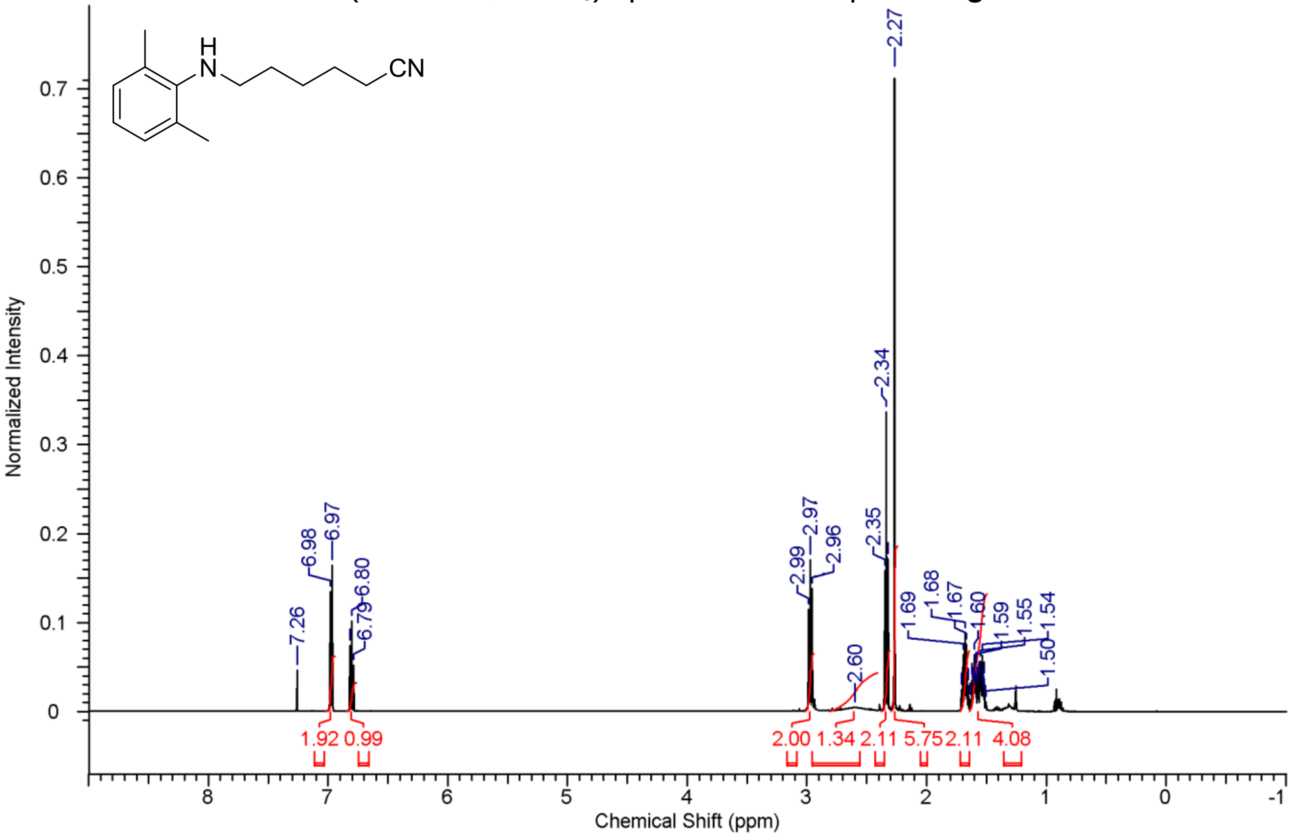

^{13}C NMR (125 MHz, $CDCl_3$) spectrum of compound **6g**

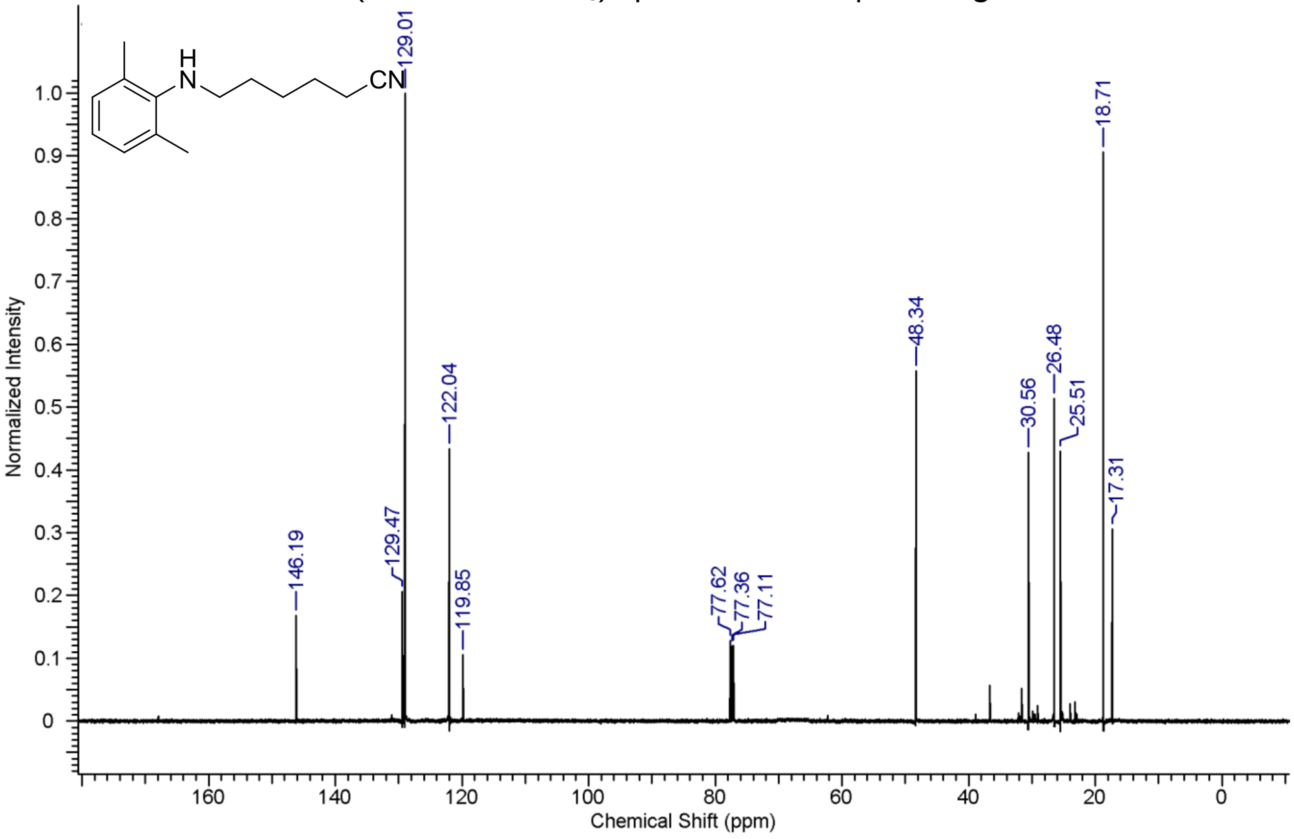

5. References:

[1] Pollmann, W; Schramm, G. *Biochim. Biophys. Acta*, **1964**, 80, 1-7.
[2] Yokoyama, M.; Yoshida, S.; Imamoto, T. *Synthesis* **1982**, 591-592.
[3] Link, N. P.; Díaz, J. E.; Orelli, L. R. *Synlett* **2009**, 5, 751-754.
[4] Díaz, J. E.; Bisceglia, J. A.; Mollo, M. C.; Orelli, L. R. *Tetrahedron Lett.* **2011**, 52, 1895-1897.
[5] Diaz, J. E.; Gruber, N.; Orelli, L. R. *Tetrahedron Lett*. **2011**, 52, 6443-6445.
